# Supplementary material for: Specialized sledge dogs accompanied Inuit dispersal across the North American Arctic
Source: Proc Biol Sci. 2019 Nov 27;286(1916):20191929. doi: 10.1098/rspb.2019.1929 (PMC6939252; doi:10.1098/rspb.2019.1929)
Supplement: Supplementary text [file rspb20191929supp1.docx]

Supplementary Information for

Specialised sledge dogs aided Inuit dispersal across the North American Arctic

Carly Ameen, Tatiana R. Feuerborn, Sarah K. Brown, Anna Linderholm, Ardern Hulme-Beaman, Ophélie Lebrasseur, Mikkel-Holger S. Sinding, Zachary T. Lounsberry, Audrey T. Lin, Martin Appelt, Lutz Bachmann, Matthew Betts, Kate Britton, John Darwent, Rune Dietz, Merete Fredholm, Shyam Gopalakrishnan, Olga I. Goriunova, Bjarne Grønnow, James Haile, Jón Hallsteinn Hallsson, Ramona Harrison, Mads Peter Heide-Jørgensen, Rick Knecht, Robert J. Losey, Edouard Masson-MacLean, Thomas H. McGovern, Ellen McManus-Fry, Morten Meldgaard, Åslaug Midtdal, Madonna Moss, Iurii G. Nikitin, Tatiana Nomokonova, Albína Hulda Pálsdóttir, Angela Perri, Aleksandr N. Popov, Lisa Rankin, Joshua D. Reuther, Mikhail Sablin, Anne Lisbeth Schmidt, Scott Shirar, Konrad Smirarowski, Christian Sonne, Mary C. Stiner, Mitya Vasyukov, Catherine F. West, Gro Birgit Ween, Sanne Eline Wennerberg, Øystein Wiig, James Woollett, Love Dalén, Anders J. Hansen, Tom Gilbert, Benjamin Sacks, Laurent Frantz, Greger Larson, Keith Dobney, Christyann M. Darwent, Allowen Evin

Corresponding Author: Carly Ameen

Email: [C.Ameen@exeter.ac.uk](mailto:C.Ameen@exeter.ac.uk)

This PDF file includes:

Supplementary text

Figs. S1 to S17

Captions for databases S1 to S6

References for SI reference citations

Other supplementary materials for this manuscript include the following:

Datasets S1 to S6

Table of Contents:

[**\section*{A note on cultural terminology:}**](#_yxgt2ku6oqh6) **2**

[**\section*{A note on modern and historical samples:}**](#_1iy8ran7vnuy) **3**

[**\section*{Ancient DNA Methods}**](#_1yfyvxcy5fd7) **4**

[\subsection*{Sampling and DNA Extraction}](#_7x2gt1g3dgq7) 4

[\subsection*{DNA Library Building}](#_3abyclqtz2al) 5

[\subsection*{Sequencing and Data Preparation}](#_4e2kaeotbb0z) 7

[\subsection*{Maximum Likelihood Tree Construction}](#_5aw0okwnh0n4) 7

[\subsection*{Bayesian Skyline Analysis}](#_23e42xscsmf) 8

[\subsection*{Haplotype Determination}](#_caes7o48ruq2) 9

[**\section*{Ancient DNA Expanded Results}**](#_g0dqdmjng0tb) **9**

[\subsection*{A-clade Subclades and Haplotypes}](#_khk8eqka0777) 9

[\subsection*{Demographic Histories of Inuit and Historical Dogs}](#_kz1b446fvz6c) 11

[\subsection*{Analyses of AL2797 Nuclear Genome}](#_8r0lfd3gzto7) 12

[**\section*{Geometric Morphometrics Methods}**](#_tkrs3w5wxbtl) **13**

[\subsection*{2D hemi-mandible and lower M1}](#_8chsfwno4jsz) 13

[\subsection*{Building 3D models of crania using photogrammetry}](#_io0zcfxu7z8g) 13

[\subsection*{GMM Statistical Analysis}](#_hj4n3o6zby9s) 14

[**\section*{Stable Isotope Analysis}**](#_oja877dej21p) **15**

[\subsection*{Stable Isotope Analysis Methods}](#_7vkmc7pt17vh) 15

[\subsection*{Stable Isotope Analysis Results}](#_jhcu0hszoqvt) 16

[**\section*{Archaeological Site Descriptions}**](#_8md0r567h4u8) **16**

[\subsection*{North American Paleo-Inuit sites}](#_eidg1xttx1m4) 16

[\subsection*{North American Inuit Sites}](#_hq341rvljyr8) 27

[\subsection*{Siberian Archaeological Sample Descriptions}](#_mkv7419trjz) 50

[\subsection*{Icelandic and Norse Greenlandic Archaeological Sample Descriptions}](#_13zkuspt28uk) 54

[\subsection*{Museum and Ethnographic Sample Descriptions}](#_bfg45uarfa6p) 59

[**SI Figure Captions**](#_abfy09tykcs2) **66**

[**Captions for Databases S1 - S7**](#_z5i7bz6bz9bs) **67**

[**SI References**](#_5qqeh55dp325) **68**

## \section*{A note on cultural terminology:}

The terms ‘Inuit’ and ‘Paleo-Inuit’ are used throughout this manuscript in the following way; dogs from archaeological sites from the more recent archaeological traditions of the North American Arctic, from ~ 1000 BP onwards, are referred to collectively as ‘Inuit’ and include specimens from sites attributed to Eskimo, Neo-Eskimo, Northern Maritime, Thule, Old Bering Sea (OBS), Punuk, and Birnirk cultures. The Northern Maritime tradition includes OBS, Punuk, Birnirk, and Thule, with the development of toggling harpoons and drag floats originating with OBS ~2000 BP in the Bering Strait region. The Thule migration out of Alaska into the Eastern Arctic of Canada and Greenland is generally agreed to date to ca. AD 1300 (Friesen and Arnold 2008), thus “Inuit” origins are much older in the Bering Strait than in the Eastern Arctic. The term ‘Paleo-Inuit’ here refers to dogs associated with Arctic traditions prior to ~1000 BP, and includes specimens from sites attributed to the Allutiiq, Arctic Small Tool, Denbigh Flint Complex, Choris, Dorset, Ipiutak, Kachemak, Norton, and Saqqaq cultures. The use of the term ‘Paleo-Inuit’ should not be considered as acknowledgment of any direct ancestral relationship between these groups (human or canid) and the Inuit culture. In some cases, there is no direct contact between the Inuit peoples and those who occupied certain parts of this macro-region prior to ~ 1000 BP. However, the Inuit migration represents a pivotal moment in the bio-cultural history of this entire region, and in relation to the dogs included in this analysis, the samples classified as ‘Paleo-Inuit’ should be interpreted only as chronologically predating the Inuit period.

The cultural terminology used in this manuscript follows the advice of the Inuit Circumpolar Council ([ICC Resolution 2010 – 01](http://www.inuitcircumpolar.com/uploads/3/0/5/4/30542564/iccexcouncilresolutiononterminuit.pdf)), which represents the majority of indigenous groups of the Arctic macro-region from Chukotka to Greenland. A summary of these terms and their relationship to Arctic archaeological research is outlined by Friesen [(T. M. Friesen 2015)](https://paperpile.com/c/GftuPk/dq5oX), and the separation of dogs sampled here into Inuit and Paleo-Inuit groups is supported by both the archaeological record and ancient DNA analysis of human remains [(Raghavan et al. 2014; Duggan et al. 2017; Flegontov et al. 2017; Jensen 2016; Tackney et al. 2019)](https://paperpile.com/c/GftuPk/y69GU+1MQ4W+spLl6+uvQRw+u5yb9). We recognise that no simplified naming system is completely suitable to cover the multiple, diverse and unique indigenous groups that make up both the modern and archaeological occupants of the North American Arctic and Subarctic regions. We have adopted the above classification system which is supported by modern Indigenous representatives of the region as well as regional archaeological experts, in an attempt to find a suitable (although not perfect) terminology for discussing these large scale, pan-regional trends.

## \section*{A note on modern and historical samples:}

Throughout this manuscript, ‘Modern’ refers to samples collected from living dogs within the last two decades. Samples referred to as historical include those which are older than 20 years, collected during ethnographic expeditions from living dogs, or skeletal remains of dogs from surface contexts with no known archaeological dates. Samples categorised as historical may represent dogs of archaeological origin as a result of their surface context rather than sub-surface and the slow rate of sediment deposition in the region; however, without radiocarbon dating the age of the samples cannot be precisely determined. Samples of historical origin are separated from Inuit samples in this study due to the contact between Europeans and Arctic communities introducing European dogs to the region. Modern, historical, and Inuit age dogs from the sledge district of Greenland represent the traditional Greenland Sledge Dog. To be comparable with samples across the Arctic region, Greenland Sledge Dogs are divided by the age of the sample. The Canadian Inuit Dog and Greenland Sledge Dog are considered to represent the same breed, with the key differences being the location and size of the population [(S. K. Brown et al. 2015)](https://paperpile.com/c/GftuPk/cBWRk). Similarly, this may be the case for historical dogs in Siberia related to the Siberian husky and Alaskan Malamutes with historical Alaskan dogs; however, they show a greater extent of admixture with Eurasian dog populations than Greenland Sledge Dogs [(S. K. Brown et al. 2015)](https://paperpile.com/c/GftuPk/cBWRk). Alternatively, the Alaskan Husky represents a breed originating after contact between native Alaskans and Europeans with the crossing of Alaskan village dogs, imported Siberian Huskies, and European dogs producing Alaskan Huskies [(S. K. Brown et al. 2015)](https://paperpile.com/c/GftuPk/cBWRk).

## \section*{Ancient DNA Methods}

All samples used in the study were sampled, extracted, and setup for PCR in laboratories dedicated specifically to working with genetic samples of ancient origin, using appropriate sterile techniques and equipment, see SI Table 1 [(A. Cooper and Poinar 2000; Gilbert et al. 2005)](https://paperpile.com/c/GftuPk/Zwu5M+BThHl). Ancient DNA lab work was conducted in four facilities, the Centre of GeoGenetics at the Natural History Museum for Denmark, the Swedish Natural History Museum, the University of Oxford’s PalaeoBarn, or the Veterinary Genetics Laboratory (VGL) ancient DNA facility at the University of California, Davis (UC Davis).

### \subsection*{Sampling and DNA Extraction}

*\subsubsection*{Centre for GeoGenetics and Swedish Natural History Museum}*

For teeth and bone, all specimens were initially ‘cleaned’ through the removal of the surface through superficial drilling with a Dremel drill. Following the removal of the external layer the desired 30-80mg of bone power was drilled out of the bonee. For the samples which were derived from teeth the outer surface was removed and the cementum layer of the tooth was drilled to a powder for subsequent extraction, amounting to between 15 and 30 mg per sample. 51 samples were skin samples originating from clothing items or museum hides, the hairs on the skin were removed with a sterile scalpel from the subsample taken from the hide of approximately 30 mg. Bone, tooth, and skin samples extracted at the Swedish Natural History Museum underwent a pre-digestion step consisting of a 45 minute incubation in 315 µL of 0.5M EDTA and 7.5 µL of 10 ng/µL proteinase K. After incubation the samples were spun in a centrifuge at 13,000 rpm for 5 minutes, following which the supernatant was removed. From this stage the remaining undigested material was extracted overnight with 630 µL of 0.5M EDTA, 70 µL of 1M UREA, and 15 µL of 10 ng/µL proteinase K as per [(Ersmark et al. 2015)](https://paperpile.com/c/GftuPk/WUa6n). The skin samples were also incubated overnight in a lysis buffer as per [(Gilbert et al. 2007)](https://paperpile.com/c/GftuPk/uLvgH). Purification of extracts was performed according to [(Ersmark et al. 2015)](https://paperpile.com/c/GftuPk/WUa6n).

*\subsubsection*{University of California, Davis}*

UC Davis bone and tooth specimens were extracted via the silica extraction method [(Rohland and Hofreiter 2007; Rohland, Siedel, and Hofreiter 2010)](https://paperpile.com/c/GftuPk/sGaW6+2uNo6) with slight modifications, as per [(Sarah K. Brown, Darwent, and Sacks 2013)](https://paperpile.com/c/GftuPk/61pmw). The modifications include, 1) prior to lysis, samples were subject to a decalcification solution in 2 ml of 0.5 M EDTA solution and kept rotating at room temperature for two days, and 2) subsequent to extraction, DNA was ethanol precipitated to remove inhibitors as per [(Kemp, Monroe, and Smith 2006)](https://paperpile.com/c/GftuPk/A3FBd), and eluted in a final volume of 100 µl.

*\subsubsection*{University of Oxford’s PalaeoBARN}*

All the bone or teeth samples were “cleaned” by removing the outer surface layer, after this a small piece was cut and the bone/tooth was pulverised using a mixer mill generating around 50-70 mg powder, all of this was undertaken in a separate room in the dedicated aDNA lab. Extraction was carried out using the silica method following the Dabney extraction protocol [(Dabney et al. 2013)](https://paperpile.com/c/GftuPk/BiiZx) but with the addition of a 30 minutes pre-digest stage following [(Damgaard et al. 2015)](https://paperpile.com/c/GftuPk/bA0Oq).

### \subsection*{DNA Library Building}

*\subsubsection*{Centre for GeoGenetics and Swedish Natural History Museum}*

Libraries were built at the Swedish Natural History Museum using the blunt ending single‐tube library preparation, ‘BEST’ method specifically designed for working with degraded DNA [(Carøe et al. 2018)](https://paperpile.com/c/GftuPk/IMJda). The loss of DNA during the library build process is reduced through the limitation in the number of purifications the sample undergoes. The extract initially undergoes blunt end repair followed by a denaturing step at 65°C, without purification the sample proceeds to the adapter ligation step where Illumina adapters are ligated to the ends of the DNA fragments. Finally, after the adapter ligation reaction has incubated, the nicks in the strands are filled in and following incubation each sample is then purified using Qiagen MinElute columns.

*\subsubsection*{University of California, Davis}*

The libraries constructed at UC Davis were prepared using the NEBNext Ultra DNA kit (New England Biosystems). The manufacturer’s protocol was modified by reducing the reaction volume in half, including the volume of DNA. Library preparation was conducted in a dedicated laboratory equipped with a laminar flow hood with UV lights for sterilization. DNA was subject to end repair and dA-tailing and incubated for 30 minutes at 20°C and then 30 minutes for 65°C. Next, ligation to the NEBNEXT Ultra Universal adapter was performed by adding 32.5 µL of the end repair/dA-tailing reaction with 1.25 µL of 1.5 µM adapters (diluted tenfold for ancient DNA), 0.5 µL Ligation enhancer, and 7.5 µL of Blunt/TA Ligase Master Mix. This reaction was incubated at 20°C for 15 minutes. USER enzyme (1.5 µL) was added to the reaction to cleave the loop adapters, incubated at 37°C for 15 minutes, and then purified with 1X Ampure beads according to protocol (no size selection). Libraries were uniquely barcoded through PCR amplification with indexing primers by mixing 11.5 µL of adapter ligated DNA with 12.5 µL Kapa Biosystems Uracil + master mix (Kapa: Woburn, MA), and 0.5 µL of each 25 µM primer (Universal NEB primer and Indexed primer (Supplemental Appendix A). The PCR cycling conditions were as follows: 98°C for 45s; 18 cycles of 98°C for 15s, 65°C for 30s, 72°C for 30s; 72°C for one minute. Libraries were then gel electrophoresed and quantified using a Qubit fluorometer (Life technologies; Grand Island, NY). PCR amplified libraries were then purified with 1X Ampure beads and eluted in 33 µL of 10mM Tris-HCl. Cleaned libraries were then quantified using a Qubit fluorometer (Life technologies; Grand Island, NY).

Target enrichment for the domestic dog mitochondrial genome was performed on the amplified and cleaned library using MYcroarray’s custom MYBaits enrichment kit. The custom RNA baits were developed from the *Canis familiaris* reference mitochondrial genome [(Kim et al. 1998)](https://paperpile.com/c/GftuPk/hTu0W) in 4x tiling of 80mer bases. Forty eight uniquely barcoded DNA samples were pooled in equimolar amounts (10 ng each, 480 ng total), and concentrated in a speed vacuum until dry and then reconstituted in 3.4 µL of DI RNase free water. Concentrated DNA libraries were hybridized according to manufacturer’s instructions, in a final concentration of 5.2 x SSPE, 5.2 x Denhardt’s, 5mM EDTA and 0.1\% SDS. This solution was incubated with the DNA libraries at 48°C for 37.5 hours. Hybridized DNA was then immobilized on magnetic streptavidin beads, washed at 48°C and then eluted with 100mM NaOH to destroy the RNA baits, and subsequently neutralized with neutralization buffer (1M Tris-HCl, pH 7). The captured DNA was purified with a Qiagen Qiaquick spin column and eluted in 30 µL of elution buffer. This product was PCR amplified for 10 cycles using 5 µL of capture product, 18 µL of nuclease free water, 25 µL of Kapa HiFi Uracil + master mix, 1 µL 10uM universal NEB primer and 1µL sequencing non-indexed primer (5’ CAAGCAGAAGACGGCATACGAGAT 3’). The capture products were purified with 1X Ampure beads and eluted in 33 µL of 10mM Tris-HCl, and then DNA was quantified on the Qubit analyzer.

As per [(Enk et al. 2014)](https://paperpile.com/c/GftuPk/nM0ui), the first round of capture underwent a second hybridization, to increase the amount of mitochondrial DNA captured. Cleaned capture product from the first round of capture was concentrated on a speed vacuum until dry and then reconstituted with 3.4 µL of DI RNase free water. The capture product was then hybridized according to manufacturer’s protocol at 55°C for 48 hours, and subsequently cleaned via Streptavidin beads. Captured libraries were subsequently cleaned, and amplified an additional 15 cycles. Amplified capture libraries were cleaned via 0.8X Ampure bead clean up and delivered to the UC Davis Genome Center for sequencing on the Illumina Miseq. Sequencing varied between runs: Plate 1 and 2 = 250 Paired End, Plate 3 = 150 bp SE, Plate 4 = 100 bp SE.

*\subsubsection*{PaleoBARN, University of Oxford}*

Illumina libraries were built at the University of Oxford following [(Meyer and Kircher 2010)](https://paperpile.com/c/GftuPk/6H4Zb), with the addition of a six base-pair barcode added to the IS1\_adapter.P5 adapter. The libraries were then amplified on an Applied Biosystems StepOnePlus Real-Time PCR system to check that library building was successful, and to determine the optimum number of cycles to run during the indexing amplification PCR reaction. A six base-pair barcode was added during the indexing amplification reaction. This resulted in each library being double-barcoded with an “internal adapter” directly adjacent to the ancient DNA strand and which would form the first bases sequenced, and an “external barcode” that would be sequenced during Illumina barcode sequencing. Libraries were sequenced on an Illumina HiSeq 2500 (Single End 80bp) sequencer at the Danish National High-Throughput Sequencing Centre, and on an Illumina NextSeq 500 (Single End 80bp) at the Natural History Museum in London.

Mitochondrial captures were performed using the myBaits manual version 3.01 (August 2015), with a 55°C incubation for 24 hours. The heat elution step was skipped, and the captures were amplified using KAPA Hi-Fi polymerase following the myBaits manual instructions for 23 cycles. The PCR reaction was cleaned using Minelute columns.

### \subsection*{Sequencing and Data Preparation}

The samples were shotgun sequenced on the Illumina HighSeq2500 in pools of 40 samples per lane with paired end sequencing. The number of reads sequenced for each sample ranged from 3,000 to 27,551,904.

Adapters were trimmed and paired reads were merged with SeqPrep (<https://github.com/jstjohn/SeqPrep>). The UC Davis samples were demultiplexed and trimmed adapters using MiSeq Reporter version 2.3.32 (Illumina). The merged reads were then mapped using BWA aln, Burrows-Wheeler Aligner, to the CanFam3.1 dog reference genome [(Li 2013; Lindblad-Toh et al. 2005)](https://paperpile.com/c/GftuPk/0n4PK+EAkiM). The reads were then filtered using SAMtools with the quality filter set to 30 [(Li et al. 2009)](https://paperpile.com/c/GftuPk/htmCu) and PCR duplicates were removed with the MarkDuplicates application in PicardTools (http://broadinstitute.github.io/picard/). Reads that mapped to the mitochondrial genome were then extracted from the bam file and the read depth of the mitochondrial genome was calculated with QualiMap bamqc. A consensus sequence was created for each sample from these extracted reads using htsbox (<https://github.com/lh3/htsbox>). The consensus sequence for each of the samples were merged together into a single file and the sequences were aligned using the Mafft algorithm using the default parameters [(Katoh and Standley 2013)](https://paperpile.com/c/GftuPk/7sgmG).

Default parameters on MapDamage2.0 were used to assess the molecular damage patterns of the samples [(Jónsson et al. 2013)](https://paperpile.com/c/GftuPk/P9vRP). The expected damage patterns at the ends of fragments were seen on ancient samples (Fig. S1). Alternatively, the historical samples from within the most recent 150 years displayed much lower levels of damage which is also as would be expected for these samples (Fig. S1).

### \subsection*{Maximum Likelihood Tree Construction}

Maximum likelihood phylogenetic trees were constructed with RAxML from the aligned consensus sequences using a General Time Reversible model with a Gamma distribution [(Stamatakis 2014, 2006)](https://paperpile.com/c/GftuPk/nfe8C+mZFqc). Coyote *(Canis latrans)* was used as an outgroup for each of the phylogenetic trees, in addition to a reference panel of published mitochondrial genomes [(Thalmann et al. 2013; Pang et al. 2009; Imes et al. 2012; Frantz et al. 2016; Botigue et al. 2016; Ní Leathlobhair et al. 2018)](https://paperpile.com/c/GftuPk/Oq0dQ+FegVc+jEaD0+iMyeI+DI1p6+VwOGC). Each phylogeny was constructed with 1,000 bootstraps to ensure the robustness of each of the nodes of the tree. Separate maximum likelihood trees were constructed from aligned samples with a mean of 1x read depth, a minimum mean of 3x read depth of 80\% of positions in the mitochondrial genome, and a minimum mean of 10x read depth of 95\% of positions for a robust representation of phylogenetic relationships (Fig. S2). The output from RAxML was visualised using FigTree v1.4.3 (Rambaut: http://tree.bio.ed.ac.uk/software/figtree/).

### \subsection*{Bayesian Skyline Analysis}

Bayesian Skyline Plots (BSPs) were computed using BEAST2.4 software to infer effective population sizes. Analyses were run for subsets of the data divided by phylogenetic clade affiliation and by cultural affiliation. A minimum threshold of 10X mean read depth was set for all bayesian analyses. Skyline analyses were run to compare differences in population size across different populations of dogs. The first Bayesian skyline analysis contained a reference panel of published dogs from [(Ní Leathlobhair et al. 2018; Thalmann et al. 2013)](https://paperpile.com/c/GftuPk/VwOGC+Oq0dQ) in addition to the 76 new complete mitochondrial genomes with over 10x read depth (Fig. S3). BSPs were also run for subsets of the data based on their age to assess differences in the effective population size based on their cultural affiliation or sample age. The same Three analyses were undertaken to address the difference: 1) Inuit dogs (n=22) from across the North American Arctic, 2) Recent dogs (n=39) from 300 years before present to the current day in the North American Arctic, and 3) Recent dogs (n=28) from 300 years ago until 50 years ago. The third analysis was undertaken to separate the influence the impact of unbalanced representation of samples in the dataset in the recent populations. All samples from the North American Arctic which pass the 10x read depth threshold are exclusively from Greenland and all but two carry related haplotypes which could reflect an accurate representation of haplotype frequencies over the last five decades or alternatively the small sample size and/or sampling bias. Due to the small sample size in the Paleo-Inuit dataset (n=2), this population was not not included in the culture/age based analyses.

To run these analyses xml files were created using BEAUti, a graphical interface function of BEAST. The datasets imported into the xml file using BEAUti were alignments of the samples partitioned based on codon position, tRNA, rRNA, and control region after removing repeating regions. Each partition was assigned a different substitution model on the basis of the recommendations given by partitionfinder: coding regions (HKY), tRNA (HKY), rRNA (TN93+G), and control region (HKY). Within BEAUti individual priors were set for each of the samples based on their ages with a uniform distribution for the age of the tip. A strict molecular clock was used as recommended in previous studies [(Thalmann et al. 2013)](https://paperpile.com/c/GftuPk/Oq0dQ). 50 million MCMC (Markov Chain Monte Carlo) chains were run on the dataset with trees being samples every 5000 iterations with a 10\% burnin. Tracer v.1.6 was utilised to evaluate convergence and visualisation of the effective population size of the dataset. Trees created with BEAST were visualised using Maximum Clade Credibility tree type using TreeAnnotator v1.8.3 with a 10\% burnin. Topology agreement was found between the tree generated by RAxML and the whole dataset tree generated by BEAST.

### \subsection*{Haplotype Determination}

D-loop haplotypes were determined by extracting the 609 bp HVI portion from all sequenced mitogenomes. The haplotypes were determined by using the Canis mtDNA HVI database <http://chd.vnbiology.com/> [(Thai, Chung, and Tran 2017)](https://paperpile.com/c/GftuPk/GVPHf). Sequences that contained too many ambiguous bases were haplotyped via BLAST, and or by alignment to known haplotypes [(Savolainen et al. 2002)](https://paperpile.com/c/GftuPk/r08QL).

A portion (N =193/480) of the UCD samples were Sanger sequenced as per [(Sarah K. Brown, Darwent, and Sacks 2013)](https://paperpile.com/c/GftuPk/61pmw). Of those samples that were Sanger sequenced, only N = 35 did not have mitogenome sequences, and thus the D-loop haplotype information was used to classify the individual to a clade.

On the basis of phylogenetic subclades found in all iterations of phylogenetic trees generated in this study, four haplogroups within the A-clade were identified. For the purposes of this study these clades representing haplogroups were identified as A1a, A1b, A2a, and A2b. To confirm the topology and obtain the most robust confidence in the phylogenetic relationships the 10x read depth dataset utilised for bayesian and maximum likelihood analyses were adjusted to exclude samples basal to the clades to assess for support at these nodes. The naming of the main dog clades (A-F) follows the convention of previous studies [(Savolainen et al. 2002; Pang et al. 2009)](https://paperpile.com/c/GftuPk/r08QL+FegVc); however, the nomenclature used in this study to refer to the subclades (A1a = haplotypes A18, A20, A22, A1b = haplotype A29, A2a = haplotype A31, A2b = haplotype A3) was created for this study.

## \section*{Ancient DNA Expanded Results}

### \subsection*{A-clade Subclades and Haplotypes}

A maximum likelihood tree was constructed from all samples which had a minimum of 3x read depth of the mitochondrial genome (Fig. S4, Fig. S5). All Inuit and Palaeo-Inuit samples fall in the previously identified Dog A-clade, together with the historical dogs from Greenland, Canada, and Alaska. Three samples from Russia included in this study, a Neolithic dog from the Boisman II site (AL2989), an ethnographic sample collected from near the Anadyr River (AL3032), and a 2,400 year old individual from Pospelov (AL2992) all fall basal to the A-clade. Four subclades (A1a, A1b, A2a, A2b) were identified in our dataset within the A-clade. Haplotypes identified from partial and complete mitochondrial genomes were assigned to each of the four subclades identified.

The A1a subclade contains modern non-Arctic dogs of a global distribution, modern Greenland Sled Dogs (n=10) and historical dogs found across the Arctic from Chukotka through to East Greenland (n=12). There is no appearance of pre-contact dogs or Paleo-Inuit dogs, however, two Thule dogs from Alaska and Greenland carry A1a haplotypes. The dog (ZMK 113a/1950-P27:TRF-01-23) from the Paleo-Inuit site at Deering, Alaska, was directly radiocarbon dated to 287±24BP, or 435 to 353 cal.BP (see Deering Paleo-Inuit site description and Table S5). The date shows that this dog was deposited during the Thule era, possibly related to the contemporary Western Thule to the east of the Ipiutak site. The other Thule dog carrying the A1a haplogroup (P68/2016KMG:TRF-01-09) was excavated from a site in northeast Greenland called Dødemandsbugt. This dog was recovered from a house from the early phase of the site, dating to the 15th to 17th centuries CE. The appearance of the A1a haplotype during the Thule Inuit era in Northeast Greenland could help support hypotheses about the Thule settlement of northeastern Greenland, with the isolated location of this sample may be related to the arrival of the Thule Inuit to northern Greenland from the Thule District via Peary Land [(Johnson 1933; Sørensen and Gulløv 2012)](https://paperpile.com/c/GftuPk/Rjjhp+aRAdo).

Two further haplogroups, A1b and A2a are isolated to Siberian and American Arctic, with the exception of an imported Greenland Sledge Dog in Denmark, a historical (1600-1800 CE) dog from Iceland, and a dingo which are known to be of Asiatic origin [(Savolainen et al. 2004)](https://paperpile.com/c/GftuPk/9PizQ). Haplogroup A1b contains modern individuals, including a previously published dingo and Siberian husky, modern Greenland Sledge Dogs (n=2), as well as historical dogs from Siberia to East Greenland (n=11). Additionally, clade A1b includes Thule Inuit dogs (n=16) from sites across the American Arctic, dogs (n=5) from Birnirk Culture sites in Chukotka, and two published 1,700 year old dogs from Aachim Lighthouse, Siberia [(Lee et al. 2015)](https://paperpile.com/c/GftuPk/GLfzM). Haplogroup A2a is found in seven of the published dogs from Zhokhov site fall in the earliest branches of the clade [(Lee et al. 2015; Ní Leathlobhair et al. 2018)](https://paperpile.com/c/GftuPk/GLfzM+VwOGC). Historical dogs from Alaska, Canada, and Greenland (n=16), as well as one historical dog from Iceland, are found within clade-A2a, along with Thule Inuit dogs from Canada and Greenland (n=36), two Birnirk culture dogs from the Walakpa site in Alaska, and one Paleo-Inuit individual from the Chugachik in Alaska which is attributed to the Kachemak Culture from approximately 2,000 BP.

Finally, A2b haplotypes are found in all precontact dogs (n=68), consistent with the work by Ní Leathlobhair and colleagues, a monophyletic lineage is formed by all previously sampled precontact American dogs from the past ~9,000 years [(Ní Leathlobhair et al. 2018)](https://paperpile.com/c/GftuPk/VwOGC) (Fig. S4). However, the clade does not exclusively contain precontact dogs, with a temporal and geographic range of Arctic dogs also falls into this subclade. A2b contains historical dogs from Alaska (n=1) and Greenland (n=1), Thule dogs from Alaska and Canada (n=8), Punuk and Old Bering Sea Culture dogs from St. Lawrence Island on the coast of Alaska (n=3), Paleo-Inuit dogs from Canada and Kodiak Island, Alaska (n=10), and dogs from the Okhotsk Culture of the Kurils (n=2). The only dogs from Greenland which fall into the the A2b clade are from north western Greenland in the Thule District from a site called Iita thought to date to the late 19th century CE.

Interestingly, no haplogroup was restricted to a particular region of the North American Arctic, which is consistent with a model of rapid range expansion of sledge dogs. However, there seems to be a geographic pattern in the dataset; for example 60\% of East Greenland dogs from the Inuit period to present show haplotypes from the A1b subclade, making it the most abundant haplogroup in East Greenland. However, it is currently difficult to assess if such pattern reflects sampling bias.

High read depth phylogenetic trees were constructed using samples with at least 10x read depth of the mitochondrial genome. Maximum likelihood (ML) trees were constructed with RAxML and a bayesian Maximum Clade Credibility tree with BEAST (Fig. S6, Fig. S7, Fig. S8). As a result of this increased read depth requirement only 22 Inuit and five Paleo-Inuit samples were included in the alignment. The appearance of the four A-clade subclades is maintained in the 10x ML trees with greater support for the nodes. The novel subclade of historical dogs and Neolithic canids within a Eurasian wolf clade is also preserved in this phylogeny with more robust bootstrap values; however, the minimum threshold cutoff has reduced this to three samples (TRF.02.29, AL3007, 03.P04.H1.1024). Of the three dogs basal to A-clade in the 3x phylogeny, two met the increased 10x threshold (AL2992, AL3032), and these samples retain their positions basal to the clade.

### \subsection*{Demographic Histories of Inuit and Historical Dogs}

Effective population size of populations divided by age was inferred from BSPs generated with BEAST2.4 (Fig. S9).

When one continuous population is assumed for dogs starting in the Inuit era of North America and continuing to present day a decrease in the effective population size can be seen between 15,000 and 5,000 years ago with a drop in the effective population size. This is followed by a small increase in the mean effective population size 2,000 years ago, returning to a similar population size as seen at 15,000 years ago. Coinciding with the Thule migration around 1,000 years ago, the effective population size decreased and is estimated to have continued to decrease until present day.

Bayesian estimation of the effective population size of only Inuit dogs, excluding all historical and modern Arctic dogs, shows a steady population until approximately 5,000 years ago when the effective population size decreased gradually until plateauing around 3,000 years ago. Another, more abrupt, decrease in the effective population size occurred approximately 1,000 years ago, dropping until 500 years ago. This decline corresponds to the timing of the Thule Inuit expansion across the North American Arctic and reflects the bottleneck in the expanding population.

The demographic analysis of North American Arctic dogs from the last three centuries contrasts with that of the Inuit dogs. The historical dogs show no decrease in the effective population size before 3,000 years ago; however, the recent dog populations show a decrease 2,500 and 1,200 years ago. This decline likely represents the Thule Expansion, however the dates are not completely in sync due to the absence of Inuit dogs in the analysis to calibrate the age of the decline. Starting around 250 years ago the population underwent an additional, more rapid decline in the effective population size. When comparing the results of the two analyses (Analysis-1 of dogs from 300 years ago until present and Analysis-2 of dogs from 300 to 50 years ago) the key difference is seen in the estimated population size at time 0 or present. When the 11 modern Greenland Sledge Dogs are included in the analysis the mean effective population size of the current population decreases by 27%. Analysis-1 estimated the mean effective population size at time 0 to be 675 while Analysis-2 estimated a mean effective population size of 2,463.

When the dataset is subdivided based on cultural affiliation/age a change in the effective population size can be seen in connection with the bottleneck experienced by Inuit dogs during the Thule Expansion. Additionally, a decline in the effective population size has also occurred over the last 250 years, coinciding with increased urbanisation in the Arctic following the arrival of Europeans as well as a changing climate. While the sampling strategy of modern dogs may have underrepresented the diversity in modern Arctic dogs from the most recent 50 years, there is no doubt that there has been a marked decline in the effective population of North American Arctic dogs over the last three centuries. This decrease in population size is corroborated with census data from dogs in Greenland which have experienced a drop in numbers from 36,521 dogs in 1998 down to 14,126 dogs in 2016 (Source: Greenland Statistics).

Bayesian Skyline simulations can be informative about the general trends which have occurred over time in a population; however, as demonstrated there can be limitations to the strict interpretation of the results. For example, the census information of the current population size of Greenland Sledge Dogs shows the stark difference between the simulated effective population size and the genuine population size. This is a recognised phenomena, in which the effective population size is an underestimate of the observed population size (xx). As a result of the underestimation inherent in effective population size simulations the general trends are the noteworthy aspects of the estimates rather than the estimated population size itself.

### \subsection*{Analyses of AL2797 Nuclear Genome}

We obtained enough reads (>700,000 reads mapping to CanFam3.1) from a sample obtained from the site of Nunalleq (AL2797) to conduct some basic nuclear analysis. We used the same SNPs set and same samples as in [(Ní Leathlobhair et al. 2018)](https://paperpile.com/c/GftuPk/VwOGC)see Table S6 for a list of genomes with accessions). Using smartpca [(Patterson, Price, and Reich 2006)](https://paperpile.com/c/GftuPk/EHUqt) we performed Principal Components Analysis (PCA) projecting AL2797. This analysis indicates that AL2797 is more closely related to wolves than dogs (including Arctic dogs; Fig. S10). We then used plink v1.9 [(Purcell et al. 2007)](https://paperpile.com/c/GftuPk/yC722) to compute an Identity By State (IBS) matrix using all SNPs. This matrix was used to build a neighbour joining tree (NJ) using the R package “ape” [(E. Paradis, Claude, and Strimmer 2004)](https://paperpile.com/c/GftuPk/U9xiu). The tree captures the deep split between East Asian and Western Eurasian dogs [(Frantz et al. 2016)](https://paperpile.com/c/GftuPk/iMyeI) and demonstrate the close affinity of AL2797 with North American wolves (Fig. S10).

We then computed D-statistics of the form D (AndeanFox, AL2797, Pop3, Pop4) using ADMIXTOOLS [(Patterson et al. 2012)](https://paperpile.com/c/GftuPk/gSD4i) where Pop3 was fixed as either European dogs, Asian dogs, Arctic dogs, or CTVT and Pop4 represented possible other sample. We plotted, as box plots, the results of these combinations (Fig. S10). Positive values imply that the AL2797 share more derived alleles with the population on the y axis, while negative values imply that pre-contact dogs are closer to the other populations. The results indicate that AL2797 is most closely related to North American wolves.

## \section*{Geometric Morphometrics Methods}

Morphometric analysis of the dog crania in 3 dimensions (3D) and hemi-mandible and lower M1 in 2 dimensions (2D) was performed on specimens from Paleo-Inuit and Inuit archaeological sites as well as ethnographic materials were sampled from historical, and modern contexts (Table S3) using landmark and sliding semi-landmark based geometric morphometric approach. Modern and Pleistocene wolves from the North American Arctic were also included in the analyses to provide a baseline wild reference morphology (Fig. S13).

### \subsection*{2D hemi-mandible and lower M1}

Data was collected from 2D digital photographs taken of the labial surface of the mandible and the occlusal view of the lower M1. Photographs were taken with a reflex camera paired with a fixed 60mm micro lens following a standardised protocol where the position of the mandible was maintained as parallel to the focal plane of the camera using a bubble level and supported by plasticine if necessary. The camera was positioned directly above the specimen and also levelled using a bubble level to maintain consistency in camera positioning. Each picture contained a scale on the same plane as the element of interest. Mandible photographs were taken by CA, AH-B and AE. Photographs of the lower M1 were taken by AH-B. Coordinates of landmark and sliding semi-landmarks were digitized on photographs of the hemimandible and lower M1 using the software tpsDIG2 [(F. J. Rohlf 2004)](https://paperpile.com/c/GftuPk/7r5CD). The mandible was captured using a series of 15 landmarks while the lower M1 protocol used 2 landmarks and 49 sliding semi-landmarks along the outline of the tooth (Fig. S14). All mandibles were digitised by CA, and all lower M1s by AH-B.

### \subsection*{Building 3D models of crania using photogrammetry}

Photographs of the canid crania were taken following the semi-automated protocol outlined in the supplementary information of [(Evin et al. 2016)](https://paperpile.com/c/GftuPk/MyxQO). Crania were placed at the centre of a round turntable which also contained a scale. Photos were taken using a reflex camera paired with a fixed 60mm micro lens from three angles relative to the specimen, at approximately 40°, 15° and 0°. At each height, 36 photographs were taken at every ~10°, covering an entire 360° rotation. This process was repeated for both the dorsal and the ventral side of the specimen, so that for each specimen a total of approximately 216 photographs were taken (i.e. [~36 photos x 3 angles] x 2 sides). These images were then used to build 3D models of the canid crania using Agisoft Photoscan Professional (version 1.4.1, 2018). 3D coordinates of 30 landmarks were acquired on the models using MorphoDig (version 0 beta 5, 2018) with 30 landmarks (Fig. S14). All models were built and digitised by AE.

### \subsection*{GMM Statistical Analysis}

Sets of coordinates were superimposed independently using General Procrustes Analyses [(F. James Rohlf and Slice 1990; Goodall 1995)](https://paperpile.com/c/GftuPk/MvJjy+eF8ma). During the superimposition of the lower M1 data, sliding semi-landmarks were allowed to slide following the Procrustes distance minimization criteria [(Sampson et al. 1996)](https://paperpile.com/c/GftuPk/514TD). The analysis of GMM data was performed in RStudio using R version 3.4.3 [(R Core Team 2018)](https://paperpile.com/c/GftuPk/35fIo) with base functions, and the libraries Morpho [(Schlager 2017)](https://paperpile.com/c/GftuPk/FTCeR), Geomorph [(Adams, Collyer, and Kaliontzopoulou 2018)](https://paperpile.com/c/GftuPk/ikIdF), ape [(Emmanuel Paradis, Claude, and Strimmer 2004)](https://paperpile.com/c/GftuPk/tnmEq), and function “mevolCVP” [(Evin et al. 2013)](https://paperpile.com/c/GftuPk/2u38J).

Differences in size between the three dog groups and the modern and pleistocene wolves were tested with a Wilcoxon test for size and visualised with boxplots (Fig. 1.A). Variation in shape was visualised with Principal Component Analyses (PCA) (Fig. S15) and the differences tested with multivariate analyses of variance (MANOVA) and Canonical Variate Analyses (CVA) (Fig. S16) paired with leave-one-out cross-validation, performed with the R function “mevolCVP” [(Evin et al. 2013)](https://paperpile.com/c/GftuPk/2u38J). Cross Validation Percentages (CVP) obtained from 1000 resampled balanced designs were expressed as a mean CVP and a 90\% confidence interval [(Evin et al. 2013)](https://paperpile.com/c/GftuPk/2u38J). Because discriminant analyses are sensitive to unbalanced sample size between groups and to the number of variables used as group predictors compare to the number of specimens per group we used a resampling approach based on 100 discriminant analyses performed on balanced sample size (randomly selecting specimens in the larger group to match the size of the smaller group), this was paired with a dimensionality reduction approach selecting the N first principal components of the PCA that maximise the differences between groups [(Baylac and Friess 2005)](https://paperpile.com/c/GftuPk/S8kCT). Following the CVA, neighbour-joining networks were constructed using Mahalanobis D^2^ distances (Fig. 1.B). Differences in shape were visualized along the discriminant axes calculated by multivariate regressions [(Monteiro 1999)](https://paperpile.com/c/GftuPk/bd8Qs)(Fig. 1.C).

Allometries (the size and shape relationship) were analysed by testing for homogeneity of within-groups allometric patterns using MANCOVAs with shape as the dependent variable, the log centroid size as a covariate and group as a factor. Only the interaction term of the Mancovas was provided (table 1). Variance analysis, also called morphological disparity, was performed using the Procrustes variance [(Zelditch et al. 2004)](https://paperpile.com/c/GftuPk/tcjWm) using the morphol.disparity function of the geomorph R package [(Adams, Collyer, and Kaliontzopoulou 2018)](https://paperpile.com/c/GftuPk/ikIdF) for shape and Fligner-Killeen test for size.

The recent Greenland dog category was composed of Historical Arctic Dogs and Modern Greenland dogs. The Historical Arctic dogs came from sites across the North American Arctic while the Modern Greenland dogs correspond to modern Greenland Sledge Dog Breed specimens from Bern, Natural History Museum (see SI). Based on our relatively small sample size we found very little morphometric differences between these two groups, so they were pooled for all the analyses included in this article. Dogs of these two categories did not differ in centroid size for the crania (W=4, p= 0.64), lower first molar (W=35, p=0.60) nor the mandible (W=43, p=0.50). We did not detect differences in the cranial shape (F(5,2)=1.86, p=0.39) nor mandible shape (F(9,9)=1.39, p=0.31). We found differences in lower M1 shape (F(4,15)=3.94, p=0.02) but since the cross validation percentages between the two groups was relatively low (67\% (58.3-83.3\%) it did not prevent the pooling of the specimens.

## \section*{Stable Isotope Analysis}

### \subsection*{Stable Isotope Analysis Methods}

Previous stable isotope analysis of carbon and nitrogen was conducted on the dogs from the Thule site of Nunalleq (GDN-249) near Quinhagak, Alaska [(E. McManus-Fry et al. 2016; Forbes, Britton, and Knecht 2015; Ledger et al. 2016)](https://paperpile.com/c/GftuPk/FLte6+gIedR+qo7mc). To investigate the relationship between domestic and wild canids, nine new specimens were sampled for stable isotope analysis, including the specimen AL2729, confirmed by analysis of nuclear DNA to be a wolf (main text, SI text). Samples were taken from mandibles and were prepared at the Department of Archaeology, University of Aberdeen, where collagen extraction was performed following a standard protocol [(Britton et al. 2013; Longin 1971; T. A. Brown et al. 1988; M. J. Collins and Galley 1998)](https://paperpile.com/c/GftuPk/B31FT+XYBID+Qd2jf+14wi1). First specimens were demineralised in 0.5 M hydrochloric acid (HCl) at 4°C, which was changed at regular intervals until demineralisation was complete, usually once or twice per specimen. Demineralisation was complete for all specimens after 14 days, and the demineralised samples were then rinsed with deionised water and gelatinised for 48h at 70°C in a solution containing three drops of 0.5M HCl in deionised water to make a weakly acidic solution (pH 3). These residues were then filtered using an E-zee-filter TM (Elkay, UK), followed by ultrafiltration to isolate the >30 kDa fraction, ensuring that only the longest collagen chains were used for analysis. Finally, the purified filtrate was frozen and lyophilised, and resulting samples were weighed into tin capsules for carbon and nitrogen isotope analysis. Isotopic measurements were performed in duplicates using EA/CF-IRMS by Iso-Analytical (Cheshire, UK) with analytical precision of $\pm$0.2 \% (1-sigma). Integrity of the archaeological proteins preserved in the specimens was established using standard collagen quality control indicators, which all samples met [(Ambrose 1990; van Klinken 1999)](https://paperpile.com/c/GftuPk/NqBfu+PEDCD).

### \subsection*{Stable Isotope Analysis Results}

The results were plotted alongside the 28 previously sampled dog specimens from [(E. McManus-Fry et al. 2016)](https://paperpile.com/c/GftuPk/FLte6) (Fig. S17; Table S4). The patterns of isotopic variation indicate that the protein in the diet of the wolf specimen AL2729 was more terrestrially-based than the dogs at Nunalleq. Interpretation of this result suggests that the wolf specimen was not living in a domestic context alongside the dogs at Nunalleq which were provisioned with a diet higher in marine proteins (likely salmon). Instead, this specimen likely represents a wild wolf, possibly killed to prevent predation, or for its fur, further evidence for the use of both dogs and wolves at Arctic sites. For specimens that were suitable for both GMM and isotope analysis, a comparison of the relationship between shape and size and diet was performed, but results indicated no relationship between these morphological variables and the diet of the Nunalleq dogs.

## \section*{Archaeological Site Descriptions}

*Below is a description of the archeological sites where canid remains were sampled for this study. Further information about the specific samples can be found in Tables S1 and S3.*

### \subsection*{North American Paleo-Inuit sites}

*\subsubsection*{Agvik, Banks Island, Northwest Territories}*

*Extract ID: OKRN-1:TUR*

Agvik (OkRn-1), also referred to as the Fish Lake site, is located on the southern coast of Banks Island overlooking Amundsen Gulf [(Hodgetts and Eastaugh 2017)](https://paperpile.com/c/GftuPk/I5JXY) ; it was initially mapped and tested in 2009 [(Arnold 2010)](https://paperpile.com/c/GftuPk/ypKpG). A juvenile dog radius was sampled from faunal remains recovered from a test unit at the head of a gully, which is stored at the Prince of Wales Northern Heritage Centre in Yellowknife. Associated caribou bones were submitted for radiocarbon dating: 420$\pm$40 BP (Beta-264000) and 380$\pm$40 BP (Beta-263998). The results fit with the estimated age of the site based on dwelling types and artifacts at AD 1350‒1550 [(Hodgetts and Eastaugh 2017)](https://paperpile.com/c/GftuPk/I5JXY).

*\subsubsection*{Angyaruaq (Jungjuk) Port Site, Middle Kuskokwim}*

*Extract ID: SLT-094-09-00507*

The Angyaruaq (49-SLT-094) is located in southwestern Alaska near the village of Crooked Creek along the north bank of the Kuskokwim River, approximately 230 km northeast of Bethel. Angyaruaq is the Yup’ik placename roughly translated as “toy canoe” that figures prominently in oral accounts of Crooked Creek’s origins. The site, excavated by Northern Land Use Research, is composed of three loci with multiple features. The canid remains come from a semi-subterranean house pit, Feature 1, in Locus 1. Feature 1 dates between AD 140-320 (1810-1630 cal BP) and has an artifact assemblage that shows affinities to the Norton tradition [(Hays et al. 2012)](https://paperpile.com/c/GftuPk/H79y3).

*\subsubsection*{Cape Addington Rockshelter, Alaska}*

*Extract ID: 49-CRG-188*

Cape Addington Rockshelter (49-CRG-188) is located on Noyes Island in the outer Prince of Wales Archipelago of southeast Alaska. Dates range from ~2000 to 500 cal BP. Throughout the site’s 1500-year occupational history, the site was used seasonally. The most common vertebrates were deer, harbor seal, halibut, salmon, Pacific cod, and eagle, with fewer remains of Steller sea lion, northern fur seal, and a variety of seabirds. California mussels were also heavily used. Dog remains were limited to three specimens [(Moss 2004)](https://paperpile.com/c/GftuPk/VOZat); a premolar tooth from Trench 1, Unit 2, Level III-B (ca. AD 450-920) was tested in this study for mtDNA [(Moss 2004)](https://paperpile.com/c/GftuPk/VOZat).

*\subsubsection*{Cape Krusenstern, Kotzebue Sound, Alaska}*

*Extract ID: CK-H10-H1, CK-H10-M1, CK-H22-P1, CK-H37-M1, CK-H40-C1, CK-H40-C2 ; P96/2013KMG-C.Krusenstern-A1*

Cape Krusenstern was designated as an Archaeological District (49-NOA-002) in 1973 because it contains a historical record of human habitation of these beach ridges for more than 5,000 years. Discovery of this rich archaeological record, known locally as Sealing Point, came late in the 1958 field season for J.L. Giddings [(J. Louis Giddings and Anderson 1986)](https://paperpile.com/c/GftuPk/grz9v). Excavations were undertaken under the leadership of Giddings from 1959 to 1961, and then after his death in 1964, Anderson returned to continue excavation in 1965 on the Ipiutak beach ridges [(J. Louis Giddings and Anderson 1986)](https://paperpile.com/c/GftuPk/grz9v). All artifacts and faunal remains, with the exception of a portion of the Old Whaling locality are housed at the Haffenreffer Museum of Anthropology at Brown University.

A total of nine Ipiutak dwellings were excavated across four beach ridges at Cape Krusenstern between 1959 and 1965 and were the focus of Doug Anderson’s PhD dissertation [(J. Louis Giddings and Anderson 1986)](https://paperpile.com/c/GftuPk/grz9v). C. Darwent reanalyzed the faunal remains from three of the Ipiutak houses—a sample of one house from each of three house clusters: House 10, House 37, and House 40. The houses were generally rectangular with driftwood roof posts and sod and wood walls. The mandible of a juvenile dog and humerus of an adult was sampled for DNA from House 10, which dates to AD 690–950; an adult mandible was selected from House 37, which dates to AD 390–780; and two crania from House 40, which dates to AD 390–780.

Five semi-subterranean, lobed dwellings and five shallow, tent-like dwellings were excavated at the Old Whaling locality between 1959 and 1960 [(J. Louis Giddings and Anderson 1986)](https://paperpile.com/c/GftuPk/grz9v). Radiocarbon dates from recent excavations at Old Whaling indicate the site was likely inhabited between 1150 and 850 BC [(J. Darwent and Darwent 2005)](https://paperpile.com/c/GftuPk/NejOP). Analysis of the faunal assemblage from Old Whaling stored at the Haffenreffer Museum [(Christyann M. Darwent 2006)](https://paperpile.com/c/GftuPk/kJd1S)(Darwent 2006) revealed two *Canis* remains. A phalanx was selected by C. Darwent for DNA testing in 2012. The following year S. Brown traveled to the Natural History Museum in Copenhagen to find dog samples from Greenland; to our surprise she discovered approximately 120 bones from Old Whaling. In 1962–63, Giddings spent a sabbatical year at the National Museum of Denmark, and it appears that faunal material from excavations at Cape Krusenstern were brought with Giddings to Copenhagen for identification by zoologist Ulrik Møhl and then remained behind when he returned to Brown University. An astragalus was selected from 12 small *Canis* fragments in a bag labeled House 22 (P96/2013KMG-C.Krusenstern-A1). The Haffenreffer Museum was not aware of their existence until this project began.

*\subsubsection*{Choris Peninsula, Kotzebue Sound, Alaska}*

*Extract ID: CP-H2-H1(CP2-1), CP-H2-H2(CP2-2), CP-H2-H3(CP2-3), CP-H2-H4(CP2-4), CP-H3-F1 (CP3-1), CP-H3-F2 (CP3-2)*

Choris Peninsula is a finger-like projection extending into Eschscholtz Bay on the eastern shore of Kotzebue Sound. The Choris site (49-SLK-007) was identified in 1956, and excavated in 1957 under the direction of J.L. Giddings [(J. L. Giddings 1957; J. Louis Giddings and Anderson 1986)](https://paperpile.com/c/GftuPk/BxIqM+grz9v). It is considered the type site for the Choris culture, with its most defining characteristics being the remains of three large ovoid (communal?) dwellings and the presence of pottery [(J. L. Giddings 1957; J. Louis Giddings and Anderson 1986)](https://paperpile.com/c/GftuPk/BxIqM+grz9v). Five AMS radiocarbon dates were run on caribou bone from Houses 1–3, and Area 3 of the Choris site and cluster between 750 and 400 BC [(Christyann M. Darwent and Darwent 2016)](https://paperpile.com/c/GftuPk/U0Pyf). Faunal remains from this site were reanalyzed by C. Darwent [(Christyann M. Darwent and Darwent 2016)](https://paperpile.com/c/GftuPk/U0Pyf), and from this analysis four *Canis* left humerii were selected from House 2 and two right femora from House 3 were selected for DNA testing.

*\subsubsection*{Chugachik Island, Upper Kachemak Bay, Cook Inlet, Alaska}*

*Extract ID: SEL-033-0057, SEL-033-1108, SEL-033-1219, SEL-033-1505, SEL-033-2250, SEL-033-2269, SEL-033-2519, SEL-033-2520, SEL-033-2907*

The Chugachik Island Site (49-SEL-033) is located on the southern end of Chugachik Island in the Kachemak Bay State Wilderness Park, in Upper Kachemak Bay, Cook Inlet in southcentral Alaska. Karen Workman led an excavation in 1974 in association with the Alaska State Division of Parks and the Youth Conservation Corps [(K. W. Workman 1975, 1978)](https://paperpile.com/c/GftuPk/xTWq8+lv6U3). Further excavations were conducted during University of Alaska Anchorage (UAA) field schools led by William Workman and John Lobdell in 1977 and 1981[(K. W. Workman 1978; Lobdell 1980; W. B. Workman, E., and Workman 1980)](https://paperpile.com/c/GftuPk/lv6U3+mqG2U+dTE2O). Additional excavations were led by UAA-affiliate Peter Zollars in 1982 and 1984 [(Zollars 1982; W. B. Workman and Zollars. 2002)](https://paperpile.com/c/GftuPk/3GmWB+oxhZ7).

The Chugachik Island site was found to consist of an extensive and complex stratified shell midden, with the main component dating to the Kachemak Tradition. Samples of charcoal and birchbark basketry collected in 1974 yielded dates of 1705$\pm$65 radiocarbon years and 2310$\pm$65 radiocarbon years, respectively . Five samples collected in 1977 and 1981 yielding dates ranging from 1475$\pm$70 radiocarbon years to 2740$\pm$75 radiocarbon years [(W. B. Workman, E., and Workman 1980; W. B. Workman 1998)](https://paperpile.com/c/GftuPk/dTE2O+Gazln). The basal component was found to date to the Arctic Small Tool Tradition

During the 1974 excavation, a canid burial was recovered from a depth of 160 cmbs and additional individual candid skeletal elements were recovered from elsewhere in the site [(K. W. Workman 1975)](https://paperpile.com/c/GftuPk/xTWq8). During the 1977 excavation, 126 canid skeletal elements, including another burial, were recovered ([(Lobdell 1980)](https://paperpile.com/c/GftuPk/mqG2U):129) determined that “none of the recovered canid limb bones were long or large enough to be other than domestic dog of a stature much smaller than wolf.” Additional canid remains were recovered during later excavations; all but one of the samples cited in this paper were recovered in 1981.

A dog tooth from SEL-033-057, Level 26 was submitted by C. Darwent for radiocarbon dating by Arizona AMS (AA10285: 2723 $\pm$ 51 BP, d13C: -12.3‰), along with a dog rib from level 12 (AA102986: 2274 $\pm$ 49, d13C -13.3‰). Given the clear marine signature of the carbon isotope values for these specimens, a marine calibration was used to compensate for the reservoir effect using Calib 7.1 [(Stuiver, Reimer, and Reimer 2019)](https://paperpile.com/c/GftuPk/4QrBn). Thus, these dog remains date to between ca. 700 BC and AD 200, which is consistent with the cultural affiliation of Kachemak.

*\subsubsection*{Coffman Cove}*

*Extract ID: 49-PET-067*

The Coffman Cove site has undergone significant damage over the years, and much of the site has been destroyed. Excavations in 2006 aimed to mitigate the adverse effects of transfer of the site from State to City ownership [(Moss et al. 2016)](https://paperpile.com/c/GftuPk/3A67b). The site is located on the northeast side of Prince of Wales Island in southeast Alaska. It was occupied between 5500 and 1000 cal BP. The most abundant fish were salmon, Pacific cod, sculpin, rockfish and herring. Harbor seal and deer were the most common mammals, and scoters, the most common bird. Dog remains were limited to 59 specimens. One tooth sample was tested in this study for mtDNA.

*\subsubsection*{Coffman Cove Ferry Terminal}*

*Extract ID: 49-PET-556*

The Coffman Cove Ferry Terminal site is located on the northeast side of Prince of Wales Island in southeast Alaska [(Reger et al. 2017)](https://paperpile.com/c/GftuPk/oA8rT). It is not as extensive as 49-PET-067, but it too has undergone damage due to construction (of a ferry terminal). It is located just 600 m away from 49-PET-067. 49-PET-556 was occupied between 3000-2000 cal BP. Although this site was occupied at the same time as one of the components at 49-PET-067, the faunal assemblages differ, with a greater emphasis on Pacific cod at the Ferry Terminal site. A total of 19 dog specimens were found at the site; a single tooth was tested for mtDNA.

*\subsubsection*{Cove Site, Knud Peninsula, Ellesmere Island, Nunavut}*

*Extract ID: SGFM-5*

Sites in the Bache Peninsula region were investigated by members of the Arctic Institute’s Ellesmere Island Research Project under the direction of Peter Schledermann between 1977 and 1982 [(Schledermann 1990)](https://paperpile.com/c/GftuPk/Bm9uR). The Cove site (SgFm-5) is located on the northeast end of Knud Peninsula, Ellesmere Island; the main structure at this site is a Late Dorset (AD 800–1100) communal longhouse defined by a “wall” of boulders and stones [(Schledermann 1990)](https://paperpile.com/c/GftuPk/Bm9uR). Nearly the entire 14.5 x 5.5 m structure was excavated over four field seasons. Faunal remains from the dwellings’ interior were dominated by birds, but they also included a mandibular carnassial tooth of an adult *Canis*, which was sampled for mtDNA at the Canadian Museum of History.

*\subsubsection*{Deering, Seward Peninsula, Kotzebue Sound, Alaska}*

*Extract ID: TRF-01-23(ZMK 113a/1950-P27), TRF-01-24(ZMK 113a/1950-P28), TRF-01-27(ZMK 113a/1950-P31), P97/2013KMG-Deering-H1(ZMK 113a/1950-P31), P98/2013KMG-Deering-C2(ZMK 113a/1950-P27), P99/2013KMG-Deering-C3(ZMK 113a/1950-P28)*

Deering is a northwestern Native Alaskan community situated on a spit within Kotzebue Sound on the northern coast of the Seward Peninsula. Several archaeological sites are located along the Deering spit that make up the Deering Archaeological District (Alaska Heritage Resources Survey number KTZ-000169) [(Sloan 2014; Peter M. Bowers and Williams 2009)](https://paperpile.com/c/GftuPk/720EY+8LcB2). One such site, KTZ-00023, in the district contains an Ipiutak *qalegi* or *qargi,* ceremonial house, discovered and excavated by Helge Larsen in 1949 [(VanStone and Lucier 1974)](https://paperpile.com/c/GftuPk/Gvk1t).

This unusually large ceremonial structure, or ‘men’s house’, was built with a foundation of non-locally sourced spruce [(Larsen 2001)](https://paperpile.com/c/GftuPk/q9sp). The site, dated to between AD 700 and 900, is situated just to the east of later Western Thule houses, which are dated to between AD 1030 and 1640 [(Sloan 2014; Larsen 2001)](https://paperpile.com/c/GftuPk/720EY+q9sp). The antechamber of the qalegi contained preserved organic materials such as wood shavings, animal bones, and dog faeces [(Larsen 2001)](https://paperpile.com/c/GftuPk/q9sp). Additionally, remains of sledges were preserved at the site which indicate that the sledge style used by the Ipiutak people at Deering was that of a built-up sledge which had previously not been seen in North America and was more reminiscent of similarly aged sledges in Sweden, Finland, and western Siberia [(Larsen 2001)](https://paperpile.com/c/GftuPk/q9sp). Larsen argues that the combination of dog remains and faeces as well as sledge materials presents evidence for the possibility of the use of the dogs as traction to pull the sledges [(Larsen 2001)](https://paperpile.com/c/GftuPk/q9sp). The left petrous bone was sampled from three individuals for the genetic portion of this study derived from the third and fourth layers of the site, these layers have been radiocarbon dated to approximately 1250 years before present [(Larsen 2001)](https://paperpile.com/c/GftuPk/q9sp). Two of the specimens (TRF-01-23:ZMK 113a/1950-P27, TRF-01-24:ZMK 113a/1950-P28) were directly radiocarbon dated for this study at the University of Oxford Radiocarbon Accelerator Unit. Both specimens returned dates which postdate the Ipiutak occupation and fall within the Thule era, see Table S5. Direct radiocarbon dating of sample TRF-01-24:ZMK 113a/1950-P28 had greater uncertainty as a result of the reservoir corrections required. TRF-01-24:ZMK 113a/1950-P28 showed a marine reservoir offset, therefore for calibration a mixed curve was used (INTCAL13 and MARINE13, as recommended by the University of Oxford Radiocarbon Accelerator Unit, after [(Reimer et al. 2013)](https://paperpile.com/c/GftuPk/nX6QW). The marine proportion was estimated using interpolation, two endpoints of -21 per mille and -12.5 per mille, with a result of 74\% marine component with 10\% uncertainty.

*\subsubsection*{Icebreaker Beach Site, Truelove Lowlands, Devon Island, Nunavut}*

*Extract ID: QKHN-13:1654*

The Icebreaker Beach site (QkHn-13) is an early Predorset occupation located in the Truelove Lowland region of northern Devon Island. Radiocarbon dating of unburned caribou bone dated to approximately 2500–1900 BC [(Helmer 1991)](https://paperpile.com/c/GftuPk/rWm09). A large canid “quite possibly dog,” was reported from this site. A distal humerus of an adult *Canis* was pulled from the Feature 15 (indeterminate feature type) faunal remains at the Canadian Museum of History and sampled for mtDNA.

*\subsubsection*{Kaleruserk, Igloolik, Nunavut}*

*Extract ID: TRF-01-22(ZMK-123c/1955), P80/2013KMG-Igloolik-F1, P87/2013KMG-Igloolik-F2*

Igloolik is an island just north of Canada’s Melville Peninsula, within the Foxe Basin, and is associated with a Paleo-Inuit settlement [(Maribeth Suzanne Murray 2005)](https://paperpile.com/c/GftuPk/R22x6). The site was originally explored in the 1950s by Jørgen Meldgaard, and dog remains were recovered from the Káleruserk locality, also known as Perry Hill. These remains are assumed to be pre-Dorset because they are associated with deposited dated to the early fourth millennium B.P. [(Morey and Aaris-Sørensen 2002; Jørgen Meldgaard 1960; Maribeth Suzanne Murray 2005)](https://paperpile.com/c/GftuPk/9kaOs+Bl7oE+R22x6). Up to as many as 29 intact and disturbed tent rings were identified on the site [(Maribeth Suzanne Murray 2005)](https://paperpile.com/c/GftuPk/R22x6). A crania from Igloolik was analysed for mtDNA analysis for this study, and two femora were also sampled for mtDNA.

*\subsubsection*{Kugzruk Island, Lopp Lagoon, Seward Peninsula, Alaska}*

*Extract ID: KZ-7005-1*

Kugzruk Island (49-TEL-149) is located northeast of Cape Prince of Wales on Seward Peninsula. Two sites were discovered by J. L. Giddings in the barrier beach area to the front of Lopp Lagoon, Agualak Island and Kugzruk Island, in 1959. On Kugzruk Island, they located two Norton-period house pits and a possible third that had eroded into the adjacent pond; he named this cluster Site 1: Kugzruk Pond, Houses 1 and 2 [(J. Louis Giddings and Anderson 1986)](https://paperpile.com/c/GftuPk/grz9v). Wood from the pond excavations produced a date of 2306 ± 38 BP [(J. Louis Giddings and Anderson 1986)](https://paperpile.com/c/GftuPk/grz9v), which calibrates to 400–200 BC and fits with the presumed Norton age based on house shape and artifacts. Fauna is described as coming from the “bottom of a small pond adjacent to the site and partially excavated from the mound itself” according to notes with the collection at the Haffenreffer Museum of Anthropology, Brown University. The bones, which included a phalanx, three metatarsals, five metacarpals, and a distal humerus were all stamped with the number 7005. A dog maxilla from this collection was sampled for DNA.

*\subsubsection*{Lagoon, Banks Island, Northwest Territories}*

*Extract ID: OJRL-3:534*

The Banks Island Archaeological Research Project, directed by Charles Arnold, focused on the excavation of the Lagoon site (OjRl-3) in 1976 and 1977 [(Arnold 1980)](https://paperpile.com/c/GftuPk/fQIXT)(Arnold 1980). Area A at this site included a buried cultural component with dwelling remains and associated midden areas with excellent organic preservation. In addition to faunal remains and osseous artifacts, a wooden fragment of a bowl, a skin kamik and bag, a leather thong, knotted muskox hair, and knotted baleen. Subsistence at this site was focused on geese, ptarmigan, ringed seal, and to a lesser extent muskox and arctic fox.

This site, however, is best known for the remains of three *Canis* vertebrae, 12^th^ thoracic to 1^st^ lumbar; “the dorsal surface of the spinous process of each vertebra is flattened and lipped” ([(Arnold 1979)](https://paperpile.com/c/GftuPk/iIj4y):264), and suggests the animal may have carried a pack. A small fragment of bone was collected at the Prince of Wales Northern Heritage Center from the elements for mtDNA sampling (OJRL-3:534; 982.50.534) Radiocarbon dates on three muskoxen scapulae from Area A yielded dates indicating this early Dorset site was likely occupied from 825–200 BC.

*\subsubsection*{Mill Island, Baffin Island, Nunavut}*

*Sample ID: HMCZ\_47059*

Mill Island is located in Hudson Strait off the southwestern coast of Baffin Island. The Dorset site excavated there in the 1950s consisted of two semi-subterranean houses and their associated midden [(Park 1993)](https://paperpile.com/c/GftuPk/0Ngo5). While the architecture at the site is reminiscent of Thule type, all of the recovered artifacts were identified as Dorset, with the archaeologists concluding there was no contact between Dorset and Thule peoples at this site, nor any evidence of long term re-occupation of the structures, although it is likely that the Thule remodelled the house at some point. A single lower M1 from this site was included in this analysis.

*\subsubsection*{Nanook, Kimmirut (Lake Harbour), Baffin Island, Nunavut}*

*Extract ID: KDDQ-9*

The Nanook site (KkDq-9) is located near Kimmirut (Lake Harbour) in the Tanfield Valley of southern Baffin Island, Nunavut, and was excavated under the direction of Moreau Maxwell in 1962 [(Maxwell 1973)](https://paperpile.com/c/GftuPk/zkj10). The buried remains of an amorphous stone and sod structure with a slight central depression (approximately 2 x 3.5 m) were unearthed in a grassy gully described as Nanook, Component 2 [(Maxwell 1973)](https://paperpile.com/c/GftuPk/zkj10). Artifacts are characteristic of eastern Arctic early to middle Dorset sites, and calibrated radiocarbon dates suggest the site was occupied from approximately 420 BC to AD 120 [(Maxwell 1973)](https://paperpile.com/c/GftuPk/zkj10).

This site unearthed the first Dorset-age dog remains; measurements of the partial cranium have been suggested to fall within that of a large Inuit dog [(Cleland and Haag 1973)](https://paperpile.com/c/GftuPk/K9Vlj). The prominent sagittal crest is indicative of a male. Cleland appears to have refit and glued the fragmentary cranial pieces to undertake his analysis. Dark discoloration of the bone was from soil staining, as there were no signs of burning. A sample of the P4 root was taken at the Canadian Museum of History for mtDNA.

*\subsubsection*{Nipisat I, Sisimiut District}*

*Extract ID: TRF-01-14(ZMK 136/1989), P86/2013KMG-Nipisat-H1, Pxx/2013KMG-Nipisat-H2*

The Saqqaq site, Nipisat I, is located in the Sisimiut District of western Greenland [(Morey and Aaris-Sørensen 2002; Gotfredsen and Møbjerg 2010)](https://paperpile.com/c/GftuPk/9kaOs+3lfd4). Nipisat I was continuously occupied between 4000 and 2500 BP, the Saqqaq site with the longest and well dated occupation [(Morey and Aaris-Sørensen 2002)](https://paperpile.com/c/GftuPk/9kaOs). Excavations at Nipisat I yielded 17 bones and teeth belonging to Saqqaq dogs [(Morey and Aaris-Sørensen 2002; Gotfredsen and Møbjerg 2010)](https://paperpile.com/c/GftuPk/9kaOs+3lfd4). The dog remains are all derived from the third phase of the site, during the most recent occupation, from 3000 to 2500 BP [(Morey and Aaris-Sørensen 2002)](https://paperpile.com/c/GftuPk/9kaOs). The samples taken for this study were drilled from a canine tooth, and two humeri.

*\subsubsection*{North Point, Gulf of Alaska}*

*Extract ID: SUM-025-74*

The North Point site (49-SUM-025) is located in a small cove on the south shore of Port Houghton, approximately 35 km northeast of Kupreanof Island and 25 kn east of Admiralty Island, southeast Alaska [(P. M. Bowers and Moss 2001; P. M. Bowers et al. 2001)](https://paperpile.com/c/GftuPk/rsaoh+3nReU). The site is also known as the “North Point Wet site” due to it being situated in the intertidal zone, and partial inundation of water creating an excellent medium for organic preservation. The site was investigated in 1994, 1995 and 2005 by Northern Land Use Research Inc. and US Forest Service archaeologists. The occupations at the North Point site dates between 2200-2800 BP (300 to 950 BC).

*\subsubsection*{Nunguvik, Navy Board Inlet, Baffin Island, Nunavut}*

*Extract ID: PGHB-1:1535*

The Nunguvik site (PgHb-1) is located on the west shore of Navy Board Inlet on northeastern Baffin Island. This is an extensive site, with more than 80 houses, that spans early and late Dorset and early and late Thule. Father Guy Mary-Rousselière excavated the remains of several house structures from both Dorset and Thule in the early 1970s [(Mary-Rouselière 1976, 1979)](https://paperpile.com/c/GftuPk/prR3D+kTxcj). The cranium of an old, female dog was recovered from House 71, a rectangular Dorset house that dates to AD 130‒580. Mary- Rousselière [(Mary-Rouselière 1976)](https://paperpile.com/c/GftuPk/prR3D) reports that 700 bones were found during the 1971 season, primarily caribou and bird, but also seal and other mammals, including dog; he comments that the dog is “perhaps intrusive,” suggesting that it may, in fact, be from later Thule occupation of the site. The cranium was missing its teeth post-mortem; however, she appeared to have experienced loss of at least one tooth pre-mortem with alveolar resorption, and she had clearly suffered a blow to the frontal. Because the sample failed to yield DNA, it was not submitted for direct radiocarbon dating.

*\subsubsection*{Point Hope, North Slope, Alaska}*

*Sample ID: NMNH\_241525*

Point Hope is the type site for the Ipiutak culture, located on the western edge of Alaska, above the Arctic circle on the Chukchi sea. The site has been dated to between 500 - 900 AD, and consists of both seasonally occupied dwellings and a large cemetery [(Hill 2018)](https://paperpile.com/c/GftuPk/UfdM7). While much of the excavations have focused on the human remains from the cemetery, a significant number of faunal remains were also collected, including the remains of a single dog used in this analysis, which includes a crania, a mandible and a lower M1.

*\subsubsection*{Port Joli , Nova Scotia}*

*Extract ID: ALDF-30A-1568, ALDF-30A-911, ALDF-30A-912*

These dog samples, which serve as a North American Arctic outgroup, are derived from the Area A shell midden at AlDf-30, a small Middle Maritime Woodland Period site in Port Joli, Nova Scotia [(M. W. Betts, Burchell, and Schöne 2017)](https://paperpile.com/c/GftuPk/rwmHX). Dating to ca. AD 575-660, the site is located approximately 250 meters from the high tide mark, on a knoll in the middle of a small fen. The appendicular bones of the dog were found articulated, but no evidence of an intentional interment was present (the mandible was found in an adjacent level). The kitchen midden abutted multiple concatenated living surfaces (likely wigwam floors), and a sweat house structure. A phalanx and two premolars were sampled for mtDNA.

*\subsubsection*{Port Refuge, Cape Grinnell, Devon Island, Nunavut}*

*Extract ID: RBJU-1:1107, RBJU-1:COLD*

Archaeological research was undertaken by Robert McGhee, between 1972 and 1977, at the Port Refuge site (RbJu-1) situated along Port Refuge Bay on the south coast of Grinnell Peninsula, Devon Island [(McGhee 1979)](https://paperpile.com/c/GftuPk/md6Mc). One component of the site, the Cold component, is located on a beach ridge between 22 and 25 masl approximately 600 m inland from the northern shore of Port Refuge Bay; faunal remains were excavated and collected from 12 features, which included a Canis humerus midshaft (RBJU-1:COLD) sampled for mtDNA. At the Upper Beaches component, located at the southwestern entrance to Port Refuge Bay, faunal material was recovered from three features, and two putative dog remains were identified. Both were examined at the Canadian Museum of History and were identified as a juvenile maxilla with P3 (P4 erupting), and a juvenile mandible with canine and P3 (P4 erupting). As these likely came from a single individual only one (RBJU-1:1107) was sampled for mtDNA. All materials from this site are housed at the Canadian Museum of History.

*\subsubsection*{Qajaa, Disko Bay}*

*Extract ID: TRF-01-64(ZMK350/1982-P79), ZMK350/1982-P78, P81/2013KMG-Qaja-H1, P83/2013KMG-Qaja-T1*

Qajaa is an archaeological site from the Saqqaq culture located on the eastern side of Disko Bay in western Greenland [(Morey and Aaris-Sørensen 2002)](https://paperpile.com/c/GftuPk/9kaOs). Due to the location of the site along the shore much of the site has been eroded by the sea [(Møhl 1986)](https://paperpile.com/c/GftuPk/LDMJS). The site is estimated to have accumulated over approximately 900 years, between 3600 and 2700 BP [(J. Meldgaard 1983; Morey and Aaris-Sørensen 2002)](https://paperpile.com/c/GftuPk/jObq7+9kaOs). Excavations at Qajaa uncovered 21 dog bones in the earliest phases of the site’s habitation [(Morey and Aaris-Sørensen 2002; Møhl 1986)](https://paperpile.com/c/GftuPk/9kaOs+LDMJS).

*\subsubsection*{Qeqertasussuk, Disko Bay}*

*Extract ID: TRF-01-17(ZMK 70/1983), P82/2013KMG-QT-H1, P84/2013KMG-QT-T1, P85/2013KMG-QT-H2*

Qeqertasussuk lies in the south extreme of Disko Bay in western Greenland and like Qajaa is an archaeological site attributed to the Saqqaq culture [(Grønnow 1994, 2017)](https://paperpile.com/c/GftuPk/ISyIc+m8k3f). The Saqqaq occupation of Qeqertasussuk occurred between 3900 and 3100 BP [(Morey and Aaris-Sørensen 2002; Grønnow 1994)](https://paperpile.com/c/GftuPk/9kaOs+ISyIc). 41 dog bones were excavated from the site from across the 800 years of site usage [(M. Meldgaard 2004)](https://paperpile.com/c/GftuPk/m8PEw). Among the dog remains there were several acticulated dog limbs, some of which display cut marks on the bones as a result of processing by humans [(M. Meldgaard 2004)](https://paperpile.com/c/GftuPk/m8PEw). However, despite the generally excellent organic preservation no sledge related artefacts were discovered at the site, indicating their rare or complete absence at Qeqertasussuk, in line with all other Saqqaq sites [(Morey and Aaris-Sørensen 2002; M. Meldgaard 2004)](https://paperpile.com/c/GftuPk/9kaOs+m8PEw). The samples used in this study were taken from three dog humeri and one tibia.

*\subsubsection*{Rastoden, Bache Peninsula, Ellesmere Island, Nunavut}*

*Extract ID: SFFL-10*

Sites in the Bache Peninsula region were investigated by members of the Arctic Institute’s Ellesmere Island Research Project under the direction of Peter Schledermann between 1977 and 1982. The Rastoden site is located on the southeastern tip of Thorvald Peninsula, south of Bache Peninsula, on a beach ridge 26 masl [(Schledermann 1990)](https://paperpile.com/c/GftuPk/Bm9uR). Bone from this late Predorset site (1200–1300 BC) was surface collected from Camp Area B, which included several circular tent-ring features and midden areas. Because excavation was not undertaken, the faunal assemblage is small. A maxillary P4 from Area B-4 was sampled for mtDNA at the Canadian Museum of History.

*\subsubsection*{Sermermiut, Ilulissat}*

*Sample ID: HMCZ\_10539*

The Sermermiut Valley is located in the World Heritage Site of Ilulissat Icefjord 2 kilometres south of the centre of the Disko Bay town of Ilulissat, Greenland. Excavations and survey since the 1950s have uncovered houses and middens from Saqqaq, Dorset and Thule periods in this region [(Therkel Mathiassen 1958)](https://paperpile.com/c/GftuPk/UBnPl). Due to preservation conditions for organic materials on the site, bone and wood from the Saqqaq and Dorset layers did not survive [(Therkel Mathiassen 1958)](https://paperpile.com/c/GftuPk/UBnPl). A lower M1 from a Thule era specimen was analysed in this study. This specimen is described by G. Allen [(Allen 1920)](https://paperpile.com/c/GftuPk/0fGFw) as the type specimen used for his analysis of an ‘Eskimo Dog’, which was “exhumed by Dr. AI. P. Porsild from an old village site at Sermermiut”, and is now held at Harvard’s Museum of Comparative Zoology.

*\subsubsection*{Tikilik, Igloolik Island, Nunavut}*

*Extract ID: NIHF-4:249C, NIHF-4:3422C, NIHF-4:4BSP, NIHF-4:9BSM*

Tikilik (NiHf-4) is a Late Dorset site located on a peninsula jutting out along the southwestern coast of Igloolik Island. The site was excavated by Susan Rowley in 1993–94, and faunal remains from the Paleo-Inuit components were identified by Maribeth Murray. The date of this site was estimated between 1200 and 800 BP (AD 750–1150) based on beach-ride elevation, Dorset parallel harpoon heads, and typical Late Dorset-style artwork [(Maribeth S. Murray 1996)](https://paperpile.com/c/GftuPk/g3MH1). However, there are Thule components to this site according to notes associated with the original excavation bags and unpublished documents for this site at the Prince of Wales Northern Heritage Centre in Yellowknife—discussed below. Bone recovered from the lower black soil layer is Late Dorset, and bone from the upper peat layer is associated with later Thule occupation. A cranial fragment (Unit -24N/9E; NIHF-4:249C), canine tooth (Feature 22, Unit -34N/-22E; NIHF-4:3422C), pelvis fragment (Feature 4, Unit 1N/-5E; NIHF-4:4BSP), and mandibular fragment (Feature 9, Unit 56N/-7E; NIHF-4:9BSM) were collected from the Prince of Wales warehouse storage facility for mtDNA sampling.

A portion of the dog mandible recovered from the lower black soil layer of Feature 9 (NIHF-4:9BSM) was submitted by C. Darwent for radiocarbon dating (Beta-349748: 960 $\pm$ 30 BP, d13C -17.2‰, d15N -6.9‰). The calibrated age for this specimen is AD 1020–1160, which fits with the Late Dorset designation based on stratigraphy and artifact styles.

*\subsubsection*{Uyak, Kodiak Island, Alaska}*

*Sample ID: HMCZ\_38342, NMNH\_257528, NMNH\_257533, NMNH\_257534, NMNH\_257535, NMNH\_257537, NMNH\_257540, NMNH\_257542, NMNH\_259180, NMNH\_259181, NMNH\_259182\_1, NMNH\_259182\_2, NMNH\_259182\_3, NMNH\_259182\_5, NMNH\_259182\_6, NMNH\_260310, NMNH\_260311, NMNH\_26033, NMNH\_260318, NMNH\_260320, NMNH\_260321, NMNH\_Uyak\_C21753\_3, NMNH\_Uyak\_C25, NMNH\_Uyak\_C28, NMNH\_Uyak\_C9, NMNH-256720-1, NMNH-258782-2, NMNH-258782-3, NMNH-258782-5, NMNH-258782-6, NMNH-Uyak-C018, NMNH-Uyak-M006, NMNH-Uyak-M008, NMNH-Uyak-M012, NMNH-Uyak-M014, NMNH-Uyak-M015, NMNH-Uyak-M016, NMNH-Uyak-M018, NMNH-Uyak-M019, NMNH-Uyak-M021, NMNH-Uyak-M023, NMNH-Uyak-M026, NMNH-Uyak-M030, NMNH-Uyak-M051, NMNH-Uyak-M054, NMNH-Uyak-M055, NMNH-Uyak-M056, NMNH-Uyak-M057, NMNH-Uyak-M062, NMNH-Uyak-M071, NMNH-Uyak-M073, NMNH-Uyak-M075, NMNH-Uyak-M085, NMNH-Uyak-M086, NMNH-Uyak-M087, NMNH-Uyak-M088, NMNH-Uyak-M090, NMNH-Uyak-M091, NMNH-Uyak-M092, NMNH-Uyak-M095, NMNH-Uyak-M102, NMNH-Uyak-M108, NMNH-Uyak-M109, NMNH-Uyak-M111, NMNH-Uyak-M117, NMNH-Uyak-M118, NMNH-Uyak-M121, NMNH-Uyak-M123, NMNH-Uyak-M124, NMNH-Uyak-M127, NMNH-Uyak-M130, NMNH-Uyak-M131, NMNH-Uyak-M135, NMNH-Uyak-M139, NMNH-Uyak-M140, NMNH-Uyak-M143, NMNH-Uyak-M145, NMNH-Uyak-M147, NMNH-Uyak-M156, NMNH-Uyak-M159, NMNH-Uyak-M161, NMNH-Uyak-M162, NMNH-Uyak-M166, NMNH-Uyak-M168, NMNH-Uyak-M169, NMNH-Uyak-M171, NMNH-Uyak-M177, NMNH-Uyak-M178, NMNH-Uyak-M200, NMNH-Uyak-M201, NMNH-Uyak-M202, NMNH-Uyak-M203, NMNH-Uyak-M204, NMNH-Uyak-M206, NMNH-Uyak-M207, NMNH-Uyak-M208, NMNH-Uyak-M209, NMNH-Uyak-M211, NMNH-Uyak-M212, NMNH-Uyak-M213, NMNH-Uyak-M214, NMNH-Uyak-M215, NMNH-Uyak-M216, NMNH-Uyak-M217, NMNH-Uyak-M219, NMNH-Uyak-M22, NMNH-Uyak-M220, NMNH-Uyak-M221, NMNH-Uyak-M222, NMNH-Uyak-M223, NMNH-Uyak-M224, NMNH-Uyak-M225, NMNH-Uyak-M226, NMNH-Uyak-M227, NMNH-Uyak-M228, NMNH-Uyak-M229, NMNH-Uyak-M230, NMNH257526, NMNH257536, NMNH257538, NMNH257539, NMNH257541, NMNH260312*

*Extract ID: AL3198, 560028-01, 560028-02, 560028-03, 560028-04, 560028-05, 560028-06, 560028-11, 560028-22, 560028-28, 560028-07, 560028-08, 560028-09, 560028-10, 560028-12, 560028-13, 560028-14, 560028-15, 560028-16, 560028-17, 560028-18, 560028-19, 560028-20, 560028-21, 560028-23, 560028-24, 560028-25, 560028-26, 560028-29, 560028-30, 560028-27*

The Uyak site (49-KOD-145) is a substantial prehistoric midden at least three meters deep, covering hundreds of acres and located on the western side of Kodiak Island, Alaska. The site was excavated by physical anthropologist Aleš Hrdlička from 1933-1936 for the United States National Museum (now the National Museum of Natural History), and consisted of house structures and hearths, stone and organic artefacts, human remains, and a large faunal assemblage [(Steffian 1992; Heizer 1956)](https://paperpile.com/c/GftuPk/EF8Q5+62auz). Hundreds of only the “best preserved” dog remains were collected, with excavators noting they were ubiquitous at the site. Excavation of the specimens was poorly recorded. Also, it is unknown in what context the dog remains were found, and whether they represent intentional burials, midden/refuse deposits, or some combination of deposition types. Thirty one specimens were sampled for DNA analysis, while 97 mandibles, 91 lower M1s and 15 crania from 133 unique specimens were used for the GMM analysis.

*\subsubsection*{Westwind, Lake Hazen, Ellesmere Island, Nunavut}*

*Extract ID: TJFD-3:1*

The Westwind (TjFd-3) site is an early Paleo-Inuit (Independence I) site related to the initial migration of humans into the high arctic regions of Canada and Greenland (ca. 3900 BP or 1950 BC). Survey of this area was undertaken by Patricia Sutherland within the boundaries of the Northern Ellesmere Island National Park Reserve between the late 1970s and the early to mid-1990s [(Sutherland 1996)](https://paperpile.com/c/GftuPk/jslUe). The site is located adjacent to Lake Hazen in the Eureka Upland, which is an intermontane plateaus is surrounded by extensive ice caps and mountains that range over 2700 m high. Faunal remains from this site and a nearby site similar in age were identified by Darlene Balkwill of the Canadian Museum of Nature, who reported that the vast majority of the remains are of muskox, hare, and fox [(Christyann Marie Darwent 2002)](https://paperpile.com/c/GftuPk/fje2b). Two Canis canines were reported from Feature 5, the most complete of which was sampled for mtDNA at the Canadian Museum of History.

### \subsection*{North American Inuit Sites}

*\subsubsection*{Aftermiut, Qaqortoq}*

*Extract ID:* *P95/2013KMG-Aftermiut-M1, Pxx/2013KMG-Aftermiut-M2, Pxx/2013KMG-Aftermiut-M6, P78/2013KMG-Aftermiut-M5, P79/2013KMG-Aftermiut-M7*

The Thule Inuit site of Aftermiut (ZMK 13/1934) is located at Qaqortoq (formerly Julianehåb) in the southern tip of Greenland. It was investigated by T. Mathiassen and E. Holtved in 1934 [(T. Mathiassen and Holtved 1936)](https://paperpile.com/c/GftuPk/4LaHE), and is estimated to date to ca. AD 1350-1650 [(Gullov 1997)](https://paperpile.com/c/GftuPk/lhkfj). A total of five dog mandibles were sampled for mtDNA at the Natural History Museum in Copenhagen.

*\subsubsection*{Ahteut, Kobuk River, Alaska}*
*Extract ID: UA1-1941-1856*

Ahteut (49-XBM-003) is a village site located in northwest Alaska along the middle portion of the Kobuk River between the present-day villages of Ambler and Kiana. The site has designated north and south components consisting of over 100 house features. James Louis Giddings excavated 12 houses at the site in the 1940s and dated a single house (3n) to a range between AD 1202 and 1250 based on five tree-ring dates [(J. Louis Giddings 1952)](https://paperpile.com/c/GftuPk/RIzAU). House 10s is the only other dated feature from Ahteut. Two different caribou metapodial scrapers were selected from the museum collection, sampled, and radiocarbon dated returning nearly identical results of 800-680 cal BP [(Shirar 2011)](https://paperpile.com/c/GftuPk/zwsRy). The single dog mandible sampled for DNA analysis and included in this study also originates from Giddings’ House 10s.

*\subsubsection*{Beulah, Cape Krusenstern, Western Coronation Gulf, Northwest Territories}*

*Extract ID: NCPF-12:2-18*

The Beulah site (NcPf-12) is located on the west side of the Cape Krusenstern peninsula, on the mainland coast of western Coronation Gulf. The site is comprised of 12, single-roomed, ovate winter houses arranged in two clusters with the entrance tunnels oriented to the southwest [(D. A. Morrison 1983)](https://paperpile.com/c/GftuPk/dtiX6). Limited excavation was undertaken in the “wall midden” of House 4, and a single dog mandible from was sampled for mtDNA at the Canadian Museum of History. Like the Clachan and Nuvuk sites, Beulah is early Thule (AD 1200–1400) in age [(D. A. Morrison 1983)](https://paperpile.com/c/GftuPk/dtiX6).

*\subsubsection*{Bonasila, Lower Yukon River, Alaska}*

*Sample ID: NMNH-254782-1, NMNH254781*

The site of Bonasila (49-XHC-047) is located on the banks of the Yukon River below the village of Anvik, near the confluence of the Bonasila and Yukon rivers in central Alaska. The site was surveyed by an expedition lead by Aleš Hrdlička, and is described as consisting of pit and tunnel dwellings with a cemetery [(Hrdlicka 1928)](https://paperpile.com/c/GftuPk/f3qiz). Though the primary target of the survey was the collection of human remains, the fossilised remains of many animals were noted and collected during the survey, including dogs, though their context was not recorded. A mandible and a lower M1 from Bonasila were included in the GMM analyses.

*\subsubsection*{Cache Point, Richards Island, Mackenzie Delta, Northwest Territories}*

*Extract ID: NHTS-2:C6-9-4, NHTS-2:C8-P1, NHTS-2:C8-P2*

The Cache Point site (NhTs-2) is situated on a large bluff on the east coast of Richards Island in the mouth of the Mackenzie Delta, Northwest Territories; at least 23 semi-subterranean Thule houses were documented [(T. M. A. X. Friesen 2009)](https://paperpile.com/c/GftuPk/iv6Du). To understand the history of Inuvialuit in this area, excavations were undertaken in 1996, 1998, and 1999 under the direction of Max Friesen. Diagnostic artifacts and radiocarbon dates place this site within the early Thule period at AD 1200–1300. House 6 was built of adzed logs and planks; it had a main room (the floor was compact sand rather than wooden planks), a side kitchen alcove, and an entrance tunnel [(T. M. A. X. Friesen 2009)](https://paperpile.com/c/GftuPk/iv6Du). House 8 was a somewhat more complex wooden structure that had a roughly square main room, the back half which appears to have been a bench—the planks may have been removed when the house was abandoned; the floor in front of the bench was still covered with large driftwood logs [(T. M. A. X. Friesen 2009; T. M. Friesen and Betts 2006)](https://paperpile.com/c/GftuPk/iv6Du+CtcqD). This house also had a kitchen tunnel connecting it to a kitchen, a side annex, and an external cache. Two right adult dog pelves—both carnivore gnawed—were selected from House 8, Level 3 at the Prince of Wales Northern Heritage Center in Yellowknife, and an adult femur from House 6, Level 3 was provided by Max Friesen for mtDNA sampling.

*\subsubsection*{Cape Espenberg, Seward Peninsula, Kotzebue Sound, Alaska}*

*Sample ID: BELA-36034*

*Extract ID: BELA-34679, BELA-35135, BELA-36031, BELA-36034, BELA-36035, BELA-37372, BELA-37373, BELA-37374, BELA-37375, BELA-37376, BELA-37940, BELA-59973, BELA-59974, BELA-37370, BELA-37371, BELA-52965, BELA-52966, BELA-37367, BELA-37368, BELA-37369,BELA-59975, BELA-59976, BELA-59977, BELA-59978, BELA-59979, BELA-59980, BELA-59981, BELA-59982*

Cape Espenberg is a long, narrow spit of land on the northwestern extent of the Seward Peninsula, the shores of which are bounded by the Chukchi Sea and Kotzebue Sound. It is part of Bering Land Bridge National Park and consists of a series of dune-covered beach ridges that jut out into the sound and have been building over the past 4000 years. Extensive mapping of the site was undertaken in 2007 and 2010 [(J. Darwent et al. 2013)](https://paperpile.com/c/GftuPk/FV5Vq); between 2009 and 2011, excavation of six Thule house depressions was directed by John Hoffecker and Owen Mason [(Alix et al. 2015; J. Darwent et al. 2013; Norman et al. 2017)](https://paperpile.com/c/GftuPk/Xa015+FV5Vq+TKoet). *Canis* specimens were sampled for mtDNA from across three sites at Cape Espenberg ([(Sarah K. Brown, Darwent, and Sacks 2013)](https://paperpile.com/c/GftuPk/61pmw):1)) 49-KTZ-304, Feature 12 and 21; 2) 49-KTZ-087, Feature 68A, 68B, and 87; 49-KTZ-088, Feature 33.

At KTZ-304, the oldest structure is Feature 12; a driftwood and whale-bone structure that revealed at least two to three occupations superimposed with periods of abandonment and rebuilding. It compares most closely to Birnirk structures described to the north at Barrow [(Alix et al. 2015)](https://paperpile.com/c/GftuPk/Xa015). The structure appears to have been built initially by Birnirk inhabitants, but then rebuilt or reused by early Thule occupants. Bone samples were collected from the upper levels (Level 1A-C and 2B) where associated radiocarbon dates span AD 1050–1300 [(J. Darwent et al. 2013)](https://paperpile.com/c/GftuPk/FV5Vq). Right mandibles were sampled from Level 1 (BELA-59976, 59977, 59978, 59980, 59982) and Level 2 (BELA-59975, 59979, 59981). Sample BELA-59981 is a large wolf mandible, which was confirmed by aDNA testing. Feature 21 at this site is a typical Thule structure built of driftwood logs and planks with a main room, a side-spur kitchen area full of burned and cemented fat [(Buonasera et al. 2015)](https://paperpile.com/c/GftuPk/HnBDR), and long entrance tunnel. Based on radiocarbon dates the structure was occupied from AD 1250–1400. A wolf metacarpal (BELA-36031), three dog crania (BELA-36034, 36035, 37372), and four dog mandibles that did not match the crania (BELA-37373, 37374, 37375, 37376) from the lower levels of the tunnel, kitchen side spur, and house interior were all sampled for mtDNA.

The remains of three driftwood constructed Thule houses were excavated at KTZ-087. Features 68A and 68B were located adjacent to each other only one meter apart; 68A was excavated in 2010 and 68B in 2011; both structures radiocarbon date to AD 1450–1650 (Brown et al. 2013; Darwent et al. 2013). Feature 68A is a typical Thule dwelling with a square main room with planks forming the rear sleeping platform, a hearth area to the front, and then a 4-m long entrance tunnel. A separate external kitchen area with no connection to the house was also excavated [(J. Darwent et al. 2013)](https://paperpile.com/c/GftuPk/FV5Vq). A cranium and non-matching mandible from Level 3 of the floor of the tunnel/cold trap entrance were sampled (BELA-37370, 37371). Feature 68B is nearly identical in terms of the construction of the tunnel, but the main room was distinguished by a beam running perpendicular to the tunnel and a compacted sand floor visible in the wall profile. The main area of the house was full of wood-working debris, as well as bone and stone debitage, and thus has been interpreted as a communal men’s house. Two crania were sampled from the tunnel floor (BELA-52965, 52966), one of which had evidence of chop marks to remove the parietal/occipital region and cut marks indicative of skinning on the frontal.

The third feature excavated at KTZ-087 was Feature 87, which radiocarbon dates to AD 1300–1450 [(Sarah K. Brown, Darwent, and Sacks 2013; J. Darwent et al. 2013; Norman et al. 2017)](https://paperpile.com/c/GftuPk/61pmw+FV5Vq+TKoet). This semi-subterranean wood-framed structure included a square main room with a wide rear platform and two narrow benches along the side walls [(Norman et al. 2017)](https://paperpile.com/c/GftuPk/TKoet). An amorphous area of burned material and cemented sand was located to the front of the house and was likely a kitchen alcove. A long tunnel served as the entrance. Faunal remains are dominated by small seal at 82\% followed distantly by caribou and dog at 3\% each [(Norman et al. 2017)](https://paperpile.com/c/GftuPk/TKoet). A left, fifth metacarpal from Level 2 of the midden area in front of the tunnel (BELA-59974) and from Level 3 of the main room/entrance (BELA- 59973) was sampled, along with a calcaneus from Level 2 outside and just to the east of the house (BELA-35135). Two modified maxillary canines that appear to have been used as pendants were also sampled: one from Level 2 (BELA-34679) at the rear of the house, and one from inside a ceramic vessel in the kitchen (BELA-37940).

Only Feature 33 was excavated at KTZ-088, which is a well-built Inupiat house dating to the 18^th^ century based on radiocarbon dating of structural wood and caribou bone [(Méreuze 2015; Sarah K. Brown, Darwent, and Sacks 2013; J. Darwent et al. 2013)](https://paperpile.com/c/GftuPk/VpS6n+61pmw+FV5Vq). The structure was occupied pre-contact as no Russian-American trade items were recovered. Entrance to the main room was through a tunnel made of driftwood capped with a whale bone lintel. An external kitchen with thick layers of burned and cemented sand [(Buonasera et al. 2015)](https://paperpile.com/c/GftuPk/HnBDR) was also excavated. Wood flooring was in place in the front of the room where a ceramic lamp also remained, but the sleeping platform planks appear to have been removed on abandonment. Three right ulnae were sampled from the tunnel (BELA-37367), kitchen (BELA-37368), and in front of the sleeping platform (BELA-37369) for mtDNA.

*\subsubsection*{Cape Garry, Somerset Island, Nunavut}*

*Extract ID: PCJQ-5:H5-M1, PCJQ-5:H5-M3, PCJQ-5:H6-M1, PCJQ-5:H6-M2, PCJQ-5:H6-M3, PCJQ-5:H6-M4, PCJQ-5:H6-M6*

Three Thule houses were excavated at Cape Garry (PcJq-5) on the southernmost extent of Creswell Bay, Somerset Island, under the direction of Allen McCartney in 1976. Faunal remains from this House 6 at this site, and House 1 at the Learmonth site, nearly 40 km to the north, were analyzed by Anne Rick [(Rick 1980)](https://paperpile.com/c/GftuPk/6eg5N). House 6 was a roughly circular-shaped stone-and-sod Thule dwelling with a single platform; a small alcove had been added to the room at a later date. Two right mandibles from House 5 and five left mandibles from House 6 were sampled at the Canadian Museum of History for mtDNA.

A caribou rib from House 5 and a long-bone shaft fragment from House 6 were submitted by C. Darwent for radiocarbon dating at Arizona AMS. The results were nearly identical with calibrated ages of AD 1305–1445 (AA102351: 540 ± 45 BP, d13C -19.9‰) and AD 1300–1450 (AA102352: 528 ± 55 BP, d13C -18.4‰) respectively, which fits well with Classic Thule for this region.

*\subsubsection*{Cape Krusenstern, Kotzebue Sound, Alaska}*

*Extract ID: CK-H4-M1, CK-H4-M2, CK-H4-M3, CK-H4-M4, CK-H4-M5, CK-H4-M6, CK-H5/7-M1, CK-H5/7-M2, CK-H6-M1, CK-H6-M2, CK-H6-M3, CK-H6-M4, CK-H6-M5, CK-H6-M6, CK-H6-M7, CK-H6-M8, CK-H6-M9, CK-H6-M10, CK-H8-M1, CK-H8-M2, CK-H8-M3, CK-H8-M4, CK-H8-M5, CK-H8-M6, CK-H8-M7, CK-H8-M8, CK-H8-M9, CK-H8-M10, CK-H8-M11, CK-H8-M12, CK-H8-M13, CK-H32-M1, CK-H32-M2, CK-H33-M1, CK-H33-M2, CK-H35-A1, CK-H50-M1*

Cape Krusenstern was designated as an Archaeological District (49-NOA-002) in 1973 because it contains a historical record of human habitation of these beach ridges for more than 5,000 years. Discovery of this rich archaeological record, known locally as Sealing Point, came late in the 1958 field season for J.L. Giddings [(J. Louis Giddings and Anderson 1986)](https://paperpile.com/c/GftuPk/grz9v). Excavation of Thule-period dwellings was undertaken by Giddings from 1959 to 1961. Artifacts and faunal remains from these excavations are housed at the Haffenreffer Museum of Anthropology at Brown University.

The remains of nearly 30 Kotzebue period or late Thule houses were mapped along one of the youngest beach ridges at Cape Krusenstern. Excavation was undertaken in House 14, 31, 34, 35, and 50. House 50, which was part of a cluster of four houses, was a single-roomed dwelling with a long entrance tunnel and no evidence of a raised sleeping platform [(J. Louis Giddings and Anderson 1986)](https://paperpile.com/c/GftuPk/grz9v). A mandible was selected for DNA sampling. House 35 was clustered with the ruins of one other house and they may have originally been joined; these are the smallest houses from this time period. House 35 was a nearly square single-roomed house with an entrance tunnel extending from the south end of the front wall ([(J. Louis Giddings and Anderson 1986)](https://paperpile.com/c/GftuPk/grz9v): 50). An atlas was selected for DNA testing. Radiocarbon dating and artifact styles indicate these houses were most likely occupied from AD 1400 to 1600.

Five house depressions were excavated that date to Early Western Thule settlement of Cape Krusenstern; houses 4 and 5 cluster on beach 10 and houses 6, 7, and 8 on beach 11 [(J. Louis Giddings and Anderson 1986)](https://paperpile.com/c/GftuPk/grz9v). Constructed of driftwood and sod, House 4 had a square main room, a short tunnel leading to a kitchen, and a longer entrance tunnel [(J. Louis Giddings and Anderson 1986)](https://paperpile.com/c/GftuPk/grz9v); five adult, and one subadult, dog mandibles were sampled for DNA. House 6 was somewhat larger, and better preserved than House 4, but the overall construction was similar; six adult, two subadult, and two juvenile dog mandibles were sampled for DNA. House 5 and House 7 were similarly constructed houses with a long tunnel entering a main room with a rear sleeping platform, a short tunnel and kitchen angling off the side of the main room, and a side room with planked floor was entered through a side tunnel off the main entrance tunnel [(J. Louis Giddings and Anderson 1986)](https://paperpile.com/c/GftuPk/grz9v). The faunal remains from houses 5 and 7 were commingled; two adult mandibles were selected for DNA sampling. House 8 consisted of one main room with a rear platform of driftwood planks, a large kitchen extending directly off of one of the front corners, and a long entrance tunnel [(J. Louis Giddings and Anderson 1986)](https://paperpile.com/c/GftuPk/grz9v); 12 adult, and one juvenile, dog mandibles were sampled for DNA. Based on radiocarbon dates, artifact styles, and comparison to similar sites in the region, the estimated age of these Early Western Thule houses is AD 1000–1250 [(J. Louis Giddings and Anderson 1986)](https://paperpile.com/c/GftuPk/grz9v).

House 32 and 33, which had been deeply buried by formation of the subsequent beach ridge deposit and only visible by two crescent-shaped depressions, were excavated in 1960 and 1961 respectively [(J. Louis Giddings and Anderson 1986)](https://paperpile.com/c/GftuPk/grz9v). These two dwellings compared with Birnirk structures excavated at other northern Alaska coastal sites, and radiocarbon dates indicate they were occupied AD 650–1150 [(J. Louis Giddings and Anderson 1986)](https://paperpile.com/c/GftuPk/grz9v). House 32 had a main room with a hearth area on the floor in front of the entrance tunnel; the tunnel which extended perpendicular to the dwelling had a planked floor and a small side alcove. House 33 had a similarly sized main room, but the entrance tunnel extended diagonally out from the main room and a plank-floored alcove extending from the front corner of the main room. Both houses had planked side platforms that extended across half of main room—in other words, oriented 180⁰ to later Western Thule-style dwellings. C. Darwent undertook analysis of the faunal remains from these houses, and two adult mandibles from each of the houses were sampled for mtDNA.

*\subsubsection*{Cape Nome, Seward Peninsula, Norton Sound, Alaska}*

*Extract ID: UA2001-75-1, UA2001-75-2*

Two dog skulls were collected by Otto Geist at Cape Nome in 1946. Primary documentation notes the specific locality as “Nome, recent Eskimo village, sandspit across from Nome,” but no other contextual details are provided. Both of these specimens were sampled for this project. The skulls are likely affiliated with the Inupiat ca. AD 1800-1900.

*\subsubsection*{Clachan, Cape Hearne, Western Coronation Gulf}
Extract ID: NAPI-2:14, NAPI-2:15-38-21, NAPI-2:16, NAPI-2:19, NAPI-2:28-15-6, NAPI-2:29-1b-5, NAPI-2:33-17, NAPI-2:33-8, NAPI-2:C70-10*

The Clachan site (NaPi-2) is located just south of Cape Hearne on the mainland coast facing Coronation Gulf, which is an area known locally as *Kugalyuk*. Archaeological investigation of this site (along with Beulah and Nuvuk) was undertaken by David Morrison between 1979 and 1981 [(D. A. Morrison 1983)](https://paperpile.com/c/GftuPk/dtiX6). A cluster of three, stone-and-sod Thule winter houses were excavated, from which a sample of 18 individual dog mandibles were analyzed osteometrically [(D. A. Morrison 1983)](https://paperpile.com/c/GftuPk/dtiX6). Morrison focused on carnassial length, tooth row length, and total mandible length. Bivariate scatterplots indicate the Clachan site dogs are smaller, on average, than modern Inuit sled dogs, and both are smaller than the northern Mackenzie wolf. Since all of these mandibles were stored together at the Canadian Museum of History, we were able to extract samples from nine of the 18 individuals. A series of 10 spruce wood and or willow twig samples were radiocarbon dated. Together the dates range from ca. AD 700 to 1200, which is 300–400 years too old for an early Thule site in Canada [(D. A. Morrison 1983)](https://paperpile.com/c/GftuPk/dtiX6) and likely the result of seal oil contamination. Commingled with the dog mandible samples was a caribou premolar from the excavated houses, which C. Darwent submitted for radiocarbon dating from Arizona AMS: 700 $\pm$ 46 BP, d13C -17.5‰ (AA102348). The calibrated age fits with Early Thule sites for the western Canadian Arctic at AD 1240–1400.

*\subsubsection*{Deering, Seward Peninsula, Kotzebue Sound, Alaska}*

*Extract ID: DRG-99-0043, DRG-99-0769, DRG-99-1196*

Deering is a northwestern Native Alaskan community situated on a spit within Kotzebue Sound on the northern coast of the Seward Peninsula. Several archaeological sites are located along the Deering spit that make up the Deering Archaeological District (Alaska Heritage Resources Survey number KTZ-000169) [(Peter M. Bowers and Williams 2009)](https://paperpile.com/c/GftuPk/8LcB2). Specimens DRG-99-0043, DRG-99-0769, DRG-99-1196 come from the Western Thule House 1 (KTZ-00300). The occupation of the Western Thule House 1 dates between AD 720 and 970.

*\subsubsection*{Dødemandsbugten, Clavering Island}*

*Extract ID: TRF-01-09(ZMK 28/1932)*

Dead Man’s Bay, also known as Dødemandsbugten, is located on Clavering Island in North Eastern Greenland [(Sørensen and Gulløv 2012)](https://paperpile.com/c/GftuPk/aRAdo). Settlement of the site took place between the fifteenth and nineteenth centuries CE [(Morey 2010)](https://paperpile.com/c/GftuPk/9nIm2). Forty-three house ruins were identified by Danish archaeologist Helge Larsen, twenty-three of which were excavated [(Morey 2010)](https://paperpile.com/c/GftuPk/9nIm2). Additionally, thirty graves were detected during the excavation as well as indications of seven interments within one of the houses [(Larsen and Sørensen 1934)](https://paperpile.com/c/GftuPk/vKntg). The site likely has two periods of occupation: stage 1, between the 15th and 17th centuries, and stage 2, between the 18th and 19th centuries CE [(Gotfredsen 2010)](https://paperpile.com/c/GftuPk/Oa3Pg). The demise of the early occupation has been suggested to be the result of starvation seen through the recovery of humans remains within a house, the ‘disproportionate’ abundance of dog bones inside and around some houses, and gnaw marks attributed to humans on some of the well preserved dog remains which [(Larsen and Sørensen 1934; Morey 2010)](https://paperpile.com/c/GftuPk/vKntg+9nIm2). Material from this site includes ancient DNA from the petrous bone of a skull from one of the early phase houses.

*\subsubsection*{Double Mer Point, Labrador}*

*Extract ID: TRF-07-03(DMP04), TRF-07-04(DMP07), TRF-07-08(DMP15), TRF-07-12(DMP24)*

The Double Mer Point site is an 18th century Inuit village located in central Labrador, Canada. The site was initially investigated by William Fitzhugh in 1968, and then tested by Richard Jordan of Bryn Mawr College in 1973. Since 2013 Lisa Rankin and graduate students from Memorial University of Newfoundland have undertaken a near complete excavation of the three semi-subterranean houses and surrounding middens in partnership with the nearby Inuit community of Rigolet, Nunatsiavut. Multiple dog bones have been recovered from the site. DNA was extracted from a mandible recovered from the midden between House 1 and House 2 ([(Rankin 2014)](https://paperpile.com/c/GftuPk/syJ9e) and pers. comm. L. Rankin, 2019).

*\subsubsection*{Eskimobyen, Knud Peninsula, Ellesmere Island, Nunavut}
Extract ID: SGFM-4:H14-C1, SGFM-4:H14-C2, SGFM-4:H14-C3, SGFM-4:H20-M*

Sites in the Bache Peninsula region were investigated by members of the Arctic Institute’s Ellesmere Island Research Project under the direction of Peter Schledermann between 1977 and 1982 [(Schledermann and McCullough 2003)](https://paperpile.com/c/GftuPk/rQxj4). The Eskimobyen site (SgFm-4) is located on the Knud Peninsula and separated from the Bache Peninsula by Flagler Bay; 26 Thule winter stone-and-sod house structures were identified at this site [(Schledermann and McCullough 2003)](https://paperpile.com/c/GftuPk/rQxj4). Three dog cranial fragments from House 14 and one mandibular fragment from House 20 were sampled for mtDNA at the Canadian Museum of History. Both of these houses are Ruin Island style and thus are associated with early Thule (AD 1200–1300) in the Smith Sound region of Ellesmere Island and northern Greenland. A caribou rib fragment from House 14 was submitted by C. Darwent for radiocarbon dating with Arizona AMS (AA102355: 1229 $\pm$ 47 BP, d13C -14.2). Although the rib was clearly from a caribou, the isotopic value was highly marine; the calibrated age for the sample using a marine correction is AD 1060–1273. One end of the correction fits with the estimated age of this site at AD 1200–1300.

*\subsubsection*{Fort Churchill, Manitoba}*

*Extract ID: TRF-01-31(ZMK 28/2017), TRF-01-59(ZMK 27/2017)*

Pre-Dorset, Dorset, and subsequent Thule settlements have all had phases of occupation in the Fort Churchill area on the west coast of the Hudson Bay in Manitoba, Canada [(Ronald J. Nash 1972; Riewe, R., Suluk, L., & Brandon, L 1989)](https://paperpile.com/c/GftuPk/Wtov4+iOaum). The pre-Dorset occupation is considered to be much later than other pre-Dorset sites, such as those in Igloolik, with radiocarbon dating placing the occupation around 945 BCE. [(R. J. Nash 1976)](https://paperpile.com/c/GftuPk/XN97S). The later Dorset occupation has been radiocarbon dated to approximately 130 BCE [(R. J. Nash 1976)](https://paperpile.com/c/GftuPk/XN97S). DNA was extracted from petrous bones from two dog skull recovered from a partially buried specimen and a specimen found on the surface of the Fort Churchill site, the presumed dates of the individuals are of more recent Thule occupations from within the last 200 years.

*\subsubsection*{Gambell, St. Lawrence Island, Alaska}*

*Sample ID: NMNH\_254868, NMNH\_258749, NMNH\_258751, NMNH\_258758\_1, NMNH\_258761\_1, NMNH\_258761\_2, NMNH\_258761\_3, NMNH258749, NMNH258761\_1, NMNH258771-2, NMNH258771-8, NMNH258758\_3*

The Gambell site is located at the Northwest tip of St Lawrence Island in the Bering Sea. The site consists of a set of stratified mounds, with the oldest occupations dating to the Old Bering Sea cultural phase [(J. L. Giddings 1960)](https://paperpile.com/c/GftuPk/UrmPd). Material from this site included in this study are 7 mandibles and 6 lower M1s from a total of 11 specimens.

*\subsubsection*{Geographical Society Islands, Northeast Greenland}*

*Extract ID: P88/2013KMG-GeoSociety-M6, P89/2013KMG-GeoSociety-M5, P90/2013KMG-GeoSociety-M7, P91/2013KMG-GeoSociety-M4, P92/2013KMG-GeoSociety-M2, P93/2013KMG-GeoSociety-M1, P94/2013KMG-GeoSociety-M3*

Several Thule period winter house structures (ca. AD 1500–1800) located in the King Oscar Fjord region of Northeast Greenland were excavated by P. V. Glob [(Glob 1935)](https://paperpile.com/c/GftuPk/5nUzj) in the early 1930s (ZMK 17/1933). A total of seven right mandibles from adult dogs, originally analyzed by zoologist Magnus Degerbøl were sampled for DNA at the Natural History Museum in Copenhagen.

*\subsubsection*{Gutchiak, Eskimo Lakes, Mackenzie Delta, Northwest Territories}*

*Extract ID: NHTN-1:2023M, NHTN-1:2036H, NHTN-1:2048H, NHTN-1:2099M, NHTN-1:2133H*

The Gutchiak site (NhTn-1) is located on the edge of a low plateau, on the eastern side of a small tidal lagoon, at the tip of one of a series of long peninsulas that divide up the Eskimo Lakes like accordion folds. Gutchiak means “like a river” and is located in a place where strong tidal currents ensure late freeze-up and early break-up, and thus an ideal location for netting fish [(D. Morrison 2000)](https://paperpile.com/c/GftuPk/bCJ0d). The faunal remains—dominated by fish bone—were recovered from cultural deposits interpreted as a procurement/processing site; hearth areas and the remains of posts suggest the construction and use of a smoking tent for drying fish [(D. Morrison 2000)](https://paperpile.com/c/GftuPk/bCJ0d). The deposits span the precontact Thule/Inuvialuit period from approximately AD 1400 to 1800 [(Matthew W. Betts 2008; D. Morrison 2000)](https://paperpile.com/c/GftuPk/nzphP+bCJ0d). Two adult mandibles and four juvenile humeri were sampled at the Prince of Wales Northern Heritage Centre in Yellowknife for mtDNA.

*\subsubsection*{Haa Island, Hayes Fjord, Ellesmere Island, Nunavut}*

*Extract ID: SGFQ-1:H9-1, SGFQ-1:H9-2; SGFQ-1:H24-F1*

Sites in the Bache Peninsula region were investigated by members of the Arctic Institute’s Ellesmere Island Research Project under the direction of Peter Schledermann between 1977 and 1982 [(Schledermann and McCullough 2003)](https://paperpile.com/c/GftuPk/rQxj4). The Haa Island site (SgFq-1) is described as a precipitous rocky island located at the junction of Beitstad and Jokel Fjords within the larger Hayes Fjord on eastern Ellesmere Island, Nunavut [(Schledermann and McCullough 2003)](https://paperpile.com/c/GftuPk/rQxj4). On the west side of the island a broad grassy slope holds the remains of 20 stone-and-sod Thule winter houses; it would have been a prime location for breathing-hole sealing. Metacarpals III-IV from an adult dog and a cranium/mandible from a juvenile dog were sampled from House 9, and a fibula was sampled from House 24 for mtDNA at the Canadian Museum of History. A radiocarbon date on heather recovered from House 24 yielded a corrected age of ca. AD 1450 [(Schledermann and McCullough 2003)](https://paperpile.com/c/GftuPk/rQxj4). Both of these dwellings date to the post-Ruin Island phase of Thule occupation in this region, or what would be called Classic or middle Thule elsewhere.

*\subsubsection*{Hebron, Labrador}*

*Sample ID: HMCZ\_7406*

Hebron is located on the Northeastern coast of Labrador, Canada. Archaeological excavations at Hebron indicate the occupation of Moravian Inuit peoples during the 19th century [(Loring and Arendt 2009)](https://paperpile.com/c/GftuPk/qNdOI), though there is indication that the site was occupied prior to the historic period, as Moravian missionaries in the early twentieth century uncovered large sod houses, stone burial chambers, and Inuit and Paleo-Inuit artefacts which were transported to museums and collections [(Loring and Arendt 2009)](https://paperpile.com/c/GftuPk/qNdOI). In his book “Dogs of the American Aborgonies” Glover Allen mentions “the skull of an Eskimo Dog from Hebron, Labrador, collected in 1897” ([(Allen 1920)](https://paperpile.com/c/GftuPk/0fGFw) pg. 443), which is now held in the collections at the Harvard Museum of Comparative Zoology. Both the cranium and mandible were used in this study for GMM analysis.

*\subsubsection*{Herschel Island, Yukon}*

*Sample ID: HMCZ\_57575*

Herschel Island lies off the Yukon Coast, west of the mouth of the Mackenzie River in Northwestern Canada. Evidence of human occupation comes from a stretch of rapidly

eroding shoreline on the northeast edge of the island, including the Thule period Washout Site (NjVi-2) [(Yorga 1980)](https://paperpile.com/c/GftuPk/czw1j). Excavations in 1978 recovered a large quantity of well preserved midden materials, and included round and chipped stone tools, pottery, bone artefacts, and a wide range of organics including bird and fish bones, scales, feathers, hair, hide, gut, sinew, coprolites, and baleen [(Morgan et al. 1983)](https://paperpile.com/c/GftuPk/gT1fP). A single lower M1 from this site was included in the GMM analysis, and is now held at the Harvard Museum of Comparative Zoology.

*\subsubsection*{Hopedale, Labrador}*

*Sample ID: HMCZ\_44669*

The Hopedale region of Labrador, Canada lies on the southeastern coast of the province. Archaeological survey and excavations began there as early as 1935 with who examined five sites in the region, and excavated 45 of the recorded 88 Inuit sod houses, providing early evidence for the Thule Inuit occupation in Labrador [(Arendt 2013)](https://paperpile.com/c/GftuPk/zzfbP). Since then, various excavations throughout the twentieth century have refined our understanding of the Thule occupation of Labrador, and Hopedale specifically, which radiocarbon dates indicating that the Thule arrived in the region during the 16th century, and genetic evidence indicating that they displaced early pre Thule peoples [(Raghavan et al. 2014)](https://paperpile.com/c/GftuPk/y69GU). A large number of faunal remains were found in and around house structures, as were the remains of a dog sled [(Arendt 2013)](https://paperpile.com/c/GftuPk/zzfbP). For this analysis two specimens were analysed for GMM analysis, one providing a mandible, lower M1 and crania, and the other a single crania.

*\subsubsection*{Iglulualuit, Cape Bathurst Peninsula, Mackenzie Delta, Northwest Territories}*

*Extract ID: NLRU-1:623C2, NLRU-1:625M1, NLRU-1:626M2*

Iglulualuit (Nlru-1) is located on the Cape Bathurst Peninsula near the mouth of the Horton River Delta on Franklin Bay in the Western Canadian Arctic; it was excavated by David Morrison in 1987 [(D. Morrison 1990)](https://paperpile.com/c/GftuPk/St2Xq), and the collections are stored at the Canadian Museum of History. Iglulualuit means “many houses” and is one of the largest sites in the Canadian Arctic with at least 30 Thule/Inuvialuit winter houses spread across 800 m of coast line. The two excavated houses, House 11 and House 20, were constructed mainly out of driftwood timbers with the occasional bowhead whale element or rocks [(D. Morrison 1990)](https://paperpile.com/c/GftuPk/St2Xq). *Canis* remains were recovered from excavation of House 11 (AD 1500–1800), but comprised only 1\% of the faunal assemblage (Morrison 1990). A fragmentary adult cranium and two sets of juvenile mandibles were sampled for mtDNA.

*\subsubsection*{Iita (Etah), Foulke Fjord, Inglefield Land, Greenland}*

*Extract ID: KNK2643x1838, KNK2643x1839, KNK2644x1119, KNK2644x1120, KNK2644x1121*

Iita is a multicomponent Inughuit (Inuit) site located on an alluvial fan jutting from the northern shore of Foulke Fjord, Inglefield Land, northwestern Greenland. This site, also known as Etah in the historical literature, was the nearest Inughuit community to a number of over-wintering expeditions, the earlier being those of Elisha Kent Kane (1856) and Isaac Israel Hayes (1867) in the mid-1800s, and the latter associated with Robert Peary’s North Pole expedition (1898–1902). Donald MacMillan’s Crocker Land expedition 1913–17 was set up at Iita itself. The surface of the alluvial fan has over 180 visible features present—including the remains of nine winter houses, two of which were excavated in 2006 [(LeMoine and Darwent 2010)](https://paperpile.com/c/GftuPk/ig5DN). Most of the surface features relate to late prehistoric and early historical period Inughuit occupations, but subsurface components are also present due to unique geological features that have created a series of buried strata and include occupations extending to AD 1000 and the Late Dorset period (Paleo-Inuit)—highly unusual for the High Arctic. However, dog remains were only recovered from Thule-Inughuit components [(Sarah K. Brown, Darwent, and Sacks 2013; Johansen 2012)](https://paperpile.com/c/GftuPk/61pmw+2Q6eB). Two juvenile dog mandibles were sampled for DNA from winter house Feature 1: KNK2643x1838 from early Thule levels dated to AD 1275–1650, and KNK2643x1839 from historical Inughuit levels dating from 1900–1910, coinciding with Peary’s expedition. Mandibles from a juvenile, adult, and old adult dog were sampled for DNA from historical Inughuit levels in winter house Feature 2 dated to AD 1850–1920 (KNK2644x1119, x1120, and x1121). Thus these samples span the early Thule to Historic habitation of this site by the Inughuit and their ancestors.

*\subsubsection*{Inugsuk, Upernavik, Greenland}*

*Extract ID: TRF-01-25(ZMK 53b/1966), TRF-01-26(ZMK 53a/1966), TRF-01-45(ZMK 20/2017), P100/2013KMG-Inugsuk-C9, P101/2013KMG-Inugsuk-C1, P102/2013KMG-Inugsuk-C5, P103/2013KMG-Inugsuk-C8, P104/2013KMG-Inugsuk-C6, P106/2013KMG-Inugsuk-C4*

Inugsuk is an island approximately 20 kilometers north of Upernavik in western Greenland which contains a Thule age site of the same name. Much of the site, primarily the middens, had been washed away to sea through erosion before the excavation [(Therkel Mathiassen 1930a)](https://paperpile.com/c/GftuPk/2zmJW). The topmost layer of the site contains materials from occupation phases in the eighteenth and nineteenth centuries CE[(Therkel Mathiassen 1930a)](https://paperpile.com/c/GftuPk/2zmJW). Conditions on the site allowed for preservation of organic materials such as wood, baleen, narwhal tusk, and whale bone, among the preserved materials sledge crossbars, trace buckles and whip handles were recovered [(Therkel Mathiassen 1930b, [a] 1930)](https://paperpile.com/c/GftuPk/Q61Eb+2zmJW). Samples were included from the site were derived from the oldest phase, a surface find, and a partially buried skull from the upper layers of the site.

*\subsubsection*{Iyatayet, Cape Denbigh, Norton Sound, Alaska}*

*Extract ID: IYAT-00192*

The Iyatayet site (49-NOB-002), located on Cape Denbigh in Norton Sound, Alaska, was investigated by Andrew Tremayne in 2013 [(Tremayne et al. 2018)](https://paperpile.com/c/GftuPk/7VdTG). The dog mandible was recovered from beach deposits eroding from the Nukleet (Thule) layers and dates to ca. AD 1100-1400.

*\subsubsection*{Kitnepaluk, St. Lawrence Island, Alaska}*

*Extract ID: KP-1, KP-2, KP-3*

Kitnepaluk is located on the west coast of St. Lawrence Island approximately 25 km south of Gambell. The site consists of a small rocky midden in addition to two cultural mounds that include midden deposits as well as house and cache features. In 1972 and 1973, as part of a multiyear research program run through the University of Bern, Hans-Georg Bandi excavated two trenches designated as K1 and K2. K1 was thirteen meters long, 1-1.5 meters deep, and was excavated through the mound on the northwest part of the site. K2 was twenty-five meters long, 2.9 meters deep, and was excavated through the mound on the southeast part of the site. Chronologically the site is estimated to date to the Punuk time period based on stylistic attributes of artifacts and two radiocarbon dates run on driftwood samples collected from two different house features revealed during excavation of K2 [(Bandi and Blumer 2002; Mason 2016)](https://paperpile.com/c/GftuPk/IrP7o+PF0SF). Three complete dog mandibles collected from K2 were sampled for this study.

*\subsubsection*{Kittigazuit, Tuktoyaktuk Peninsula, Northwest Territories}*

*Extract ID: NITR-2:T1*

Investigation of the Thule site known as Kittigazuit site (NiTr-2) on Tuktoyaktuk Peninsula in the Mackenzie Delta region of the Northwest Territories was undertaken by Robert McGhee who excavated two Thule winter houses and associated midden areas here in 1969–70 [(McGhee 1975; Matthew W. Betts 2008)](https://paperpile.com/c/GftuPk/DGX60+nzphP). A tibia was sampled for mtDNA at the Canadian Museum of History. Previous radiocarbon dates suggest the site was occupied between AD 1300 and 1660; C. Darwent submitted a caribou metapodial fragment for dating at Arizona AMS (AA102349: 375 ± 45, d13C -19.1‰), which produced a calibrated age of AD 1445–1635.

*\subsubsection*{Kotzebue, Kotzebue Sound, Alaska}*

*Extract ID: KOTZ-10-0118A*

The Kotzebue Archaeological District (49-KTZ-036) is located within the city of Kotzebue, or Qikiqtaġruk, on Baldwin Peninsula that juts into Kotzebue Sound. The district consists of at least 53 sites, 47 historic and 16 prehistoric, including the large villages of Old Kotzebue (49-KTZ-031) and Intermediate Kotzebue (49-KTZ-030) sites. The KOTZ-10-0118A dog remains were recovered from a Late Prehistoric semi-subterranean house pit floor (Housepit 10). Housepit 10 was documented by Northern Land Use Research Inc. in 2010 [(Carlson et al. 2013)](https://paperpile.com/c/GftuPk/jCT1u). This structure is larger than many of the surrounding house pit structures and may represent a communal house, or \textit{*qalegi}*. The floor of Housepit 10 has yet to be radiocarbon dated. However, artifacts from the house floor reflect Late Prehistoric assemblages dating between AD 1400 and 1770.

*\subsubsection*{Kuk, Southampton Island, Nunavut}*

*Extract ID: TRF-01-32(ZMK 25/2017), TRF-01-33(ZMK 24/2017)*

The Kuk site (LjHp-2, 3, 4) is a Thule period site in Duke of York Bay on northwestern Southampton Island, Nunavut, Canada. Excavated initially by Therkel Mathiassen in the early 20th century, the site consists of three main groupings of semi-subterranean houses, containing traditional and well preserved Thule artefacts [(Therkel Mathiassen 1927b)](https://paperpile.com/c/GftuPk/WRVMv). The site is considered to be contemporary with other local Classic Thule sites dating to 1200 CE [(Clark 1980)](https://paperpile.com/c/GftuPk/SCk6l) and canid specimens were sampled for mtDNA sequencing.

*\subsubsection*{Kukulik, St. Lawrence Island, Alaska}*

*Extract ID: UA1-1933-9631, UA1-1933-9632, UA1-1937, UA1-1939-1497, UA1-1939-1497-1, UA1-1939-1497-2, UA1-1939-1497-3, UA1-1939-1497-4, UA1-1939-1497-5, UA2001-078-0004*

Kukulik is located on the northern coast of St. Lawrence Island and was the focus of extensive archaeological excavation in the early half of the 1930s. This work was conducted under the direction of Otto William Geist and Froelich Rainey [(Geist and Rainey 1936)](https://paperpile.com/c/GftuPk/7c6vu) between 1931 and 1933 as the Alaska College Bering Sea Expedition and the between 1934 and 1935 as the United States Department of Interior-Alaska College Archaeological Expedition. A test cut 41 meters long was excavated through the site between 1931 and 1933 which revealed numerous house and cache features as well as extensive midden deposits. This test cut also revealed approximately five meters of deposition proving the Kukulik Site to be a large cultural mound built up during the course of approximately 2000 years of human occupation. The test cut was completed in 1933 and work continued at the site in 1934 and 1935 to excavate large portions of the mound north and east of the test cut [(Houlette 2009)](https://paperpile.com/c/GftuPk/O8UAV). Dozens of dog specimens exist from this site and nine were sampled for DNA.

*\subsubsection*{Kuukpak (Gupuk), Richards Island, Mackenzie Delta, Northwest Territories}*

*Extract ID: NITS-1:4794C, NITS-1:A2H1M, NITS-1:A2H1R, NITS-1:A2H1T*

The Kuukpak site (NiTs-1), also known as Gupuk, is located on Richards Island facing the east channel of the Mackenzie River. The site is divided into six areas made up of spatially distinct clusters of houses; approximately 21 semi-subterranean house ruins extend across an 800 m stretch of beach [(Matthew W. Betts 2008)](https://paperpile.com/c/GftuPk/nzphP). House 1 in Area 1 is a cruciform-shaped semi-subterranean driftwood and sod structure that was excavated under the direction of Chuck Arnold in 1986 [(Arnold 1994; T. M. Friesen and Arnold 1995)](https://paperpile.com/c/GftuPk/9elav+Int0o); he returned in 2002 and placed several test pits to examine the impact of coastal erosion on the site. An adult canine was pulled from the faunal remains in Level 4 in Area 2, House 1’s midden at the Prince of Wales Northern Heritage Centre in Yellowknife for mtDNA sampling. Area 2, House 1 and a large midden area was excavated in 1986, 1988, and 1989. Portions of the midden, although exceptionally well-preserved in permafrost, were eroding into the East Channel. Analysis of 28,000 specimens represented only 30% of the massive midden assemblage [(Balkwill and Rick 1994)](https://paperpile.com/c/GftuPk/ttunl). Faunal remains from Area 2, House 1 were searched for DNA samples and the following were selected: 1) adult metacarpal from Level 3, 2) adult incisor and canine from Level 5, and 3) juvenile rib from Level 5. Kuukpak was occupied from AD 1300–1500 based on a series of radiocarbon dates and temporally sensitive artifacts (Betts 2008).

*\subsubsection*{Lake Site, Southampton Island, Nunavut}*

*Extract ID: KKHH-2:357*

The origin and development of Sadlermiut culture on Southampton Island, Nunavut, was the reason for excavations at the Lake Site (KkHh-2) by Brenda Clark in 1978 [(Clark 1980)](https://paperpile.com/c/GftuPk/SCk6l). Nine semi-subterranean Thule winter houses, with architectural similarities to those described for the northwestern Hudson Bay mainland [(Allen P. McCartney 1977)](https://paperpile.com/c/GftuPk/CoT6O), were identified along a beach ridge facing Native Bay; two of these structures were excavated (House 2 and House 5). Walrus and seal dominate the faunal remains, but a few dog bones were recovered, which included an adult mandible that was tested for mtDNA. Two original dates for the site based on caribou bone suggest an occupation between AD 1300 and 1600 (154). A caribou long-bone shaft fragment was submitted by C. Darwent for radiocarbon dating by Arizona AMS (AA102343: 374 ± 45 BP, d13C -17.9‰), which suggests the site most likely falls between AD 1450 and 1650 or Classic (middle) Thule.

*\subsubsection*{Learmonth, Creswell Bay, Somerset Island, Nunavut}*

*Extract ID: PEJR-1:H1-5201, PEJR-1:H5-M2, PEJR-1:H5-M3, PEJR-1:H5-M4*

Three Thule houses were excavated at the Learmonth site (PeJr-1) located on the north shore of Creswell Bay, Somerset Island, under the direction of Allen McCartney in 1976. Faunal remains from House 1 at this site, and House 6 at the Cape Garry site nearly 40 km to the south, were analyzed by Anne Rick [(Rick 1980)](https://paperpile.com/c/GftuPk/6eg5N). House 1 was a well-preserved semi-subterranean dwelling with two internal platforms, an entrance tunnel, and an external kitchen area. A humerus from House 1, and three left mandibles from House 5 were sampled at the Canadian Museum of History for mtDNA.

A caribou long bone shaft from House 1 and deciduous premolar from House 5 were submitted by C. Darwent for radiocarbon dating with Arizona AMS. House 1 produced a calibrated date of AD 1295–1420 (AA102353: 588 ± 45 BP, d13C -19.3‰), and House 5 a date of AD 900–1160 (AA102354: 1007 ± 46, d13C -20.9‰). The latter date is too early for initial Thule occupation of the Canadian Arctic, but the former fits well with other comparable early Thule sites in this region.

*\subsubsection*{McKinley Bay, Tuktoyaktuk Peninsula, Mackenzie Delta, Northwest Territories}*

*Extract ID: OATI-1:F2-M1, OATI-1:F2-M2, OATI-1:H1R, OATI-1:H1S*

The McKinley Bay site (OaTi1) is located on the northern margin of McKinley Bay on the Tuktoyaktuk coast, east of the Mackenzie Delta, Northwest Territories. At least 12 semi-subterranean houses and associated caches make up the site. In 1991, partial excavation of House 1 was undertaken by Chuck Arnold of the Prince of Wales Northern Heritage Centre; constructed of driftwood, this Thule house had a tunnel entering into a rectangular floor, but with no benches [(Matthew W. Betts 2008)](https://paperpile.com/c/GftuPk/nzphP). A scapula from Level 2 and a radius from Level 5 were sampled for mtDNA. Matthew Betts returned to the site in 2004 to undertake further testing in Area 1, Feature 2, and we sampled two right mandibles from Level 4 of this midden area. Radiocarbon dates and the associated artifact assemblage indicate site was occupied AD 1400–1650 (Betts 2008).

*\subsubsection*{Minto Lake Site, Minto Flats, middle Tanana Valley, Alaska}*

*Extract ID: UA64-95*

The Minto Lake Site is situated on a low grass-covered rise in Minto Flats and was investigated by John Matthews during the fall of 1964. Five test pits were excavated at the site and revealed concentrations of bone, hearth features, rolled pieces of birch bark, stone tools, flakes, worked bone and antler, and fire cracked rock. In test pit 2 a dog skull was collected approximately 4” below the ground surface in association with a rolled piece of birch bark. This dog specimen was sampled for this study.

*\subsubsection*{Misigtoq, Kangartik, Greenland}*

*Extract ID: TRF-01-08(ZMK 18/1932)*

Misigtoq is a Thule site located in Northeast Greenland in the along the Ammassalik Fjord [(Therkel Mathiassen 1933; Morey 2010)](https://paperpile.com/c/GftuPk/9NogJ+9nIm2). The site was identified and excavated in 1931 by Therkel Mathiassen during his investigation into the ‘Angmagssalik Eskimos’ of East Greenland to elucidate the direction of migration to East Greenland [(Therkel Mathiassen 1933)](https://paperpile.com/c/GftuPk/9NogJ). Settlement at Misigtoq has been suggested to have started as early as the 13th century CE and includes a total of nine house ruins [(Gullov 1997; Therkel Mathiassen 1933)](https://paperpile.com/c/GftuPk/lhkfj+9NogJ). Mandibles on site with holes drilled in the ascending ramus possibly for use as toy sledges [(Morey 2010)](https://paperpile.com/c/GftuPk/9nIm2). The sample obtained for this study from Misigtoq was taken from a petrous bone.

*\subsubsection*{Native Point, Southampton Island, Nunavut}*

*Sample ID: NMNH\_300707, NMNH\_300773, NMNH\_300774, NMNH\_300775, NMNH\_300776, NMNH\_300777, NMNH300772, NMNH302619*

Native Point (KkHh-1), is a Sadlermiut site on Southampton Island. The origins and relationship between Sadlermiut culture and other Inuit populations in the region remains ambiguous, with parts of their material cultural reflecting Dorset traits, and others more closely affiliated with the Thule [(Ryan and Young 2013)](https://paperpile.com/c/GftuPk/KCAIx). Based on the dating of the human remains found at the site, it is likely that the samples included here date to the proto-historic period, prior to sustained European contact in the 19th century CE, and ~150-350 years prior to their final demise in 1902-1903 due to a devastating disease epidemic [(Ross 1977)](https://paperpile.com/c/GftuPk/sPjVz). A total of eight individual specimens were analysed for GMM in this study, including eight lower M1s and six corresponding mandibles. These specimens are now housed at the Smithsonian National Museum of Natural History.

*\subsubsection*{Nelson River, Banks Island, Northwest Territories}*

*Extract ID: OHRH-1:1426M1, OHRH-1:1426M2*

The Nelson River site (OhRh-1) is situated on the southern coast of Banks Island, just west of the mouth of the Nelson River [(Max Friesen and Arnold 2008)](https://paperpile.com/c/GftuPk/fTOKa). The semi-subterranean house was made of driftwood timbers and planks; it includes two distinct rooms, a raised earth platform interpreted as a sleeping area, and a separate external kitchen alcove make up this complex dwelling structure [(Max Friesen and Arnold 2008)](https://paperpile.com/c/GftuPk/fTOKa). Several chronologically significant harpoon heads associated with the initial Thule migration into Canada were recovered, including Sicco, Natchuk, and Thule Type 2. Three new radiocarbon dates on muskox and caribou bone from this feature provide an age range of AD 1030‒1300 [(Max Friesen and Arnold 2008)](https://paperpile.com/c/GftuPk/fTOKa). Two lumbar vertebrae, one from a larger and one from a smaller variety of dog, were sampled for mtDNA at the Prince of Wales Northern Heritage Centre in Yellowknife.

*\subsubsection*{Nugarsuk, Upernavik District, Greenland}*

*Sample ID: ZMK\_P70*

*Extract ID: TRF-01-10(ZMK 139/1967-P69), TRF-01-11(ZMK 139/1967-P70)*

In 1966 Jørgen Meldgaard recommended the excavation of a Thule site in the Upernavik District Greenland [(Møhl 1979)](https://paperpile.com/c/GftuPk/dNABD). The site of Nugarsuk is located on an island 20 kilometers from Upernavik, archaeological excavations began in 1967 by Jan Hjarnø [(Møhl 1979)](https://paperpile.com/c/GftuPk/dNABD). The settlement was occupied between approximately 1650 and 1850 CE [(Møhl 1979)](https://paperpile.com/c/GftuPk/dNABD). Six houses, both single family and communal house, were excavated on the site and it appears that only one house occupied at a time [(Møhl 1979)](https://paperpile.com/c/GftuPk/dNABD). Analysis of the faunal remains revealed that 96\% of the remains belonged to, ring seals (*Phoca hispida*) [(Møhl 1979)](https://paperpile.com/c/GftuPk/dNABD). The abundance of ring seal remains indicates that the community was depended on ring seals for subsistence [(Møhl 1979)](https://paperpile.com/c/GftuPk/dNABD). The preservation on the site was excellent, with skin and flesh present on bones due to the permafrost conditions [(Møhl 1979)](https://paperpile.com/c/GftuPk/dNABD). 155 bones or bone fragments were attributed to dogs, two petrous bones were sampled for this project along with one crania for GMM analysis.

*\subsubsection*{Nulato, Yukon River, Alaska}*

*Sample ID: NMNH\_A08222*

The Nulato site is located on the lower Yukon River in the Yukon Valley, Alaska, and has been of interest to anthropologists and explorers due to its potential for sites dating to the early arrival of humans in the Americas. A number of sites in this region were found during surveys in the late 19th and early twentieth centuries, which were later ascribed to the Thule culture based on architectural design and artefact type [(de Laguna 1936)](https://paperpile.com/c/GftuPk/qLpAh). Glover Allen reports that one of the largest dogs he examined was collected by Dr. W. H. Dall at Nulato, Alaska ([(Allen 1920)](https://paperpile.com/c/GftuPk/0fGFw) pg 444), which is now housed at the Smithsonian National Museum of Natural History, and the mandible and associated lower M1 were included in this analysis.

*\subsubsection*{Nunalleq, Yukon-Kuskokwim Delta, Alaska}*

*Sample ID: NUN\_12001\_21, NUN\_12001\_35, NUN\_12003\_30, NUN\_12003\_47, NUN\_12003\_57, NUN\_12003\_58, NUN\_12003\_90\_C2, NUN\_12004\_90, NUN\_12005\_29, NUN\_12006\_16, NUN\_12006\_2\_IS, NUN\_12007\_91\_IS, NUN\_12010\_25\_C2, NUN\_12012\_3\_IS, NUN\_12012\_30, NUN\_12014\_27, NUN\_12014\_28, NUN\_12014\_39, NUN\_12014\_41, NUN\_12014\_6\_IS, NUN\_12014\_8\_C1, NUN\_12019\_82\_C1, NUN\_12019\_82\_C2, NUN\_13007\_72, NUN\_13015\_56, NUN\_13015\_57\_IS, NUN\_13016\_58, NUN\_13016\_59, NUN\_13030\_224, NUN\_13030\_227, NUN\_13041\_224, NUN\_13085\_228\_C2\_B, NUN\_13092\_20\_C1, NUN\_13092\_20\_C2A, NUN\_13092\_20\_C2B, NUN\_13102\_26\_IS, NUN\_13131\_223, NUN\_13137\_223, NUN\_13137\_224, NUN\_13140\_59, NUN\_13146\_229, NUN\_14021\_53, NUN\_14048\_89, NUN\_14065\_66\_C1, NUN\_14078\_68\_C1, NUN\_14080\_55, NUN\_14083\_91, NUN\_14087\_47, NUN\_14097\_20, NUN\_15160\_32\_C1, NUN\_3HF\_11, NUN\_3HF\_11\_C2, NUN\_3HF\_8\_IS, NUN\_3HF\_9, NUN\_3HF\_9\_IS, NUN\_4\_5, NUN\_5cHF\_90, NUN\_HF\_33*

*Extract ID: AL2784, AL2788, AL2791, AL2792, AL2794, AL2795, AL2797*

The Nunalleq site (GDN-248) is located in the Yukon-Kuskokwim Delta region of Southwestern Alaska, between the Kanektok and Arolik rivers, south of the modern Yup’ik village of Quinhagak. Nunalleq was a large prehistoric, semi-subterranean settlement site dating from predominantly from c. AD 1450-1700 [(Britton et al. 2013; Forbes, Britton, and Knecht 2015; Ledger et al. 2016)](https://paperpile.com/c/GftuPk/B31FT+gIedR+qo7mc), with the main occupation from AD 1570 - 1675 [(Ledger et al. 2018)](https://paperpile.com/c/GftuPk/TYBnC). It consists of multiple, multi-layer domestic structures with thousands of organic artefacts and ecofacts preserved by the discontinuous permafrost covering of the site [(Ledger et al. 2016)](https://paperpile.com/c/GftuPk/qo7mc), with dating further supported by the lack of any Euro-American artefacts, though a small amount of metal and metal modified artefacts have been found, consistent with trade for Eurasian metal prior to European contact [(H. K. Cooper et al. 2016)](https://paperpile.com/c/GftuPk/aEX8h). Dog remains were the second most abundant mammal species found at Nunalleq after pinnipeds [(E. T. McManus-Fry 2015)](https://paperpile.com/c/GftuPk/N6a4z), and their remains indicate a close relationship between humans and dogs at the site, based on a wide age range of dogs from the site from puppies to fully grown adults and their deposition within domestic spaces as well as middens [(E. McManus-Fry et al. 2016; Forbes, Britton, and Knecht 2015)](https://paperpile.com/c/GftuPk/FLte6+gIedR). For this analysis, 52 lower M1s, 21 mandibles and five crania were used from a total of 62 individual specimens.

*\subsubsection*{Nuvuk, Cape Krusenstern, Western Coronation Gulf, Northwest Territories}*

*Extract ID: NCPF-1:8-20*

The Nuvuk site (NcPf-1) is located at the base of the Cape Krusenstern peninsula, on the mainland coast of western Coronation Gulf. It was an area known for tom-cod fishing, sealing, and caribou hunting and is mentioned by Diamond Jenness in his book on the Copper Eskimo [(D. A. Morrison 1983)](https://paperpile.com/c/GftuPk/dtiX6). Houses 1 and 2 at the Nuvuk site were the remains of Thule winter houses with remnants of a poorly defined sleeping platform and an entrance tunnel [(D. A. Morrison 1983)](https://paperpile.com/c/GftuPk/dtiX6) . A dog mandible from this house was sampled for mtDNA. The site is estimated to have the same early Thule date as the nearby Clachan site at AD 1200–1400 [(D. A. Morrison 1983)](https://paperpile.com/c/GftuPk/dtiX6) .

*\subsubsection*{Nuvuk, Point Barrow, North Slope, Alaska}*

*Extract ID: UA2001-76-1*

The Nuvuk site (49-BAR-011) is a multicomponent site at Point Barrow in northern Alaska [(Jensen 2009)](https://paperpile.com/c/GftuPk/lXFas). The Thule occupation on Point Barrow, Alaska is known mostly from an occupation associated with a large Thule cemetery at Nuvuk [(Jensen 2009)](https://paperpile.com/c/GftuPk/lXFas). A single dog skull collected by Otto Geist at Point Barrow was sampled for DNA. No date is listed but this specimen was likely collected in the 1950s or 1960s. No other contextual information exists for the cranium, which is housed at the University of Alaska Museum of the North.

*\subsubsection*{Nyboe Land, Greenland}*

*Sample ID: ZMK-NYB-60-1985*

This Northwestern most portion of Greenland is relatively unexplored, as it remains difficult to access. However, there are four known archaeological sites in this region, two dating to the Independence I/II cultures, and two to the Thule period [(Gronnow and Jensen 2009)](https://paperpile.com/c/GftuPk/k6dN1). The Thule era site of Strømstedet is the only one which reports the recovery of any faunal remains, which are now held at the Danish Zoological Museum [(Gronnow and Jensen 2009)](https://paperpile.com/c/GftuPk/k6dN1). At this site, a sled shoe made of whale bone was also found. A single dog mandible was included in the GMM analysis for this study.

*\subsubsection*{Onion Portage, Kobuk River, Alaska}*

*Extract ID: OP-B1-M1, OP-B1-M2, OP-H13-M1, OP-H13-M2, OP-H13-M3, OP-H13-M4, OP-H13-M5, OP-H13-M6*

The Onion Portage site (49-AMR-170) is located on the northern edge of the middle Kobuk River in northern Alaska [(Anderson 1988)](https://paperpile.com/c/GftuPk/G72rU). Excavated in several stages, J.L. Giddings initiated small-scale testing in 1941, 1961, and 1963 followed by deep vertical trench excavations in 1964 to discern the depth of the cultural layers (82). Douglas Anderson took over direction of the excavation after Giddings’s death and expanded on his trenches to explore the horizontal extent of the site, along with testing of the surrounding area between 1966 and 1973 (82). Of the faunal remains recovered from the Onion Portage excavations, dogs were only identified from the Band 1 or Upper levels. This band spans the Arctic Woodland Culture from ca. AD 1000 to the 18^th^ century [(J. Louis Giddings 1952)](https://paperpile.com/c/GftuPk/RIzAU). Two dog mandibles are documented to have been recovered from Band 1, and six were recovered from within House 13, an Ahteut phase structure radiocarbon dated to ca. AD 1025–1225 (82). Faunal remains from this site are housed at the Haffenreffer Museum of Anthropology at Brown University.

*\subsubsection*{Peale Point, Frobisher Bay, Baffin Island, Nunavut}*

*Extract ID: KKDO-1:2270, KKDO-1:2282, KKDO-1:2363*

Excavations were undertaken at the Peale Point site (KkDo-1), a large Thule settlement located on a small island 13 km west of Iqaluit on southern Baffin Island, Nunavut, under the direction of Doug Stenton between 1981 and 1983 [(Stenton 1987)](https://paperpile.com/c/GftuPk/KEL0u). A total of five semi-subterranean sod houses were excavated that produced a faunal assemblage of over 28,000 specimens. Each house was constructed following what Stenton refers to as the basic Thule design: a paved cold-trap entrance, a main room with sleeping platform, and cooking/storage areas. Samples were taken early Thule components (AD 1200–1400) from three dog ulnae at the Canadian Museum of History for mtDNA testing. A caribou molar was submitted by C. Darwent for radiocarbon dating with Arizona AMS, but unfortunately it did not produce a date.

*\subsubsection*{Piġniq (Birnirk), North Slope}*

*Sample ID: NMNH\_241526*

*Extract ID: UA2012-51-9215, UA2012-51-3947*

Piġniq, the Birnirk type site, is located on the northern coast of Alaska at the intersection of the Chukchi and Beaufort Seas near Utqiaġvik [(J. L. Giddings 1960)](https://paperpile.com/c/GftuPk/UrmPd). The site consisted of twenty anthropogenic mounds representing approximately 1500 years of human occupation. In the early 1950s Wilbert Carter excavated in three of these mounds (H, Q, and L) mostly focusing on a large trench in Mound H. The trench excavated through Mound H included forty-seven 6’ x 6’ squares (~1.8 m^2^) and most of these were excavated to sterile gravels, although several areas did not thaw enough to find the bottom of the cultural layers [(Carter 1966)](https://paperpile.com/c/GftuPk/CKFka). Two dog skulls were sampled for this study and include one collected from the 5-5.5’ (~1.52-1.67 m) level in Mound H and another collected from the 3-3.5’ (~0.91-1.06 m) level in Mound L. Material used for GMM analysis from this site included a single crania, a mandible, and 2 lower M1s from collections held at the Smithsonian National Museum of Natural History.

*\subsubsection*{Pond Site, Richards Island, Mackenzie Delta, Northwest Territories}*

*Extract ID: NITS-2:H1B, NITS-2:H1H, NITS-2:H2MC1, NITS-2:H2MC2*

Located on Richards Island on the west bank of the East Channel, the Pond site (NiTs-2) is located about 1 km upriver from Kuukpak (NiTs-1) and adjacent to a small stream that flows into the Mackenzie River (Betts 2008). At least seven house ruins remain at the site, and during the summer of 1989 two houses and their associated midden areas were excavated under the direction of Chuck Arnold of the Prince of Wales Northern Heritage Centre [(Matthew W. Betts 2008)](https://paperpile.com/c/GftuPk/nzphP). House 1 at the Pond site was a semi-subterranean Thule-style dwelling with a rectangular main living area, a rear sleeping alcove, and a cold-trap entrance tunnel. House 2 appears to have had a similar configuration to House 1, but its architecture was not as well preserved at the rear. Both houses included extensive midden areas. From House 1 a dog baculum (with a healed midshaft fracture) and humerus were collected for DNA testing, and from House 2 an adult and a juvenile metacarpal. House 1 appears to be slightly older and dates to AD 1200–1400, whereas House 2 dates to AD 1300–1500 (Betts 2008); however, the ranges overlap considerably and the houses were likely reoccupied.

*\subsubsection*{Porden Point, Grinnell Peninsula, Devon Island, Nunavut}*

*Extract ID: RBJR-1:1042.27, RBJR-1:1042.47, RBJR-1:642.56, RBJR-4:222.21, RBJR-4:222.34, RBJR-4:336.19, RBJR-5:99.1A, RBJR-5:99.1B, RBJR-5:99.4*

Porden Point is located on the southeastern portion of the Grinnell Peninsula on northern Devon Island, Nunavut; it is made up of three sites (RbJr-1, RbJr-4, and RbJr-5) that include 17 Thule winter houses and over 100 other feature types that date to AD 1200 to 1400. Three houses were excavated by Robert McGhee in 1976 and 1977, and 13 additional houses were excavated by Robert Park in 1984 and 1985 [(Park 1987)](https://paperpile.com/c/GftuPk/uEuYq). Based on cranial and mandibular elements of dogs from these sites, Park estimates between 16 and 21 dogs are represented, many of which have evidence of traumatic injuries such as broken canines and healed depressed fractures [(Park 1987)](https://paperpile.com/c/GftuPk/uEuYq). We sampled for mtDNA across these cranial elements, which were boxed together at the Canadian Museum of History: three crania from RbJr-1, one cranium and two mandibles from RbJr-4, and two crania from RbJr-5.

*\subsubsection*{Point Spencer, Port Clarence, Norton Sound, Alaska}*

*Extract ID: UA2001-72-2*

A single dog skull and mandible were collected by an unknown individual at Point Spencer, Alaska and housed at the University of Alaska Museum of the North. No date is given for when these specimens were collected, but there is a remark in the primary documentation saying the “skull is recent prehistoric.” The skull from this collection was sampled for DNA but the mandible was not.

*\subsubsection*{Punuk, Punuk Islands, Alaska}*

*Sample ID: NMNH\_258795\_1, NMNH\_258795\_10, NMNH\_258795\_2, NMNH\_258795\_3, NMNH\_258795\_4, NMNH\_258795\_5, NMNH\_258795\_6, NMNH\_258795\_8, NMNH\_258795\_9, NMNH-258771-1, NMNH-258771-10, NMNH-258771-12, NMNH-258771-13, NMNH-258771-4, NMNH-258771-5, NMNH-258771-6, NMNH-258771-7, NMNH-258771-9, NMNH258771-11, NMNH258771-3, NMNH258795\_11, NMNH258795\_7*

The Punuk Site (49-XSL-006) is located on the northeasternmost of the three Punuk Islands, just off the southeastern end of St. Lawrence Island in the Bering Sea. It was originally excavated by Henry Collins in 1928,[(Hrdlicka 1930; W. W. Fitzhugh 2016)](https://paperpile.com/c/GftuPk/VqOUM+MMNn9) The precontact settlements on the island consist of houses and an extensive midden deposit, 16 feet deep [(Rainey 1941)](https://paperpile.com/c/GftuPk/jpSvK). The midden contained what was considered typical refuse for the period and region, including large quantities of sea mammal and bird bones, stone tools and remnants of organic materials. 18 dog mandibles, 17 M1s and three crania from 24 individual dogs found at Punuk Island were used in the GMM analysis.

*\subsubsection*{Qaqaitsut West, Paris Fjord, Inglefield Land, Greenland}*

*Extract ID: KNK492x36a (KNK492x33), KNK492x36b, KNK492x37, KNK492x38, KNK492x39, KNK494x1b, KNK494x2b, KNK494x3*

Excavation of four early Thule winter houses and an associated cache feature was undertaken at Qaqaitsut by the Inglefield Land Archaeology Project in 2009—two on the east side of a pond and two on the west. Four to five Inughuit families from Qaanaaq resided here in the mid-1980s, and thus the site was well-known to the Greenland National Museum before we undertook mapping of the extensive historic and prehistoric features [(LeMoine and Darwent 2010)](https://paperpile.com/c/GftuPk/ig5DN). Eroding into the west side of a pond were the remains of several Thule winter houses, one of these houses yielded an unusual concentration of dog remains in the kitchen area, most of which exhibited numerous cut marks and cracking for marrow extraction; however, few artifacts were recovered. The mandibles sampled for DNA represent seven adult dogs, and they were likely the remains of a sled-dog team consumed over a harsh winter. A fragment of a dog mandible from this house feature (KNK492) was refit with the rest of the element recovered more than 15 m away in a stone cache feature (KNK494); both features were used at the same time, and likely during a single winter. Radiocarbon dates on caribou bone recovered from the same house deposits date to AD 1275–1450 or the early Thule period [(Sarah K. Brown, Darwent, and Sacks 2013)](https://paperpile.com/c/GftuPk/61pmw).

*\subsubsection*{Qilalukan (Cockburn), Mittimatalik (Pond Inlet), Baffin Island, Nunavut}*

*Sample ID: ZMK\_TRF\_01\_34*

*Extract ID: TRF-01-34(ZMK23/2017), TRF-01-39(ZMK22/2017)*

At the time of excavation the Qilalukan site was located two kilometres from the Hudson’s Bay Company Station at Pond Inlet in northern Baffin Island [(Therkel Mathiassen 1927a)](https://paperpile.com/c/GftuPk/lGFLr). Excavations were undertaken by Therkel Mathiassen in 1923 as a part of the Fifth Thule Expedition, and again by Peter Freuchen in 1924 [(Therkel Mathiassen 1927a)](https://paperpile.com/c/GftuPk/lGFLr). The site is thought to have been an important narwhal (*Monodon monoceros*) hunting ground and as a result the site’s name means narwhal [(Therkel Mathiassen 1927a)](https://paperpile.com/c/GftuPk/lGFLr). Based on the similar typology in the archaeological assemblage, the decay of the ruins, and the uplift of land Mathiassen estimates this site like some of the other older Thule sites in the area could be up to 1000 years old [(Therkel Mathiassen 1927a)](https://paperpile.com/c/GftuPk/lGFLr). Ethnographic accounts relate that until the turn of the 20th century Qilalukan remained an important ceremonial and winter residence to local populations [(Therkel Mathiassen 1927a)](https://paperpile.com/c/GftuPk/lGFLr). Materials recovered from the site include many sledge remains such as sledge shoes, pegs, toggles, trace buckles, and crossbars [(Therkel Mathiassen 1927a)](https://paperpile.com/c/GftuPk/lGFLr). Six dog bones were identified from the site, two of these were sampled for the mitochondrial portion of this study which were derived from contexts with human graves, with one of the crania also used for GMM analysis.

*\subsubsection*{Rama, Ramah Bay, Labrador}*

*Sample ID: HMCZ\_7407*

The Thule arrived in northern Labrador, Canada around 700 - 600 BP, though the region surrounding Ramah Bay had been occupied by Palaeo-Eskimo groups since the Maritime Archaic period (7000 - 3500 BP). The region was known particularly for the highly prized and unique “Ramah chert”, which is found extensively along the north Atlantic coast and sourced from Ramah Bay [(Renouf 1993; Kaplan 1980)](https://paperpile.com/c/GftuPk/PYFRK+QhDzH), and there are a few Thule period sod houses from this region (Figure 2 in [(Natcher and Procter 2012)](https://paperpile.com/c/GftuPk/4haOA). A mandible and m1 from a single specimen, identified as an “Eskimo Dog” by the collector J.D. Sornborger, are including in the GMM analysis, and is currently held in collections at the Harvard Museum of Comparative Zoology.

*\subsubsection*{Resolute Bay, Cornwallis Island, Nunavut}*

*Sample ID: NMNH\_291088, NMNH\_291091*

The site at Resolute Bay, Cornwallis Island in the Canadian Arctic was first excavated by Henry Collins of the Smithsonian National Museum of Natural History in the late 1940s. The site consists of four semi-subterranean houses, and artefacts and the associated midden determined the site to be from the Thule period [(H. B. Collins 1951)](https://paperpile.com/c/GftuPk/jqEYQ). Many bones were also recovered, and included in this study are the remains of two dogs, with mandibles and lower M1s from both used in the GMM analysis.

*\subsubsection*{Saunaktuk, Eskimo Lakes, Mackenzie Delta, Northwest Territories}*

*Extract ID: NGTN-1:6802M, NGTN-1:6802S, NGTN-1:81002T*

Two sod and driftwood houses were identified at the Saunaktuk site (NgTn-2) on a peninsula jutting into the Eskimo Lakes, east of the Mackenzie Delta, Northwest Territories [(Matthew W. Betts 2008)](https://paperpile.com/c/GftuPk/nzphP). Excavations in Feature 1, 1 m high mound that had been disturbed by bulldozer activity, were undertaken in the late 1980s by Chuck Arnold (Betts 2008). The floor of the house was paved with flat stones, and the former structure appeared to be of an A-frame rather than typical four-post style of typical Thule houses in the west. From the undisturbed cultural layers, three specimens were sampled from Feature 1: a juvenile mandible and an adult scapula from one unit, and an adult incisor from another unit separated by two meters. Typical Mackenzie Inuit-style artifacts included net fishing gear that post-dates AD 1400, along with radiocarbon dates, suggest an AD 1400–1500 occupation.

*\subsubsection*{Silumiut Island, Northwestern Hudson Bay, Nunavut}*

*Extract ID: KKJG-1:H3-T1, KKJG-1:H4-S1, KKJG-1:H8-M1, KKJG-1:H13-M1, KKJG-1:H14-M1, KKJG-1:H14-M2, KKJG-1:H14-M3*

Silumiut Island is one of a number of small islands that dot the coast of northwestern Hudson Bay, and at low tide is connected to the mainland. The Silumiut site (KkJg-1) was excavated under the direction of Allen McCartney between 1967 and 1969 [(Allen P. McCartney 1977)](https://paperpile.com/c/GftuPk/CoT6O). A total of 28 Thule-style, stone-and-sod, semi-subterranean dwellings with cold-trap entrance tunnels were recorded, and seven of these houses were excavated (House 3, 4, 5, 6, 8, 13, 14). A massive quantity of faunal remains was recovered from this site, which is now housed in boxes at the Canadian Museum of History. We searched through 50 boxes of faunal remains [(Staab 1979)](https://paperpile.com/c/GftuPk/PhHnt); four of these contained *Canis* bone for mtDNA sampling. House 3 midden, one adult tibia; House 4, one adult scapula; House 8, one adult mandible; House 13 midden, one adult mandible; House 14 midden, one juvenile and two adult mandibles. Only a single driftwood sample had been run originally (GAK-2759, 690 $\pm$ 90 BP), and therefore C. Darwent selected associated caribou bone, where possible, for radiocarbon dating (Table S5). Dates were obtained on four of the houses sampled and these span the period from AD 1400 to 1650. The site is about 150–200 years younger than had been estimated originally [(Allen P. McCartney 1977)](https://paperpile.com/c/GftuPk/CoT6O) and is not associated with early Thule, but rather with middle or Classic Thule.

*\subsubsection*{Singauruk, Lopp Lagoon, Seward Peninsula, Alaska}*

*Extract ID:SI-H2-1, SI-H2-2*

Approximately 40 km north of the coastal village of Wales, Alaska, and situated between Lopp Lagoon and the Bering Sea, is an old channel known locally as Singauruk. Giddings first identified this location in 1959, and undertook excavations of part of a single house floor [(J. Giddings 1973)](https://paperpile.com/c/GftuPk/rgXZA). Two Canis skeletal elements (adult metatarsal, old adult mandible) were submitted for mtDNA testing. This occupation dates to early Thule based on the presence of a Thule Type 2 harpoon head and the results of two radiocarbon samples submitted by C. Darwent on caribou bone from the faunal assemblage: 1) 942 ± 63 BP, d13C -17.3‰ (AA102341); 2) 880 ± 30 BP; d13C -18.8‰ (Beta-301397). Together these dates suggest an age of AD 1000‒1200 for the Singauruk samples.

*\subsubsection*{Skraeling Island, Buchanan Bay, Ellesmere Island, Nunavut}*

*Extract ID: SFFK-4:H10-H1, SFFK-4:H11-M1, SFFK-4:H11-M2, SFFK-4:H17-F1, SFFK-4:H20-H1, SFFK-4:H21-M1, SFFK-4:H22-T3, SFFK-4:H2-T2, SFFK-4:H3-F1, SFFK-4:H4-H1, SFFK-4:H4M-H1, SFFK-4:H6-H1, SFFK-4:H8-M1*

Sites in the Bache Peninsula region were investigated by members of the Arctic Institute’s Ellesmere Island Research Project under the direction of Peter Schledermann between 1977 and 1982 [(McCullough 1989)](https://paperpile.com/c/GftuPk/7D2og). The Skraeling Island site (SfFk-4) is located on the southern tip of Skraeling Island in Buchanan Bay, and adjacent to Kane Basin on the east coast of Ellesmere Island, Nunavut. In total, the site consists of 23 early Thule (AD 1200–1400), Ruin Island-style winter houses [(McCullough 1989)](https://paperpile.com/c/GftuPk/7D2og). The massive faunal and artifact assemblage from these structures were analyzed by Karen McCullough. Most of the dog (or wolf) remains were postcranial elements; however, at least eight complete skulls were identified as dog based on their morphology. In addition, McCullough notes the recovery of sled-dog traction gear and dog whips, and thus emphasizing the importance of dog-sled teams to the early Thule occupants ([(McCullough 1989)](https://paperpile.com/c/GftuPk/7D2og):268–269).

Houses 1–4 are clustered together; House 4 is a large square-shaped structure identified as a “festival house” or *qargi*. A total of five dog remains were sampled for mtDNA across three houses: a tibia was sampled from the kitchen floor of House 2, a proximal femur from House 3, and two left humerii (one juvenile and one adult) from House 4 and the associated midden area. A single humerus was sampled from House 6, which was isolated at the north end of the site. House 8 is another *qargi*, and houses 9 and 10 are associated dwelling structures, both of which have distinct kitchen alcoves. From House 8, a mandibular tooth was sampled, and from House 10 a humerus. Teeth from two adult mandibles were sampled from House 11—a large, triple-roomed structure with a kitchen alcove. A single house ruin located on a relatively steep slope was designated as House 17; a distal femur was sampled. House 20 is isolated above the cluster of houses 8, 9, and 10, and a humerus was selected for sampling. Houses 21 and 22 were clustered together and also included distinct kitchen alcoves; a mandibular tooth was sampled from the former and a tibia from the latter. All of the faunal remains from this site are stored at the Canadian Museum of History (with the exception of midden materials from House 4 at the University of Calgary).

*\subsubsection*{Stormbugt I, Northeast Greenland}*

*Extract IDs: TRF-01-01(ZMK 13w/1909-P60), TRF-01-02(ZMK 13w/1909-P61)*

The site of Stormbugt I is lies at 76°46’N in northeastern Greenland on the Stormbugt Bay. Samples were collected by Bendix Thostrup in 1907 during the Danmarks Expedition for ethnographic research [(Thostrup 1911)](https://paperpile.com/c/GftuPk/Qljqu). The site consists of tent rings, winter houses, traps, and house stores, and four human graves. The dog remains that were collected during the expedition are presumed to be of Thule origin [(Thostrup 1911)](https://paperpile.com/c/GftuPk/Qljqu). DNA was extracted from two dog petrous bones from the Danmarks Expedition materials from Stormbugt I, inventoried in 1909, which are stored by the Natural History Museum of Denmark.

*\subsubsection*{Sukersit, Ammassalik District, East Greenland}*

*Extract ID: TRF-01-03(ZMK 23/1932)*

In the Ammassalik District, Sukersit represents one of the oldest and largest villages in the region [(Therkel Mathiassen 1933)](https://paperpile.com/c/GftuPk/9NogJ). The site consists of nine house ruins, seven human graves, four tent rings, and several other structures [(Therkel Mathiassen 1933)](https://paperpile.com/c/GftuPk/9NogJ). Dog bones were found within several houses, in addition to fragments of human remains [(Therkel Mathiassen 1933)](https://paperpile.com/c/GftuPk/9NogJ). On the basis of house type Mathiassen suggests that the site witnessed two separate occupations [(Therkel Mathiassen 1933)](https://paperpile.com/c/GftuPk/9NogJ). The earliest of these occupations taking place around the end of the 14th century CE[(Therkel Mathiassen 1933)](https://paperpile.com/c/GftuPk/9NogJ). At the time of excavation in 1931, Mathiassen estimates that it had been at least 200 years since the more recent occupation of the site [(Therkel Mathiassen 1933)](https://paperpile.com/c/GftuPk/9NogJ). One petrous bone was sampled from Sukersit and extracted for this study.

*\subsubsection*{Talaguak, Kimmirut (Lake Harbour), Baffin Island, Nunavut}*

*Extract ID: KEDQ-2:M1, KEDQ-2:P1*

Excavations were undertaken at the Talaguak site (KeDq-2) near the village of Kimmirut (Lake Harbour) along Hudson Strait on southern Baffin Island under the direction of M.S. Maxwell and George Sabo III of Michigan State University in 1977‒78 [(Sabo and Jacobs 1980)](https://paperpile.com/c/GftuPk/H34Pm). Five Thule-style houses were excavated at this site (House 2, 5, 7, 9, 11); a juvenile mandible and an adult pelvis from the Lower Component of House 2 were sampled for mtDNA at the Canadian Museum of History. This house component was identified as early Thule (AD 1200‒1300) based on the presence of, among other items, Thule Type 2 harpoon heads, and the architectural style of the house (Sabo and Jacobs 1980).

*\subsubsection*{Tikilik, Igloolik Island, Nunavut}*

*Extract ID: NIHF-4:132C, NIHF-4:1C, NIHF-4:4PLM, NIHF-4:TDL*

The Tikilik site (NiHf-4) is located on a peninsula jutting out along the southwestern coast of Igloolik Island and was excavated by Susan Rowley in 1993–94. Faunal remains from the Paleo-Inuit components of this site were analyzed (Murray 1996); however, none of the bone from the early Thule components had been identified or previously published. Using notes associated with the original excavation bags and unpublished documents at the Prince of Wales Northern Heritage Centre in Yellowknife, four *Canis* specimens were collected from the Thule (AD 1200–1400) upper peat layer components for mtDNA sampling: a palate/alveolus from Unit 13N/2E (NIHF-4:132C), a palate/alveolus from the floor of House 1 (NIHF-4:1C), a mandible from Feature 4 (Unit -1N/-3E; NIHF-4:4PLM), and a lumbar from Test Unit D (NIHF-4:TDL).

*\subsubsection*{Tiktalik, south Amundsen Gulf, Nunavut}*

*Extract ID: NKRI-3:616M, NKRI-3:623M*

Tiktalik (NkRi-3) is comprised of the remains of at least five early Thule winter houses on the southern coast of Amundsen Gulf, Northwest Territories. Excavation of House 5, the largest of the semi-subterranean house ruins, was undertaken by David Morrison in 1998; faunal remains from this site were analyzed by John Moody [(Moody and Hodgetts 2013)](https://paperpile.com/c/GftuPk/Wfvug) and are stored at the Prince of Wales Northern Heritage Centre in Yellowknife. Two adult, right mandibles recovered from Level 7 of the oil-soaked “Kitchen” were sampled for mtDNA. Radiocarbon dates on caribou bone submitted by Morrison as well as the abundance of Sicco-style harpoon heads all point to this being an early Thule house dating to AD 1200‒1300 (Moody and Hodgetts 2013). An additional caribou incisor from House 5 at the Canadian Museum of History was submitted by C. Darwent for radiocarbon dating with Arizona AMS (AA102350: 684 ± 49 BP, d13C -18.1‰), which produced a calibrated age of AD 1255‒1400.

*\subsubsection*{Uivak Point 1, Okak Bay, Labrador}*

*Extract ID: HJCL-9:01, HJCL-9:03, HJCL-9:04, HJCL-9:05, HJCL-9:07, HJCL-9:09, HJCL-9:10, HJCL-9:13, HJCL-9:14, HJCL-9:18, HJCL-9:20, HJCL-9:25, HJCL-9:27*

The Uivak Point 1 site is located in the Okak Bay region of Labrador. According to Kaplan ([(Kaplan 2012)](https://paperpile.com/c/GftuPk/lrnwD):28), “Uivaq was one of three eighteenth-century Inuit whaling settlements in Okak”, dating to the late 1700s. A large communal Inuit sod house (House 7) along with an associated midden was partially excavated Susan Kaplan and James Wollett in 1993 and 1994. This house is estimated to have been an 18 x 12 meter structure that included an entrance passage, cold trap, paved house floor, and raised sleeping platform that ran the entire length of the back wall of the house. Moravian accounts and European manufactured materials indicate the house was most likely occupied in the late eighteenth century [(Kaplan and Woollett 2000; Kaplan 2012)](https://paperpile.com/c/GftuPk/47lcT+lrnwD). Over 52,000 animal bones were recovered and analyzed from the House 7 midden alone [(Woollett 2003, 2007)](https://paperpile.com/c/GftuPk/hqbK2+7eaAW), which includes hundreds of dog remains. All samples for this study were recovered from various levels of the midden area adjacent to House 7, with the exception of HJCL-9:03, which is from a nearby test unit (Test Pit 5).

*\subsubsection*{Utkiavik, Utqiaġvik, North Slope, Alaska}*

*Sample ID: NMNH258810*

Utkiavik (49-BAR-002) is an Inupiaq site in Utqiaġvik (Barrow), Alaska. The area was first surveyed by the Smithsonian, led by James A. Ford in the 1930s [(Ford 1959)](https://paperpile.com/c/GftuPk/7c5go). The site consists of various anthropogenic mounds, which have been excavated as recently as the 1980s [(Sheehan 1997)](https://paperpile.com/c/GftuPk/mlOMD). For this analysis, the specimen from Utkiavik consists of a single lower M1 used for GMM analysis brought to the Smithsonian after Ford’s 1931 expedition to Alaska.

### *\subsection*{*Siberian Archaeological Sample Descriptions}

*\subsubsection*{Ainu Creek 1, Urup Island, Kurils}*

*Extract ID: KBP2569.045, KBP2569.170, KBP2569.179, KBP2569.283*

The Ainu Creek 1 site is located on the south end of Urup Island, on the Sea of Okhotsk. The site consists of an unknown number of house pits at an elevation ~10 m asl [(Ben Fitzhugh et al. 2016; B. Fitzhugh et al. 2007; {Fitzhugh et al. 2009)](https://paperpile.com/c/GftuPk/K1aGf+X8BB0+3DP2q). The midden deposit was tested by members of the Kuril Biocomplexity Project (KBP) in 2006 and 2007, and has materials dating to both the Epi-Jomon period and the Okhotsk period. Although the site has been heavily impacted by twentieth century military activities, the dog bones (two metapodials; scapula and maxillary fragments) sampled for mtDNA are derived from undisturbed Epi-Jomon deposits from Unit B2, Level 3, dated to ca. 780-230 BC [(Ben Fitzhugh et al. 2016)](https://paperpile.com/c/GftuPk/K1aGf).

*\subsubsection*{Boisman II, Primorsky}*

*Sample IDs: BOIS \#1, BOIS \#2, BOIS \#3, BOIS \#5, BOIS \#9, BOIS \#10*

*Extract IDs: AL2988, AL2989, AL2990, AL2991, AL2994, AL2995*

Boisman II is located on Boisman Bay to the southwest of Vladivostok on the east coast of the Russian Federation in the Primorksii region. The site complex consists of several mounds of shell midden and multiple human burials from the Siberian Neolithic Boisman culture [(A. N. Popov, Chikisheva, and Shpakova 1997)](https://paperpile.com/c/GftuPk/RJ9NW). The term Neolithic here primarily refers to the advent of the widespread use of pottery by forager populations, not agriculture [(Alexander N. Popov, Tabarev, and Mikishin 2014)](https://paperpile.com/c/GftuPk/nr4Rl). The site has been repeatedly radiocarbon dated, which suggests a maximum period of occupation between ~8500 and 5000 cal. BP [(Alexander N. Popov, Tabarev, and Mikishin 2014)](https://paperpile.com/c/GftuPk/nr4Rl). All of the dog remains at the site were found as isolated elements within midden sediments. Four of the six Boisman II dog remains were directly radiocarbon dated for this project (Table S5). Given the heavily reliance at the site on marine fish, mammals, and shellfish, the radiocarbon dates likely carry some marine reservoir bias, perhaps up to a few centuries.

*\subsubsection*{Bukhta Zakat, Makanrushi Island, Kurils}*

*Extract ID: KBP3316.216, KBP3316.228, KBP3316.229*

The Bukhta Zakat site is located on Bukhta Zakat (transl. “Sunset Bay”) on the southwest end of Makanrushi Island. The site consists of 17 house pits situated on flat grassy plain [(B. Fitzhugh et al. 2007)](https://paperpile.com/c/GftuPk/X8BB0). One test pit was excavated by KBP in 2008, exposing thick midden deposits dominated by cod bones dating to the Okhotsk period (ca. AD 1030-1190). An acetabulum, scapula blade fragment, and 5th metacarpal of a dog were all sampled for mtDNA.

*\subsubsection*{Cherniatino II, Primorsky}*

*Sample ID: CHER \#14*

*Extract ID: AL2997*

Cherniatino II is located ~80 km inland from Vladivostok near the village of Cherniatino in the Primorsky region of the Russian Federation. The site was occupied during the Bronze Age, Early Iron Age, and Medieval period. The dog sample analyzed for this study is from the Medieval Mokhe cultural deposits at the site, which contained remnants of dwellings, household waste pits, and faunal remains from wild and domestic species [(Nikitin, Iu.G., Chzhun, S., Chzho, Li Ch. 2008; Nikitin and Chzhun 2009)](https://paperpile.com/c/GftuPk/JXTS1+e4nUI). The Mokhe were settled agricultural peoples. The dog specimen was directly dated for this study to 1294 $\pm$ 33 (Ua-51216), or 1290 to 1180 cal. BP.

*\subsubsection*{Ekven Cemetery, Chukchi Peninsula, Chukotka}
Extract ID: 14.EC95.G329B.518, 15.EC95.G329B.651*

The Ekven site is located near Cape Dezhnev on the Chukchi Peninsula that is renowned for its cemetery, which has produced exquisite ivory artifacts and other grave goods belonging to the Old Bering Sea culture. Numerous investigations have been undertaken at the site [(Arutiunov, S. A., & Sergeev, D. A. 1990; Bronshtein and Dneprovsky 2002; Dneprovsky and Bronshtein 2002; Csonka 2014)](https://paperpile.com/c/GftuPk/8srrG+6OBfn+PKYIJ+5YFch). In 1995, two dogs were found interred together, both intentionally placed in a human grave (Grave 359) after the initially buried individual’s body was removed. Two grave goods (a walrus baculum and polar bear mandible) were present as well, with one of the dogs being an older male exhibiting cut marks on its occipital. A mandible fragment from one dog and a maxillary fragment from the other were submitted for DNA testing. One of the two dogs was radiocarbon dated to 1660$\pm$ $\pm$120 BP; however, the mean $\delta$13С value for the specimen was -13.62, which indicates that the dog had a predominantly a marine-based diet. Therefore, using a 100\% marine-diet calibration curve [(Stuiver, Reimer, and Reimer 2019)](https://paperpile.com/c/GftuPk/4QrBn), the sample dates to AD 483–996, which is more aligned with Punuk, but still pre-Thule or Paleo-Inuit.

*\subsubsection*{Kaniskak, Chukchi Peninsula, Chukotka}*

*Extract ID: 11.KG90.H1.658, 12.KG90.H1.747, 13.KG90.H1.708, 17.KM90.2/11.204, 18.KM90.2/11.971, 19.KM90.2/11.937, 20.KM90.2/11.1700, 21.KM90.2/11.938, 22.KM90.2/12.908, 23.KM90.2/12.907, 24.KM90.2/12.168, 25.KM90.2/10.922, 26.KM90.2/10.923, 27.KM90.2/9.931, 28.KM90.2/8.933, 29.KM90.2/7.1578, 32.KM90.2/4.266, 33.KM90.1/4.532, 34.KM89.1/3.247, 35.KM90.2/5.873, 36.KM90.2/5.272, 37.KM89.1/6.1566*

Kaniskak is a multicomponent village site located on a bluff overlooking the coast near Cape Dezhnev on the Chukchi Peninsula, Chukotka, Russia[(Gusev, Zagoroulko, and Porotov 1999)](https://paperpile.com/c/GftuPk/1wdkG). Investigations at the site were undertaken between 1989 and 1991. The site has a residential area with five semi-subterranean houses and an accompanying large stratified kitchen midden. The houses are generally associated with the Old Bering Sea and Thule cultures; however, the midden deposits span the entire sequence from 100 BC to AD 1800.

*Paleo-Inuit samples:* In House 1, the remains of two adult *Canis* ulnae were recovered from excavation of Level 4 and Level 5 and submitted for DNA testing (Extract ID: 12, 13); these date to AD 300–800 or the Old Bering Sea culture[(Gusev, Zagoroulko, and Porotov 1999)](https://paperpile.com/c/GftuPk/1wdkG). A total of 19 adult *Canis* specimens were submitted from levels 3 through 12 of the stratified kitchen midden deposits. Three left mandible fragments from Level 12 (Extract ID: 22–24), and five maxillary fragments from Level 11 (Extract ID: 17–21) date from 100 BC to AD 400 or early Old Bering Sea levels[(Gusev, Zagoroulko, and Porotov 1999)](https://paperpile.com/c/GftuPk/1wdkG). Two tibia from Level 10 (Extract ID: 25–26), an ulna from Level 9 (Extract ID: 27), two mandibles from Level 7 and Level 8 (Extract ID: 28–29), and a maxillary fragment from Level 6 (Extract ID: 37) date to AD 400–500 or late Old Bering Sea. Two dog atlas fragments were submitted from Level 5 (Extract ID: 35–36), and three maxillary fragments from Level 3 and Level 4 (Extract ID: 33–34); all five of these samples are from Birnirk levels dating to AD 900–1350[(Gusev, Zagoroulko, and Porotov 1999)](https://paperpile.com/c/GftuPk/1wdkG).

*Inuit samples:* From House 1, one adult *Canis* ulna recovered from Level 2 (Extract ID: 11) dates to AD 1650–1850 or the late Thule period (214). The absence of historical artifacts suggests occupation was from earlier in the date range. Two mandibles were submitted for DNA testing from Level 2, or the Thule level of the midden dated to AD 1100–1500 (214).

*\subsubsection*{Paipelghak, Chukchi Peninsula, Chukotka}*

*Extract ID: 01.P09.B1.1077, 02.P04.H1.128, 03.P04.H1.1024, 04.P04.H1.151, 05.P04.H1.1612, 06.P05.H2.200, 07.P05.H2.959, 08.P05.H2.961, 09.P05.H2.894, 10.P05.AD5.895*

Paipelghak is a multicomponent village site located on the shore of the Chukchi Sea 90 km north of Cape Dezhnev on the Chukchi Peninsula[(Dneprovsky 2006)](https://paperpile.com/c/GftuPk/OwLxM). Investigations at the site began in 2002 and continued until 2010 under the direction of Kurill Dneprovsky. The excavated sections of the site contained a complex of five dwellings constructed of driftwood, stone slabs, and whale bones, with accompanying midden deposits[(Dneprovsky 2006)](https://paperpile.com/c/GftuPk/OwLxM). It appears that there was a regular cycle of reusing components of old dwellings in newer structures,which has made the stratigraphy of the site highly complex. Based on radiocarbon dates and temporally sensitive artifacts, it appears the occupation of Paipelghak spanned from 300 BC to AD 1850[(Dneprovsky 2006)](https://paperpile.com/c/GftuPk/OwLxM).

*Paleo-Inuit samples:* Four adult, left mandibles from Level 11 of House 1 (Extract ID: 2–5), and three tibiae from levels 7 and 8 of House 2 (Extract ID: 6–8) were submitted for DNA testing; these deposits date to AD 700–1250 or the Birnirk culture. Burial 1 represents the remains of a Birnirk dog that appears to have been a ritually buried with several grave goods [(Dneprovsky 2006)](https://paperpile.com/c/GftuPk/OwLxM). It was so well-preserved that hide and fur were still intact; a P3 and P4 were submitted for testing (Extract ID: 1).

*Inuit samples:* Two adult, right tibiae were recovered from Area D, Level 5 (Extract ID: 9–10), which dates to AD 1600–1850. The absence of historical artifacts suggests it was likely occupied earlier in this date range.

*\subsubsection*{Pospelova 1, Primorsky}*

*Sample ID: POS \#24*

*Extract ID: AL2992*

Pospelova 1 is located on Russki Island within the city of Vladivostok on the east coast of the Russian Federation in the Primorksii region. It consists of a shell midden and a ritual platform with a human burial. The excavated materials from Pospelova 1 are from the Early Iron Age Iankov culture, thought to date from ~2800 to 2000 BP [(Rakov et al. 2009)](https://paperpile.com/c/GftuPk/de440), or ~2950 to 1970 cal. BP. The dog sample analyzed here was not dated, but remains of six other dogs at the site are directly dated to ~3020 and 2750 cal. BP. Given the use of marine resources at the site, the radiocarbon dates likely carry some marine reservoir bias, perhaps up to a few centuries.

*\subsubsection*{Rasshua 1, Rasshua Island, Kurils}*

*Extract ID: KBP3674.045, KBP3695.003, KBP3700.074*

The Rasshua 1 site is located on the southwest end of Rasshua Island, on the Sea of Okhotsk. The site is situated on a low terrace, and consists of over 100 pit features, many or all of them likely to be prehistoric house pits [(B. Fitzhugh et al. 2007; Ben Fitzhugh et al. 2016)](https://paperpile.com/c/GftuPk/X8BB0+K1aGf). Two test pits were excavated by KBP in 2008, exposing thick midden deposits dating to the Okhotsk period (AD 980-1215). The dog bones (calcaneus, 4th metatarsal, femur) were recovered from Test Pits 1 and 1A and from levels 3 and 4.

*\subsubsection*{Russkii 1, Primorsky}*

*Sample ID: RUS \#2*

*Extract ID: AL2998*

Russkii 1 is located on Russki Island within the city of Vladivostok on the east coast of the Russian Federation in the Primorksii region. This shell midden site was sampled in 2010-11 and all archaeological remains recovered are from the Early Iron Age Iankov culture, thought to date from ~2800 to 2000 BP [(A. N. Popov and Lazin 2011)](https://paperpile.com/c/GftuPk/NoHk2), ~2950 to 1970 cal. BP. The midden contained remains of both wild and domestic fauna (pigs and cattle), as do other sites in the surrounding area from this general time period [(Kuzmin 1997)](https://paperpile.com/c/GftuPk/B23BZ). The single dog specimen analyzed here was not radiocarbon dated, but six others from the site are directly dated, with ages ranging from ~3200 to 2750 cal. BP[(A. N. Popov and Lazin 2011)](https://paperpile.com/c/GftuPk/NoHk2). Given the use of marine resources at the site, the radiocarbon dates likely carry some marine reservoir bias, perhaps up to a few centuries.

*\subsubsection*{Sagan Nuge III, Lake Baikal}*

*Sample ID: 2012-003b*

*Extract ID: AL2645*

Sagan-Nuge III is located on the Little Sea shoreline of Lake Baikal in southern Siberia northeast of the city of Irkutsk. This site consists of deflated stone constructions that were visible on the modern surface that were created during the Iron age [(T. I. Nomokonova and Goriunova 2013)](https://paperpile.com/c/GftuPk/6FlcP). The constructions contained ceramics, iron tools, and other artifacts, along with remains of possible offerings of domestic (horse, cattle, sheep/goat, dog) and wild (Baikal seal, fox, hare, fish) animals. The dog sample analyzed here was directly dated for this project to 1057 $\pm$ 31(Ua-44173), which calibrates to 1054 to 926 cal. BP. Organisms consuming fauna from Lake Baikal carry varying levels of freshwater reservoir effects [(T. Nomokonova et al. 2013)](https://paperpile.com/c/GftuPk/j6BzW). The stable isotope values for this dog ($\delta$13C = -17.2; $\delta$15N = 10.7) suggest its diet consisted of some lake foods and that the date is ~152 radiocarbon years too old [(Schulting et al. 2014)](https://paperpile.com/c/GftuPk/e7klX). Note that an earlier radiocarbon date (Beta-335122; 550 $\pm$ 30) on ungulate bone from a directly adjacent feature at the site suggests the site was used somewhat later, between 639 and 517 cal. BP [(T. I. Nomokonova and Goriunova 2013)](https://paperpile.com/c/GftuPk/6FlcP).

*\subsubsection*{Verbljuzhka, Chukotka}*

*Extract ID: 16.V89.C9.239*

Excavations were undertaken at Verbljuzhka in the Chukotka region in 1989. The fragment of an adult *Canis* mandible recovered from Cut #9 at 110–120 cm below surface and dating to AD 400–700 was submitted for DNA testing.

### *\subsection*{*Icelandic and Norse Greenlandic Archaeological Sample Descriptions}

*\subsubsection*{Alþingisreitur, Reykjavík}*

The Alþingisreitur site in downtown Reykjavík, Iceland was excavated in 2008-2009 and 2012 in advance of the construction of a new office building for the Icelandic Parliament [(Garðarsdóttir 2010, 2011, 2013)](https://paperpile.com/c/GftuPk/kRX4X+fTAgk+d8rdS). During the excavation large quantities of animal bones spanning the entire settlement period of Iceland from the late 9^th^ century until the 20^th^ century were recovered [(Pálsdóttir 2010, 2013)](https://paperpile.com/c/GftuPk/rPiFF+L4PAb). Over 21 000 bone fragments were recovered at the site but the analysis of the animal bones from the site has not been completed [(Pálsdóttir 2010, 2013)](https://paperpile.com/c/GftuPk/rPiFF+L4PAb). All known dog bones from the site have been recorded in detail, a total of 315 elements were identified as coming from dogs or dog/arctic fox and 15 as coming from arctic fox. Dog bones were often found partially articulated at the site so the MNI of dogs from the site is significantly lower but that analysis has not been completed [(Pálsdóttir 2010, 2013)](https://paperpile.com/c/GftuPk/rPiFF+L4PAb). Organic preservation at the site was in general excellent and a large number of textile fragments, wood and leather were found during the excavation [(Garðarsdóttir 2010)](https://paperpile.com/c/GftuPk/kRX4X). This good organic preservation can probably be traced to the partial waterlogging of the site which lies on what was a narrow stretch of land between the Reykjavík pond and the coastline. The Alþingisreitur site is divided into four main phases based on tephrochronology and radiocarbon dating but fine scale dating of the site using dateable artefacts has not taken place [(Garðarsdóttir 2010, 2011, 2013)](https://paperpile.com/c/GftuPk/kRX4X+fTAgk+d8rdS). Ancient DNA analysis has been done on three walrus bones from Phases III and IV from the site. One of the walrus bones had no DNA and two of the bones had extremely low endogenous DNA content [(Pálsdóttir, Boessenkool, and Star 2018)](https://paperpile.com/c/GftuPk/4rNQM). Many of the bones from the Alþingisreitur site have blue mineral deposits, most likely vivianite, which forms in iron rich soil where there is plenty of water. It has been suggested that vivianite can cause problems for DNA extraction [(McGowan and Prangnell 2006)](https://paperpile.com/c/GftuPk/nUM32) and indeed that does seem to be the case at Alþingisreitur where most aDNA analysis of animal bones has failed.

###

*\subsubsection*{Alþingisreitur, Reykjavík Modern}*

*Extract ID: 2008-32-1343*

Phase I of Alþingisreitur spans the time period from 1800 to the late 20^th^ century. The archaeological remains in this phase were very poorly preserved due to construction, cellars and various utilities in the area. Remains of concrete walls and cellars as well as stone walls were found along with disturbed peat ash refuse layers. The total number of bones from this phase is only 567 and the NISP 347. In general bone preservation from this phase was poor due to the amount of building activity during this site. Bones from this phase rarely had signs of vivianite deposits. The species found in this Phase I include sheep/goat, horse, cattle, dog, cat, pig, seals, whales, rat, chicken, auks, ptarmigan and gadid fish [(Pálsdóttir 2010)](https://paperpile.com/c/GftuPk/rPiFF). A total of 13 dog bones are recorded from this phase of the site [(Pálsdóttir 2010)](https://paperpile.com/c/GftuPk/rPiFF) .

###

*\subsubsection*{Alþingisreitur, Reykjavík Historical}*

*Extract ID: 2008-32-1616, 2008-32-621, 2009-32-1539, 2009-32-1629, 2009-32-1637, 2009-32-1760, 2009-32-1819, 2009-32-1838, 2009-32-3316*

Phase II spans between 1550-1800 CE. A number of building remains belong to this phase, including a smithy from The New Enterprises (Innréttingarnar) but there was also a 350 m^2^ refuse dump at the site during Phase II. The midden was mainly used for household refuse and large quantities of animal bones accumulated in the area. The total number of bones analysed from this phase is 7514 (TNF) with a NISP of 6043. The species found in this Phase II include sheep/goat, horse, cattle, dog, cat, pig, arctic fox, seals, whales, gulls, auks, ptarmigan, eider ducks, swan, goose and gadid fish [(Pálsdóttir 2010)](https://paperpile.com/c/GftuPk/rPiFF). A total of 145 dog or dog/arctic fox bones are recorded from this phase of the site [(Pálsdóttir 2010)](https://paperpile.com/c/GftuPk/rPiFF).

The one dog bone from Alþingisreitur that had any endogenous dog DNA was a complete skull, no. 2009-32-3316, from an adult animal that came from Phase II, context 358141. The context which the skull was found in was a peat ash waste deposit with wood fragments and a number of animal bones. The deposit is a result of a dumping event and part of a sequence of midden deposits covering the area. A cluster of five stones were placed in a circle at the southwest end of context 358141. The purpose for these stones is not clear, but the stones might have been acting as a stone packing to hold a post upright ([(Garðarsdóttir 2010)](https://paperpile.com/c/GftuPk/kRX4X), p.227). The context most likely dates to after 1600 since it included two clay pipes (Ragnheiður Traustadóttir, pers. comm 14.01.2019)

*\subsubsection*{Alþingisreitur, Reykjavík Medieval*}

*Extract ID: 2008-32-1184, 2008-32-1199, 2008-32-1208, 2008-32-2327, 2008-32-38, 2008-32-670, 2008-32-908, 2009-32-1556, 2009-32-1936, 2009-32-1948, 2009-32-2985*

Phase III 1223-1550 was also largely a refuse area with large quantities of well-preserved animal bones [(Garðarsdóttir 2010)](https://paperpile.com/c/GftuPk/kRX4X). The total number of bones from Phase III is 6399 (TNF) and the NISP is 3828. The species found in this Phase III include sheep/goat, horse, cattle, dog, cat, pig, seals, walrus, whale, arctic fox, gulls, auks, eider duck, goose and gadid fish [(Pálsdóttir 2010)](https://paperpile.com/c/GftuPk/rPiFF). A total of 145 dog or dog/arctic fox bones are recorded from this phase of the site [(Pálsdóttir 2010)](https://paperpile.com/c/GftuPk/rPiFF).

###

*\subsubsection*{Alþingisreitur, Reykjavík Norse/Viking}*

*Extract ID: 2008-32-1185, 2008-32-1221, 2008-32-1253, 2008-32-1276, 2008-32-1321, 2008-32-843, 2009-32-3111, 2012-32-4144*

Phase IV spans from the early settlement period of Iceland around 870 to 1223 CE. During this time period the site was a general activity area where ironworking and processing took place [(Garðarsdóttir 2010, 2011, 2013)](https://paperpile.com/c/GftuPk/kRX4X+fTAgk+d8rdS). The total number of bones from Phase IV is 1037 (TNF) and the NISP is 552. The species found in this Phase IV include sheep/goat, horse, cattle, dog, cat, pig, seals, walruses, whales, great auk, gulls, auks, eider duck, eagle, falcon and gadid fish [(Pálsdóttir 2010)](https://paperpile.com/c/GftuPk/rPiFF). A total of 10 dog or dog/arctic fox bones are recorded from this Phase IV of the site [(Pálsdóttir 2010)](https://paperpile.com/c/GftuPk/rPiFF).

*\subsubsection*{Bessastaðir, Álftanes Medieval}*

*Extract ID: 1988-213-374*

The Bessastaðir site is located on the low lying Álftanes peninsula in Southwest Iceland. It has been inhabited since the settlement period in the late 9^th^ century CE and is currently the residence of the president of Iceland. The Bessastaðir project was the first large scale rescue excavations in Iceland and took place from 1987 to 1996 during extensive renovation of the standing buildings at the site. Those buildings largely date to the mid-18^th^ century and were constructed to serve as the residency for the Danish governor of Iceland [(Ólafsson 2010)](https://paperpile.com/c/GftuPk/3gxAn). The archaeological site was much larger than initially anticipated and by 1996 4600 m^2^ had been excavated of the estimated 11 000 m^2^. The site stratigraphy was complicated with the many phases of construction from the 9^th^ century until the mid-18^th^ century as well as the organization of the renovations and little experience in Iceland with such a complicated large scale site [(Amorosi et al. 1992; Ólafsson 2010, 2013)](https://paperpile.com/c/GftuPk/oXybd+3gxAn+Suvit). Over 16 000 bone fragments from the Late Medieval (1450-1500 CE) and Early Modern (1600-1849 CE) phases have been analyzed from Bessastaðir identifying cattle, sheep/goats, horses, dogs, and wild species [(Amorosi 1996; Amorosi et al. 1992)](https://paperpile.com/c/GftuPk/tCeif+oXybd). The number of recorded dog bones from the site is 11 but the actual number of dog bones is likely much higher ([(Amorosi 1996)](https://paperpile.com/c/GftuPk/tCeif) p. 173 and unpublished data from AHP). Bones from the oldest phase of the site have not been analyzed and the size of the total size of the archaeofauna from Bessastaðir is unknown [(Amorosi 1996; Amorosi et al. 1992; Ólafsson 2013, 2010)](https://paperpile.com/c/GftuPk/tCeif+oXybd+Suvit+3gxAn). The publication of site reports fromm Bessastaðir by the National Museum of Iceland is still underway but is set to be completed in 2019 [(Ólafsson 2010)](https://paperpile.com/c/GftuPk/3gxAn).

*\subsubsection*{Garđar, Igaliku, Eastern Settlement, Greenland}*

*Extract ID: E47-DOG-36, E47-DOG-505-4*

Garđar was the seat of the Bishop in Norse Greenland (AD 1124–1378). The closest city to Igaliku is Qaqortoq in South Greenland. Archaeological excavations of these Medieval ruins were undertaken as early as the 19^th^ century; however, more recent systematic fieldwork at Igaliku was directed by Orri Vésteinsson in 2012 [(Vésteinsson 2014)](https://paperpile.com/c/GftuPk/yPtNj). From context “036” in the charcoal horizon of the Area A excavation block, a dog tibia and an astragalus were submitted by faunal analyst Konrad Smiarowski for DNA testing. Radiocarbon dates from the charcoal horizon suggest an occupation from the late 13^th^ to early 15^th^ century (Vésteinsson 2014).

*\subsubsection*{Gásir, Eyjafjörður, Iceland}*

*Extract ID: GÁS-DOG-101, GÁS-DOG-2349, GÁS-DOG-2812, GÁS-DOG-2851, GÁS-DOG-655*

Gásir remains date to the 14^th^ century cultural contexts connected to a trading post. All materials result from the 2001-2006 archaeological investigations by the Institute of Archaeology, Iceland [(Roberts 2009)](https://paperpile.com/c/GftuPk/hgdsk). The site is located on a coastal inlet of Eyjafjörður, NE Iceland, about 11 km north of Akureyri and 3 km northeast of the Möðruvellir farm estate. The Gásir archaeofaunal and artifact assemblage shows aspects of high status consumption of food and drink, via imported pottery used for wine or beer, and exchange, for example gyrfalcon bones, a sulfur processing pit, homespun sheep wool of varied quality used for likely both packing materials and export goods, as well as roving (unfinished strings of wool) were recovered from Gásir. Furthermore, there were large slabs of imported schist for whetstone making that might have originally functioned as ballast [(Harrison 2009, 2014)](https://paperpile.com/c/GftuPk/icj5O+uH4SD). Dog upper limb bones and a mandible from the Medieval Trading site, Area A, were tested for DNA.

###

*\subsubsection*{Hofstaðir, Mývatnssveit, Iceland}*

*Extract ID: HST-DOG-5169*

Hofstađir is located in the Mývatn Lake Basin area of northern Iceland, and a midden deposit located just outside the churchyard of the Medieval Christian cemetery (AD 1280–1320) was excavated in 2011 [(McGovern, Smiarowski, and Harrison 2013)](https://paperpile.com/c/GftuPk/Jx3zt). Dog bones are generally rare in Icelandic archaeofauna; however, at least four dogs of various ages were recovered in the same deposit (McGovern et al. 2013). A tibia fragment from an adult dog was submitted for DNA testing.

*\subsubsection*{Oddsstaðir, Vesturland, Iceland}*

*Extract ID: ODÖ-DOG-108*

The Oddstaðir farm ruins are located on relatively flat, south facing pasture land at c. 150-160m asl, north of the river Hörgá and circa 20 km SW of Gásir. Materials discussed here are from the midden excavations as part of the Gásir Hinterlands Project in 2009. The Oddstaðir midden deposits provide a chronology of activities spanning several centuries, from the farm’s likely establishment in the late 9^th^ or early 10^th^ century, through to farm abandonment in the late 14^th^ century, around the same time when Gásir exchange ended. The archaeofauna suggests that Oddstaðir could have been an independent farm, at least at the time of its Settlement in the late 9^th^ to early 10^th^ century [(Harrison 2011, 2014)](https://paperpile.com/c/GftuPk/3ubRL+uH4SD). One dog vertebra was sampled for DNA.

###

*\subsubsection*{Skútustaðir, Mývatnssveit, Iceland}*

*Extract ID: SKU-DOG-1, SKU-DOG-2*

Skútustaðir is a farm site located in Mývatnssveit, northern Iceland. Excavations undertaken in the 2011 field season involved expansion of the large Trench H midden [(Hicks et al. 2013)](https://paperpile.com/c/GftuPk/BAhOS). A mandibular and a maxillary deciduous premolar of a juvenile dog were submitted for DNA testing from the early Modern deposits (1600–1700) from this midden.

*\subsubsection*{Tasiliqulooq, Aniaaq Fjord, Eastern Settlement}*

*Extract ID: E171-DOG-11*

A dog humerus from Tasiliqulooq (E171), a Norse farm settlement in Aniaaq Fjord in the Qaqortoq municipality of south Greenland, was submitted for DNA testing.

*\subsubsection*{Tatsip Ataa, Igaliku Fjord, Eastern Settlement}*

*Extract ID: E172-DOG-03, E172-DOG-07, E172-DOG-12*

The Norse farm site of Tatsip Ataa (E172) is located on the eastern shore of Igaliku Fjord in the Qaqortoq municipality of south Greenland. Excavation of a Norse midden area was undertaken under the direction of Konrad Smiarowski in 2009–10 [(Smiarowski 2012)](https://paperpile.com/c/GftuPk/ji8X8). Part of the midden was in use from around AD 1000 to 1300. A sacrum of a small-sized dog (DOG-07) and a sacrum (DOG-03) and tibia (DOG-12) of a large-sized dog from the midden area were submitted for DNA testing.

### *\subsection*{*Museum and Ethnographic Sample Descriptions}

*\subsubsection*{Natural History Museum of Denmark}*

*CN2587, CN3294, CN2040, CN2041*

Four tanned historical museum hides were sampled for this study. Two of the samples originate from East Greenland, Scoresbysund in 1928 and Angmagssalik in 1934, respectively. Two additional specimens from The Fifth Thule Expedition 1921-24, collected in eastern Canada, one in winter 1922/23 in Iglulik and one in 1923 on “Danske Øen”.

*\subsubsection*{National Museum of Denmark}*

Extract IDs: *TRF-02-14(P.32.6), TRF-02-16(P.1659), TRF-02-17(Lu.798a), TRF-02-18(Lu.798b), TRF-02-19(Lu.788), TRF-02-20(Lu.788), TRF-02-21(K.1-1a), TRF-02-27(P.6402), TRF-02-28(P.6396), TRF-02-29(P.6397), TRF-02-30(P.6399), TRF-02-31(K.6411a), TRF-02-32(P26.2), TRF-02-33(P.229a), TRF-02-34(P.231a), TRF-02-35(P.1358), TRF-02-36(Lu.930), TRF-02-37(L.5083), TRF-02-38(L19.3), TRF-02-39(L.2106), TRF-02-40(Lu.979), TRF-02-41(L.18.194), TRF-02-42(Lu.805), TRF-02-43(Lu.804), TRF-02-44(Lu.808), TRF-02-45(Lu.800), TRF-02-46(Ld.8a), TRF-02-47(L.9357), TRF-02-63(Kd.13), TRF-02-66(L.2103), TRF-02-67(K.3-13), TRF-02-68(P.229e), 31369(Ld.103), 31588\_a(L.2097), 31588\_b(L.2097), 32179(L.5082), 30491(Lc.186c), 31867(L.4364), 32135(L.5039), 32987(L.9344), 35561(L19.4a-b), 67941(L.7827)*

A total of 42 specimens were sampled from ethnographic collections housed at the National Museum of Denmark in Copenhagen. Three samples derived from Siberian clothing (Table S1 for specimen sampled from garments). The remaining 39 samples derive from Inuit clothing in Greenland, Canada, and Alaska. These samples were taken from fur clothing which was collected on ethnographic expeditions or donated to the National Museum. Macroscopic analysis was done to identify the types of fur used to make the garments, which includes headgear, parkas, trousers, footwear, or mittens. The samples included in this study from these materials originated from the American Arctic and was identified to belong to either dogs or wolves.

*\subsubsection*{Greenland National Museum and Archives}*

*Extract IDs: TRF-01-04, TRF-01-05, TRF-01-06, TRF-01-07, TRF-01-30, TRF-01-31, TRF-01-35, TRF-01-36, TRF-01-41, TRF-01-42, TRF-01-43, TRF-01-46, TRF-01-49, TRF-01-52, TRF-01-53, TRF-01-54, TRF-01-55, TRF-01-56, TRF-01-57, TRF-01-58, TRF-01-59, 19451(K.613), 19619(K.907), 19741(K.1162), 36960(P32.3), 37351(P30.9), 37351\_a(P30.9), 67733(Kc.108a), 89375(K.1-3), 89594(K.2-11), 90710(Kc.99), 91085(Kd.10), 36635(P.1658), 37233(P31.1), 66272(Ic.191), 70111(Pd.2a)*

A total of 36 historical and ethnographic samples from the Greenland National Museum and Archives, housed at the Natural History Museum of Denmark were also included in this study. These samples were collected on various expeditions to Greenland, some in the form of surface finds and others were collected from living individuals during these expeditions.

*\subsubsection*{Natural History Museum, University of Oslo, Norway}*

*Extract ID: GL1(NHMO-DMA-55366/1-P), GL2(NHMO-DMA-55369/1-P)*

Two Greenland dog hides from dogs used under Amundsen's South Pole expedition 1911-1913, the dogs are supposedly of Greenland origin.

*\subsubsection*{Museum of Cultural History, University of Oslo, Norway}*

*Sample ID: Sort, Grå*

Two stuffed dogs brought to Norway with Amundsen after his exploration of the Northwest Passage (1903–1906). The dogs supposedly origin from the modern day Gjoa Haven area.

*\subsubsection*{The Ski Museum, Oslo, Norway}*

*Sample ID: Obersten*

A famous lead sled dog used under Amundsen's South Pole expedition 1911-1913, it is recorded the dog originated from Greenland.

*\subsubsection*{Zoological Institute of the Russian Academy of Sciences}*

*Sample ID: StP\_2848, StP\_3437, StP\_5811, StP\_20033, StP\_20039, StP\_9290, StP\_9292, StP\_9299, StP\_14732, StP-6122,*

*Extract ID: AL3004, AL3007:3437, AL3023:5811, AL3031:20033, AL3032:20039, AL3052:9290, AL3053:9292, AL3054:9299, AL3080:14732*

Nine specimens were sampled for this study from the ethnographic collections at the Zoological Institute of the Russian Academy of Sciences. During the expedition of Russian scientist N.A. Grebnickii in 1884 specimens were collected from Native Kamchatkan dogs (2848, 3437) and Native sled dogs from the Bering Island's (9290, 9292, 9299). A Native Chukotka dog specimen (14732) collected during the expedition of Russian scientist K. Chapskii in 1928. During the expedition of Russian scientist N. Gondatti in 1896 a Native Chukotka dog specimen (5811) was collected. Native Chukotka dogs (20033, 20039) were also collected during the expedition of Russian scientist Dr. Grinevickii in 1891.

*\subsubsection*{American Museum of Natural History, New York}*

*Sample ID: AMHN\_30475, AMHN\_30476, AMHN\_30477, AMHN\_441\_21934, AMHN\_67191, AMHN\_67212, AMHN\_67229, AMHN\_67245, AMHN\_67179, AMHN\_67187, AMHN\_67190, AMHN\_67196, AMHN\_67203, AMHN\_67204, AMHN\_67207, AMHN\_67220, AMHN\_67227, AMHN\_67228, AMHN\_67230, AMHN\_67233, AMHN\_67235, AMHN\_67243, AMHN\_67253, AMHN\_67168, AMHN\_67182, AMHN\_67192, AMHN\_67194, AMHN\_67195, AMHN\_67198, AMHN\_67201, AMHN\_67210, AMHN\_67213, AMHN\_67216, AMHN\_70955, AMHN\_1907\_155, AMHN\_67199, AMHN\_67215, AMHN\_67223, AMHN\_67232, AMHN\_67251, AMHN\_30442, AMHN\_30450, AMHN\_67169, AMHN\_67197, AMHN\_67211, AMHN\_67214, AMHN\_67222, AMHN\_67224, AMHN\_70942, AMHN\_70943, AMHN\_70944, AMHN\_70945, AMHN\_70946, AMHN\_70947, AMHN\_70948, AMHN\_70949, AMHN\_70950, AMHN\_70952, AMHN\_70953, AMHN\_70954, AMHN\_70956, AMHN\_70958, AMHN\_30439, AMHN\_30440, AMHN\_30441, AMHN\_30474, AMHN\_30478, AMHN\_30482, AMHN\_67202, AMHN\_67205, AMHN\_67208, AMHN\_67219, AMHN\_67234, AMHN\_67240, AMHN\_67242, AMHN\_67248, AMHN\_67255, AMHN\_67256, AMHN\_67239, AMHN\_67244*

Pleistocene North American wolf specimens from the Alaska area held at the American Museum of Natural History were used for GMM analysis, including 64 lower M1s, 35 mandibles and 1 crania from 81 individual specimens.

*\subsubsection*{American Museum of Natural History, New York}*

*Extract ID: M-14045, M-14445*

Robert E. Peary’s Expeditions to the North Pole resulted in a multitude of natural history specimens collected for the American Museum of Natural History in New York. Included in this collection are more than a dozen skulls and complete skeletons of dogs used by Peary’s expedition as sled dogs. A mixture of male and female adult and old adult dogs many of which had healed and unhealed cranial wounds were collected by Peary’s team in 1896 and 1897. Teeth from two adult males were sampled for mtDNA.

*\subsubsection*{University of Alaska Museum of the North}*

*Hooper Bay Village*

*Extract ID: UA1-1950-1789, UA1-1950-2093, UA1-1950-2094, UA1-1950-2110, UA1-1950-2111, UA1-1950-2112, UA1-1950-2113*

The village of Hooper Bay is located on the Bering Sea coast in southwest Alaska on the north side of the bay. The oldest existing portion of the still-occupied village is situated along the banks of a slough where Wendell Oswalt [(Oswalt 1952)](https://paperpile.com/c/GftuPk/z1v7k) noted evidence of prehistoric houses and a midden, and where he chose to focus archaeological excavations during the summer of of 1951. Contemporary buildings covered much of the prehistoric site and therefore Oswalt excavated two blocks, one on either end of the landform. These excavations were slowed by permafrost and by the end of the field season one block reached a depth of 1.5 meters and the other a depth of 2.5 meters, and the base of the cultural deposit was not found in either. Based on tree-ring dates from three spruce driftwood samples recovered during excavation, Oswalt hypothesized a date of A.D. 1600 for the lowest levels of the midden excavation with shallower levels dating to the late A.D. 1800s. Seven complete dog craniums from this site were sampled for DNA and all of them were collected from the surface and likely date to the late Historic or Modern time periods.

*\subsubsection*{Point Hope, Old Village}*

*Extract ID: UA5072-1, UA5072-2, UA5072-3, UA5072-5, UA5072-7*

Twelve dog specimens (7 skulls and 5 mandibles) were collected by Otto Geist at Point Hope in 1961. The primary documentation that exists for this collection indicates these remains were collected from the old village site excavation, which may refer to archaeological work conducted during airfield construction at the village in 1961. Five of the seven skulls included in this collection from the University of Alaska Museum of the North were sampled for mtDNA and likely date to the late Historic or Modern period.

*\subsubsection*{School of Anthropology, University of Arizona}*

*Anaktuvuk Pass, Alaska*

*Extract ID: U.Arizona-1, U.Arizona-2, U.Arizona-3, U.Arizona-4, U.Arizona-5, U.Arizona-6*

A total of six upper second molar samples were submitted from six separate crania collected by Lewis Binford [(Binford 1978)](https://paperpile.com/c/GftuPk/8BiOA) during his Nunamiut Ethnoarchaeological research in the Anaktuvuk Pass region of northern Alaska between 1969 and 1972. These crania, which are housed at the University of Arizona, were surface collected on the tundra from unknown contexts, and thus were somewhat weathered and many of the teeth had been lost post-mortem; several individuals display joint trauma of the upper neck and/or occipital region. It is estimated that the animals died sometime in the mid-20^th^ century. Based on the physiological conditions of the crania, which includes broken or worn teeth, some or all of the individuals appear to have been used as sled dogs by local Nunamiut hunters.

*\subsubsection*{Modern Greenland Sledge Dog Samples}*

*Extract ID: GSD\_19.5, GSD\_2.6, GSD\_51602, GSD\_51603, GSD\_GS16, GSD\_GS31, GSD\_Mums, GSD\_Pondus, GSD\_Q11, GSD\_QSON*

10 modern samples from tissue or DNA extract biobanks at Greenland Institute of Natural Resources, Arctic Research Centre - Aarhus University or Department of Veterinary and Animal Sciences - University of Copenhagen. There are 2 samples from each of 5 modern Greenland cities or villages, specifically, Qaanaaq, Ilulissat, Aasiaat, Sisimiut and Tasiilaq.

*\subsubsection*{Bern Natural History Museum}*

*Sample ID: NMBE\_1051369, NMBE\_1051371, NMBE\_1051372, NMBE\_1051373, NMBE\_1051377, NMBE\_1051386, NMBE\_1051393, NMBE\_1055699, NMBE\_1055702, NMBE\_1055705, NMBE\_1058274, NMBE\_1058384, NMBE\_1059182, NMBE\_1059211*

Modern breed Greenland Dogs were collected for GMM analysis from the collections at the Natural History Museum, Bern Switzerland. These specimens all come from local Greenland dog breeders based in Switzerland between 1972–2004.

*\subsubsection*{National Museum of Natural History, Washington DC}*

*Sample ID: NMNH\_147705, NMNH\_257445, NMNH\_181244, NMNH\_203895, NMNH\_175669, NMNH\_175670, NMNH\_175671, NMNH\_282817, NMNH\_282818, NMNH\_282819, NMNH\_283586, NMNH\_291012, NMNH\_507338, NMNH\_242704, NMNH\_A06256, NMNH\_A09000, NMNH\_A09001, NMNH\_A09003, NMNH-134131, NMNH\_134781, NMNH-008710-A07154, NMNH\_243573, NMNH\_243574, NMNH\_243576, NMNH\_243577, NMNH\_212609, NMNH-257446, NMNH\_147470, NMNH\_147472, NMNH\_168440, NMNH\_127550, NMNH\_130897, NMNH\_180282, NMNH\_180283, NMNH\_302628, NMNH\_302629, NMNH\_243324, NMNH\_243325, NMNH\_A23137, NMNH\_A23140, NMNH-A23138, NMNHA23138, NMNH\_134496, NMNH\_134497, NMNH\_274485, NMNH\_274486, NMNH\_146361, NMNH\_212610, NMNH\_214477, NMNH\_214480, NMNH\_214481, NMNH\_214482, NMNH\_A16750, NMNH\_291008, NMNH\_291010, NMNH-291009, NMNH\_177370, NMNH177370, NMNH\_243327, NMNH\_243329, NMNH\_243329, NMNH\_244206, NMNH\_244207*

Modern wolf specimens from the collections at the Smithsonian National Museum of Natural

History used for GMM analysis.

*\subsubsection*{British Natural History Museum, London}*

*Sample ID: BNHM\_1979\_5444*

Ethnographic sample from Baffin Island, Nunavut

*\subsubsection*{Qaanaaq, Thule District , Greenland}*

*Sample ID: NMNH\_275071*

Qaanaaq, also called Thule or New Thule is a modern village in Northwestern Greenland. The specimen used in this study is a mandible for GMM analysis, and was collected by A.J. Duvall and C.O. Handley during an expedition sponsored by the Smithsonian in 1946.

*\subsubsection*{Harvard Museum of Comparative Zoology}*

*Sample ID: HMCZ\_B\_9070, HMCZ\_7409, HMCZ\_7410, HMCZ\_7422, HMCZ\_7482, HMCZ\_7411*

Modern wolf specimens from the collections at the Harvard museum of comparative zoology used for GMM analysis.

*\subsubsection*{Naturalis Biodiversity Center Leiden}*

*Sample ID: SNRM\_595129, SNRM\_582376*

Modern wolf specimen from Naturalis Biodiversity Center, Leiden.

*\subsubsection*{University of Alaska Museum of the North}*

*Cape Dyer, North Slope*

*Extract ID: UA5231*

A single dog skull was collected by William Pruitt on July 20, 1960 from the ground surface at Cape Dyer, east of the Kipalog Creek mouth north of Pt. Hope, Alaska. No other contextual information exists for this specimen.

*\subsubsection*{Cape Nome, Extinct Village, Alaska}*

*Extract ID: UA2001-74-1, UA2001-74-2, UA2001-74-3*

Three dog skulls were collected by Otto Geist at Cape Nome in 1946. Primary documentation notes the specific locality as “Extinct Eskimo village,” but no other contextual details are provided. All three of these specimens were sampled for mtDNA. The remains likely date to the late Historical or Modern period.

*\subsubsection*{Deering, Kotzebue Sound, Alaska}*

*Extract ID: UA2001-73-1, UA2001-73-2, UA2001-73-3*

Three dog skulls, one associated with a disarticulated mandible, were collected by an unknown individual at an unknown point in time (although likely between the 1930s and 1960s). Primary documentation states that these remains were collected at Deering, but no other contextual information exists for this mtDNA sample. The remains likely date to the late Historical or Modern period.

*\subsubsection*{Meade River, North Slope, Alaska}*

*Extract ID: UA4667, UA4667-1*

In 1959 and 1960 Otto Geist collected six dog skulls, one dog mandible, and one dog femur from the Meade River, North Slope, Alaska. The primary documentation for these specimens does not provide any additional contextual information. Two of these dog skulls were sampled for DNA. The remains likely date to the late Historical or Modern period.

*\subsubsection*{Oliktok Point, Lower Colville River, Alaska}*

*Extract ID: UA1-1952-1159, UA1-1952-1160*

Two dog mandibles were collected by William Irving during archaeological reconnaissance of the lower Colville River and the Colville Delta in 1952 [(Irving 1953)](https://paperpile.com/c/GftuPk/zxgYz). Specific details about the context of these two specimens is lacking, but they are likely from historic or protohistoric camps near Oliktok Point. Both mandibles were sample for DNA.

*\subsubsection*{Point Barrow, North Slope, Alaska}*

*Extract ID: UA5225*

A single dog skull was collected by Otto Geist from Point Barrow in 1960 and is likely modern. Primary documentation for this specimen lists “Birnirk excavation” in parenthesis as the specific location, but no other contextual information exists; it was sampled for mtDNA. The cranium could date from Birnirk through the Thule/Historic periods.

*\subsubsection*{Point Spencer, Port Clarence, Norton Sound, Alaska}*

*Extract ID: UA2001-72-2*

A single dog skull and mandible were collected by an unknown individual at Point Spencer, Alaska. No date is given for when these specimens were collected, but there is a remark in the primary documentation saying the “skull is recent prehistoric.” The skull from this collection was sampled for DNA but the mandible was not.

*\subsubsection*{Wales, Seward Peninsula, Alaska}*

*Extract ID: UA594-M-5824*

A single dog skull and unarticulated mandible was collected in Wales, Alaska by Charles Lucier prior to December of 1952, which is the date it was accessioned. Little information exists for this specimen sampled for mtDNA, but it was likely collected from the surface and dates to the historic period.

*\subsubsection*{Wainright Lagoon, North Slope, Alaska}*

*Extract ID: UA1-1959-proj247*

A single dog mandible collected by Otto Geist at Wainwright Lagoon in 1959 was sample for this project. No other contextual information exists for this specimen sampled for mtDNA, but it was likely collected from the surface and represents the historic time period.

*\subsubsection*{Walakpa, North Slope, Alaska}*

*Extract ID: WAL-B10-J16, WAL-B10-J19A, WAL-B10-J19B, WAL-B10-J19C, WAL-B10-J19D, WAL-B10-J19E, WAL-B10-J20, WAL-B10-K17, WAL-B10-K18, WAL-B10-K19, WAL-B9-J19, WAL-B9-J20, WAL-B9-K15, WAL-B9-K19*

The Walakpa site is located near the modern village of Utqiaġvik (Barrow), in the North Slope region of northern Alaska. Excavation of this multi-component site was undertaken by Dennis Stanford in 1968 and 1969 (Stanford 1976) and revealed historic, late Thule, early Thule, Birnirk, and some possible ASTt deposits. Birnirk deposits were superposed in levels 8, 9, and 10. Radiocarbon dating from this site and typological cross-dating with other dated Birnirk sites in the area indicate the Birnirk occupation here was from AD 650–1000. Although dominated by small seals, and to a lesser extent caribou, a total of ts 296 Canis remains were recovered from across all Thule and Birnirk levels of the site; nearly 70\% of these were recovered from levels 9 and 10 of Area B. We concentrated our sample search in these lower Birnirk levels, plus dog crania from these levels were boxed together. Faunal remains from excavations at Walakpa are stored at the Maxwell Museum at the University of New Mexico. Tooth roots from of 14 different crania/mandibles, some of which still had dried or burned tissue adhering, were sampled for mtDNA from Area B, levels 8–9, units J-16, J-19, J-20, K-15, K-17, K-18, K-19.

*\subsubsection*{University of California, Davis Zooarchaeology Laboratory}*

*Inglefield Land, Northwest Greenland*

*Extract ID: UCDZL-1080, UCDZL-1081, UCDZL-1082*

Three dog crania were collected by C. Darwent during the Inglefield Land Archaeology Project in 2009. Two were surface collected adjacent to houses originally built at Innuarfissuaq, Marshall Bay, by Inughuit assistants to Danish archaeologist Eric Holtved in the 1930s; however, the houses continued to be reused by hunters in the region until the 1990s. The third cranium was collected at Qaqaitsut, Paris Fjord, and likely dates to the mid-1980s when four to five Inughuit families, on the initiative of the community of Qaanaaq, built houses and endeavored to “live on the land” [(LeMoine and Darwent 2010)](https://paperpile.com/c/GftuPk/ig5DN) .

## SI Figure Captions

**Fig. S1:** MapDamage plots showing general patterns of deamination seen in samples included in the study with transition frequency plotted against position in read. Deaminated bases appear most frequently at the ends of reads, the frequency of the appearance of deaminated bases increases between younger and older samples. A) Plot of historical sample collected ca. 135 years before present. B) Plot of Thule Inuit sample, ca. 600 years old. C) Plot of Siberian Holocene sample, ca. 6,000 years old.

**Fig. S2**: Maximum likelihood tree generated with RAXML with 1,000 bootstraps from samples with a minimum mean of 10x read depth of 95\% of sites in the mitochondrial genome with the exclusion of samples in basal positions of the subclades to ensure robusticity of node support.

**Fig. S3:** Bayesian skyline plot of whole dataset, plot displates Ne against time (years before present). The black line represents the average Ne and the 95\% HPD is depicted with the purple area. Bayesian skyline plot of all samples with a minimum mean of 10x read depth, generated with BEAST2.4.

**Fig. S4:** Maximum likelihood tree generated with RAXML with 1,000 bootstraps from samples with a minimum mean of 3x read depth of 80\% of positions in the mitochondrial genome. Arrows indicating A-clade subclades and X-clade. Three specimen (coyote outgroup and two Chinese wolves) were excluded from figure for better visualisation, remaining samples were coloured according to cultural affiliation. Dog clades B-E are collapsed into black clades with labels in red.

**Fig. S5:** Maximum likelihood tree generated with RAXML with 1,000 bootstraps from samples with a minimum mean of 3x read depth of 80\% of positions in the mitochondrial genome. Arrows indicating A-clade subclades and X-clade. The outgroup and Chinese wolves are included in figure.

**Fig. S6:** Maximum likelihood tree generated with RAXML with 1,000 bootstraps from samples with a minimum mean of 10x read depth of 95\% of sites in the mitochondrial genome. A-clade subclades and X-clade are indicated with arrows.

**Fig. S7:** Bayesian mitochondrial phylogeny constructed with BEAST2.4 for A-clade with dated nodes from samples with a minimum mean of 10x read depth. The subclades of the A-clade have been coloured according with Fig. 2, purple for clade A2b, blue for clade A2a, red for clade A1b, and yellow for clade A1a.

**Fig. S8:** Bayesian mitochondrial phylogeny constructed with BEAST2.4 for whole dataset with dated nodes from samples with a minimum mean of 10x read depth. The subclades of the A-clade have been coloured according with Fig. 2, purple for clade A2b, blue for clade A2a, red for clade A1b, and yellow for clade A1a.

**Fig. S9:** Bayesian skyline plots corresponding to Inuit and Historical/Modern sample sets from the North American Arctic. Each plot displates Ne against from 15,000 years ago to present. The black line represents the average Ne and the 95\% HPD is depicted with the purple area. A) North American Arctic dogs from 300 years ago until 50 years before present. B) North American Arctic dogs from 300 years ago until present. C) Samples of only North American dogs from Inuit contexts. D) Combined dataset of Inuit dogs until modern dogs in the North American Arctic.

**Fig. S10:** Nuclear ancestry analyses of AL2797 (Thule sample from Nunalleq). A-B. Principal component analysis of nuclear SNP data with (A) and without (B) coyotes genomes. This data set comprises 49 canids genomes (see Table X for accession numbers and sample provenance), including an outgroup (Andean fox) 2 coyotes (C\_MidW, C\_Cal), 10 modern Arctic, pre-contact dogs and CTVT genomes (D\_AHusky91, D\_Green, D\_Husky, D\_Husky89, D\_Mal68, AL3194, AL3223, C\_399T, C\_79T, C\_24T), 9 modern East Asian dogs (D\_China8, D\_Tibet3, D\_Tibet4, D\_TMastif4, D\_TMastif5, D\_China9, D\_Dingo, D\_Viet21, D\_Viet59) and 17 Western dogs (D\_India168, D\_India60, D\_SLaika, D\_Na89, D\_Basenji, D\_Na8, D\_Qatar27, D\_Qatar5, D\_NGDG, D\_Peru, D\_Port71, D\_Mex, D\_Port61, D\_Leb85, D\_Leb79, D\_GerShep6, D\_GerShep3), 6 modern Eurasian wolves (W\_Iran, W\_India, W\_Spa, W\_Port, W\_Mongo, W\_Altai), 1 ancient Eurasian wolf (TAI) and 3 modern American wolves (W\_Mex1, W\_Yellow1, W\_Yellow2). C. Neighbour joining tree tree depicting the relationship between these genomes D. D-statistics of the form D(AndeanFox, AL2797, Pop3, Pop4) where Pop3 was fixed as either Western dogs, Asian dogs, Arctic dogs, or CTVT (coloured box plot) and Pop4 represents the genome on the y-axis.

**Fig. S11:** Estimated *F*_ST_ and statistical significance were computed using absolute haplogroup frequencies (i.e., sample sizes were accounted for) in Arlequin 3.5.1.3. *F*_ST_ ranges from 0 to 1 with 0 reflecting no difference in haplogroup frequencies and 1 reflecting the greatest possible difference. Statistical significance was denoted by asterisks (*** P < 0.001, *P < 0.05).

**Fig. S12:** A) A median joining network of historical and modern haplotypes from North American Arctic dogs constructed using Network v5.0.1.0., illustrating that subclades A2a and A2b are more deeply rooted than subclades A1a or A1b; rho estimates indicate the average number of mutations accumulating since divergence from the root haplotype (too few samples from A2b were present in historical dogs to estimate rho). B) The relatively recent derivation of A1a and A1b haplotypes (past ~200 years) relative to A2a haplotypes with ages based on calibration to the North American A2a subclade assuming it began accumulating the observed mutations at least 1,000 years ago (rho minus 1 SD = 1000 years), coinciding with the Inuit expansion.

**Fig. S13:** Maps showing the sampling location and overall sample sizes for the three elements analysed using GMM methods.

**Fig. S14:** (top) Images of the canid crania showing the placement of the 30 permanent 3D landmarks. 2D landmark configurations for the (middle) the lower M1 showing permanent landmarks (red) and 2 curves of sliding semi-landmarks (grey) and (bottom) mandible showing 15 permanent landmarks (red).

**Fig. S15** Two first axes of Principal component analyses of the Crania (top), lower M1 (middle), and mandible (bottom).

**Fig. S16** Two first axes of Canonical Variate Analyses of the Crania (top), lower M1 (middle), and mandible (bottom).

**Fig. S17:** Bone collagen $\delta$^13^C and $\delta$^15^N data for the canids from the Nunalleq Site. The wolf is labelled (AL2797), and shows a diet with less marine protein than the dogs from the site.

## Captions for Databases S1 - S7

**Table S1:** Data table listing all specimens used in for DNA analysis, including group assignment, type of element analysed, and sample collection location. (Excel)

**Table S2:** Data table listing all published mitochondrial sequences used as a reference panel. (Excel)

**Table S3:** Data table listing all specimens used for GMM analysis, including group assignment, type of element analysed, and sample collection location. Sheets two to four include the raw coordinates used for the GMM analyses. (Excel)

**Table S4:** Results of carbon and nitrogen stable isotope analysis, including the 7 new samples generated for this analysis, as well as those from [(E. McManus-Fry et al. 2016)](https://paperpile.com/c/GftuPk/FLte6). (Excel)

**Table S5:** Radiocarbon dates generated for this project from Deering, Seward Peninsula, Kotzebue Sound, Alaska; Silumiut site (KkJg-2), northwestern Hudson Bay, Nunavut and Boisman II, Primorsky Siberia. (Excel)

**Table S6**: Nuclear genomes used for the analysis of AL2797 (excel)

## SI References

[Adams, D. C., M. L. Collyer, and A. Kaliontzopoulou. 2018. “Geomorph: Software for Geometric Morphometric Analyses.” *R Package Version 3.0.6.*](http://paperpile.com/b/GftuPk/ikIdF) <https://cran.r-project.org/package=geomorph.>

[Alix, Claire, Owen K. Mason, Nancy H. Bigelow, Shelby L. Anderson, Jeffrey Rasic, and John F. Hoffecker. 2015. “Archéologie Du Cap Espenberg Ou La Question Du Birnirk et de L’origine Du Thule Dans Le Nord-Ouest de l’Alaska.” *Nouvelles Orientations Dans Les Peches. Serie de Notes de Synthese Sur Les Questions de Developpement*, no. 141: 13–19.](http://paperpile.com/b/GftuPk/Xa015)

[Allen, G. 1920. *Dogs of the American Aborigines*. Vol. 63. Bulletin of the Museum of Comparative Zoology at Harvard College.](http://paperpile.com/b/GftuPk/0fGFw)

[Ambrose, Stanley H. 1990. “Preparation and Characterization of Bone and Tooth Collagen for Isotopic Analysis.” *Journal of Archaeological Science* 17 (4): 431–51.](http://paperpile.com/b/GftuPk/NqBfu)

[Amorosi, T. 1996. “Icelandic Zooarchaeology: New Data Applied to Issues of Historical Ecology, Paleoeconomy and Global Change.” The City University of New York, New York.](http://paperpile.com/b/GftuPk/tCeif)

[Amorosi, T., P. C. Buckland, Ólafsson G., J. P. Sadler, and P. Skidmore. 1992. “Site Status and the Palaeoecological Record. Glasgow:” In *Norse and Later Settlement and Subsistence in the North Atlantic.*, edited by C. D. Morris and D. J. Rackham. University of Glasgow, Dept. of Archaeology.](http://paperpile.com/b/GftuPk/oXybd)

[Anderson, Douglas D. 1988. *Onion Portage: An Archaeological Site on the Kobuk River, Northwestern Alaska*. University of Alaska Press.](http://paperpile.com/b/GftuPk/G72rU)

[Arendt, Beatrix. 2013. “The Return to Hopedale: Excavations at Anniowaktook Island, Hopedale, Labrador.” *Canadian Journal of Archaeology* 37 (-): 302–30.](http://paperpile.com/b/GftuPk/zzfbP)

[Arnold, Charles D. 1979. “Possible Evidence of Domestic Dog in a Paleoeskimo Context.” *Arctic* 32 (3): 263–65.](http://paperpile.com/b/GftuPk/iIj4y)

[———. 1980. “A Paleoeskimo Occupation on Southern Banks Island, NWT.” *Arctic*, 400–426.](http://paperpile.com/b/GftuPk/fQIXT)

[———. 1994. “Investigation on Richards Island.” In *Bridges Across Time: The NOGAP Archaeology Project*, edited by Jean-Luc Pilon, 85–93. Canadian Archaeological Association Occasional Paper 2.](http://paperpile.com/b/GftuPk/9elav)

[———. 2010. “Archaeological Investigations near Fish Lake, Southwestern Banks Island.” *Unpublished Report on File at the Prince of Wales Northern Heritage Centre, Yellowknife.*](http://paperpile.com/b/GftuPk/ypKpG)

[Arutiunov, S. A., & Sergeev, D. A. 1990. “Chapter 1.” In *Issues of the Ethnic History of the Bering Sea.*, edited by Arutiunov, S. A., & Sergeev, D. A., 50–61.](http://paperpile.com/b/GftuPk/8srrG)

[Balkwill, Darlene, and Anne Rick. 1994. “Siglit Subsistence: Preliminary Report on Faunal Remains from a Large Midden at the Gupuk Site (NiTs-1), Mackenzie Delta, NWT.” *Bridges Across Time: The NOGAP Archaeology Project*, 95–116.](http://paperpile.com/b/GftuPk/ttunl)

[Bandi, Hans Georg, and Reto Blumer. 2002. “Investigations by Swiss Archaeologists on St. Lawrence Island. InD. E. Dumond and RL Bland.” *Archaeology in the Bering Strait Region: Research on Two Continents*, 25–60.](http://paperpile.com/b/GftuPk/IrP7o)

[Baylac, Michel, and Martin Friess. 2005. “Fourier Descriptors , Procrustes Superimposition , and Data Dimensionality : An Example of Cranial Human Populations.” *Modern Morphometrics in Physical Anthropology*, 145–65.](http://paperpile.com/b/GftuPk/S8kCT)

[Betts, Matthew W. 2008. *Subsistence and Culture in the Western Canadian Arctic: A Multicontextual Approach*. Canadian Mus of Civilization.](http://paperpile.com/b/GftuPk/nzphP)

[Betts, M. W., M. Burchell, and B. R. Schöne. 2017. “An Economic History of the Maritime Woodland Period in Port Joli Harbour, Nova Scotia” 10 (sp10): 18–41.](http://paperpile.com/b/GftuPk/rwmHX)

[Binford, L. R. 1978. *Nunamiut: Ethnoarchaeology*. New York: Academic Press.](http://paperpile.com/b/GftuPk/8BiOA)

[Botigue, Laura, Shiya Song, Amelie Scheu, Shyamalika Gopalan, Amanda Pendleton, Matthew Oetjens, Angela Taravella, et al. 2016. “Ancient European Dog Genomes Reveal Continuity since the Early Neolithic.” *bioRxiv*. https://doi.org/](http://paperpile.com/b/GftuPk/DI1p6)[10.1101/068189](http://dx.doi.org/10.1101/068189)[.](http://paperpile.com/b/GftuPk/DI1p6)

[Bowers, Peter M., and Catherine M. Williams. 2009. “The Archaeology of Deering, Alaska: Final Report on the Deering Village Safe Water Archaeological Program.”](http://paperpile.com/b/GftuPk/8LcB2)

[Bowers, P. M., and M. Moss. 2001. “The North Point Wet Site and the Subsistence Importance of Pacific Cod on the Northern Northwest Coast.” In *People and Wildlife in Northern North America: Essays in Honor of R. Dale Guthrie*, edited by S. Craig Gerlach and M. S. Murray, 159–77. 944. Oxford: BAR International Series.](http://paperpile.com/b/GftuPk/rsaoh)

[Bowers, P. M., C. M. Williams, R. C. Betts, O. K. Mason, R. T. Gould, and M. L. Moss. 2001. “The North Point Site: Archaeological Investigations of a Prehistoric Wet Site at Port Houghton, Alaska.” *Report Prepared for the USDA Forest Service, Tongass National Forest, Sitka* Prepared by Northern Land Use Research, Inc., Fairbanks.](http://paperpile.com/b/GftuPk/3nReU)

[Britton, Kate, Rick Knecht, Olaf Nehlich, Charlotta Hillerdal, Richard S. Davis, and Michael Richards. 2013. “Maritime Adaptations and Dietary Variation in Prehistoric Western Alaska: Stable Isotope Analysis of Permafrost-Preserved Human Hair.” *American Journal of Physical Anthropology* 151 (3): 448–61.](http://paperpile.com/b/GftuPk/B31FT)

[Bronshtein, M. M., and K. A. Dneprovsky. 2002. “The Northeastern Chukchi Peninsula during the Birnirk and Early Punuk Periods.” In *Archaeology in the Bering Strait Region: Research on Two Continents*, edited by D. E. Dumon and R. L. Bland, 153–65. University of Oregon Anthropological Papers. University of Oregon.](http://paperpile.com/b/GftuPk/6OBfn)

[Brown, Sarah K., Christyann M. Darwent, and Benjamin N. Sacks. 2013. “Ancient DNA Evidence for Genetic Continuity in Arctic Dogs.” *Journal of Archaeological Science* 40 (2): 1279–88.](http://paperpile.com/b/GftuPk/61pmw)

[Brown, S. K., C. M. Darwent, E. J. Wictum, and B. N. Sacks. 2015. “Using Multiple Markers to Elucidate the Ancient, Historical and Modern Relationships among North American Arctic Dog Breeds.” *Heredity* 115 (6): 488–95.](http://paperpile.com/b/GftuPk/cBWRk)

[Brown, T. A., D. E. Nelson, J. S. Vogel, and J. R. Southon. 1988. “Improved Collagen Extraction by Modified Longin Method.” *Radiocarbon* 30 (2): 171–77.](http://paperpile.com/b/GftuPk/Qd2jf)

[Buonasera, Tammy Y., Andrew H. Tremayne, Christyann M. Darwent, Jelmer W. Eerkens, and Owen K. Mason. 2015. “Lipid Biomarkers and Compound Specific δ13C Analysis Indicate Early Development of a Dual-Economic System for the Arctic Small Tool Tradition in Northern Alaska.” *Journal of Archaeological Science* 61 (September): 129–38.](http://paperpile.com/b/GftuPk/HnBDR)

[Carlson, Eric S., Howard L. Smith, Peter M. Bowers, Sarah McGowan, and Holly J. McKinney. 2013. “Kotzebue Shore Avenue Reconstruction Project: Construction Monitoring Cultural Resource Project.” *Report Prepared for the Alaska Interstate Construction, LLC, Anchorage, Alaska, by Northern Land Use Research, Inc., Fairbanks, Alaska.*](http://paperpile.com/b/GftuPk/jCT1u)

[Carøe, Christian, Shyam Gopalakrishnan, Lasse Vinner, Sarah S. T. Mak, Mikkel Holger S. Sinding, José A. Samaniego, Nathan Wales, Thomas Sicheritz-Pontén, and M. Thomas P. Gilbert. 2018. “Single-Tube Library Preparation for Degraded DNA.” Edited by Susan Johnston. *Methods in Ecology and Evolution / British Ecological Society* 9 (2): 410–19.](http://paperpile.com/b/GftuPk/IMJda)

[Carter, Wilbert. 1966. “Archaeological Survey of Eskimo, or Earlier, Material in the Vicinity of Point Barrow, Alaska.” *Final Report to the Office of Naval Research and Arctic Institute of North America.*](http://paperpile.com/b/GftuPk/CKFka)

[Clark, Brenda. 1980. “The Lake Site (KkHh-2), Southampton Island, NWT and Its Position in Sadlermiut Prehistory.” *Canadian Journal of Archaeology/Journal Canadien d’Archéologie*, 53–81.](http://paperpile.com/b/GftuPk/SCk6l)

[Cleland, Charles Edward, and W. G. Haag. 1973. “Appendix I: Notes on the Dog Skull from Nanook Component 2.” *Maxwell, MS Archaeology of the Lake Harbour District, Baffin Island. Mercury Series, Archaeological Survey of Canada Paper* 6: 353–56.](http://paperpile.com/b/GftuPk/K9Vlj)

[Collins, H. B. 1951. “Dr H. B. Collins’ Archaeological Excavations at Frobisher Bay, Baffin Island, and at Resolute Bay, Cornwallis Island, 1948 and 1949.” *The Polar Record* 6 (42): 255–255.](http://paperpile.com/b/GftuPk/jqEYQ)

[Collins, M. J., and P. Galley. 1998. “Towards an Optimal Method of Archaeological Collagen Extraction: The Influence of pH and Grinding.” *Ancient Biomolecules* 2 (2/3): 209–23.](http://paperpile.com/b/GftuPk/14wi1)

[Cooper, A., and H. N. Poinar. 2000. “Ancient DNA: Do It Right or Not at All.” *Science* 289 (5482): 1139.](http://paperpile.com/b/GftuPk/Zwu5M)

[Cooper, H. Kory, Owen K. Mason, Victor Mair, John F. Hoffecker, and Robert J. Speakman. 2016. “Evidence of Eurasian Metal Alloys on the Alaskan Coast in Prehistory.” *Journal of Archaeological Science* 74 (October): 176–83.](http://paperpile.com/b/GftuPk/aEX8h)

[Csonka, Yvon. 2014. *The Ekven Settlement: Eskimo Beginnings on the Asian Shore of Bering Strait*. Archaeopress.](http://paperpile.com/b/GftuPk/5YFch)

[Dabney, Jesse, Michael Knapp, Isabelle Glocke, Marie-Theres Gansauge, Antje Weihmann, Birgit Nickel, Cristina Valdiosera, et al. 2013. “Complete Mitochondrial Genome Sequence of a Middle Pleistocene Cave Bear Reconstructed from Ultrashort DNA Fragments.” *Proceedings of the National Academy of Sciences of the United States of America* 110 (39): 15758–63.](http://paperpile.com/b/GftuPk/BiiZx)

[Damgaard, Peter B., Ashot Margaryan, Hannes Schroeder, Ludovic Orlando, Eske Willerslev, and Morten E. Allentoft. 2015. “Improving Access to Endogenous DNA in Ancient Bones and Teeth.” *Scientific Reports* 5 (June): 11184.](http://paperpile.com/b/GftuPk/bA0Oq)

[Darwent, Christyann M. 2006. “Reassessing the Old Whaling Locality at Cape Krusenstern, Alaska.” In *Dynamics of Northern Societies. Proceedings of the SILA/NABO Conference on Arctic and North Atlantic Archaeology, Copenhagen, May 10th–14th, 2004,* edited by Jette Arneborg and Bjarne Grønnow, 10:95–102. Studies in Archaeology and History. Copenhagen.: PNM, Publications from the National Museum.](http://paperpile.com/b/GftuPk/kJd1S)

[Darwent, Christyann Marie. 2002. “High Arctic Paleoeskimo Fauna: Temporal Changes and Regional Differences (Canada, Greenland).”](http://paperpile.com/b/GftuPk/fje2b) <https://elibrary.ru/item.asp?id=6690866>[.](http://paperpile.com/b/GftuPk/fje2b)

[Darwent, Christyann M., and John Darwent. 2016. “The Enigmatic Choris and Old Whaling ‘Cultures’ of the Western Arctic.” *The Oxford Handbook of the Prehistoric Arctic. Oxford University Press, New York*, 371–94.](http://paperpile.com/b/GftuPk/U0Pyf)

[Darwent, John, and Christyann M. Darwent. 2005. “Occupational History of the Old Whaling Site at Cape Krusenstern, Alaska.” *Alaska Journal of Anthropology* 3 (2): 135–54.](http://paperpile.com/b/GftuPk/NejOP)

[Darwent, John, Owen K. Mason, John F. Hoffecker, and Christyann M. Darwent. 2013. “1,000 Years of House Change at Cape Espenberg, Alaska: A Case Study in Horizontal Stratigraphy.” *American Antiquity* 78 (3): 433–55.](http://paperpile.com/b/GftuPk/FV5Vq)

[Dneprovsky, K. 2006. “A Late Birnirk House at Paipelghak in Northern Chukotka: A Preliminary Report Based on the Excavations from 2002-2004.” *Alaska J. Anthropol.* 4: 34–53.](http://paperpile.com/b/GftuPk/OwLxM)

[Dneprovsky, K., and M. Bronshtein. 2002. “Ekven H-18: A Birnirk and Early Punuk-Period Site in Chukotka. Archaeology in the Bering Strait Region: Research on Two Continents.” *University of Oregon Anthropological Papers,* 59: 166–206.](http://paperpile.com/b/GftuPk/PKYIJ)

[Duggan, Ana T., Alison J. T. Harris, Stephanie Marciniak, Ingeborg Marshall, Melanie Kuch, Andrew Kitchen, Gabriel Renaud, et al. 2017. “Genetic Discontinuity between the Maritime Archaic and Beothuk Populations in Newfoundland, Canada.” *Current Biology: CB*, 1–8.](http://paperpile.com/b/GftuPk/1MQ4W)

[Enk, Jacob M., Alison M. Devault, Melanie Kuch, Yusuf E. Murgha, Jean-Marie Rouillard, and Hendrik N. Poinar. 2014. “Ancient Whole Genome Enrichment Using Baits Built from Modern DNA.” *Molecular Biology and Evolution* 31 (5): 1292–94.](http://paperpile.com/b/GftuPk/nM0ui)

[Ersmark, Erik, Ludovic Orlando, Edson Sandoval-Castellanos, Ian Barnes, Ross Barnett, Anthony Stuart, Adrian Lister, and Love Dalén. 2015. “Population Demography and Genetic Diversity in the Pleistocene Cave Lion.” https://doi.org/](http://paperpile.com/b/GftuPk/WUa6n)[10.5334/oq.aa](http://dx.doi.org/10.5334/oq.aa)[.](http://paperpile.com/b/GftuPk/WUa6n)

[Evin, Allowen, Thomas Cucchi, Andrea Cardini, Una Strand Vidarsdottir, Greger Larson, and Keith Dobney. 2013. “The Long and Winding Road: Identifying Pig Domestication through Molar Size and Shape.” *Journal of Archaeological Science* 40 (1): 735–43.](http://paperpile.com/b/GftuPk/2u38J)

[Evin, Allowen, Thibaud Souter, Ardern Hulme-Beaman, Carly Ameen, Richard Allen, Pietro Viacava, Greger Larson, Thomas Cucchi, and Keith Dobney. 2016. “The Use of Close-Range Photogrammetry in Zooarchaeology: Creating Accurate 3D Models of Wolf Crania to Study Dog Domestication.” *Journal of Archaeological Science: Reports* 9: 87–93.](http://paperpile.com/b/GftuPk/MyxQO)

[Fitzhugh, Ben, Erik Gjesfjeld, William Brown, Mark J. Hudson, and Jennie D. Shaw. 2016. “Resilience and the Population History of the Kuril Islands, Northwest Pacific: A Study in Complex Human Ecodynamics.” *Quaternary International: The Journal of the International Union for Quaternary Research* 419 (October): 165–93.](http://paperpile.com/b/GftuPk/K1aGf)

[Fitzhugh, B., M. Etnier, B. MacInnes, S. C. Phillips, and J. Taylor. 2007. “Report of Archaeological Field Research in 2006, Including Geological Descriptions of Archaeological Locales (Unpublished Project Report for Kuril Biocomplexity Project).” University of Washington (tDAR id: 376122); https://doi.org/](http://paperpile.com/b/GftuPk/X8BB0)[10.6067/XCV8PK0FCF](http://dx.doi.org/10.6067/XCV8PK0FCF)[.](http://paperpile.com/b/GftuPk/X8BB0)

[{Fitzhugh, B., M. Etnier, S. C. Phillips, and E. }. Gjesfjeld. 2009. “Report of Archaeological Field Research in 2008, Including Geological Descriptions of Archaeological Locales (Unpublished Project Report for Kuril Biocomplexity Project).” *University of Washington* tDAR id: 376123. https://doi.org/](http://paperpile.com/b/GftuPk/3DP2q)[10.6067/XCV8F18XZ9](http://dx.doi.org/10.6067/XCV8F18XZ9)[.](http://paperpile.com/b/GftuPk/3DP2q)

[Fitzhugh, William W. 2016. “Solving the ‘Eskimo Problem’: Henry Bascom Collins and Arctic Archaeology.” In *Early Inuit Studies, Themes and Transitions, 1850s-1980s.*, edited by Igor Krupnik. Washington, D.C.: Smithsonian Institution Scholarly Press.](http://paperpile.com/b/GftuPk/MMNn9)

[Flegontov, Pavel, Nefize Ezgi Altinisik, Piya Changmai, Nadin Rohland, Swapan Mallick, Deborah A. Bolnick, Francesca Candilio, et al. 2017. “Paleo-Eskimo Genetic Legacy across North America.” *bioRxiv*. https://doi.org/](http://paperpile.com/b/GftuPk/spLl6)[10.1101/203018](http://dx.doi.org/10.1101/203018)[.](http://paperpile.com/b/GftuPk/spLl6)

[Forbes, Véronique, Kate Britton, and Rick Knecht. 2015. “Preliminary Archaeoentomological Analyses of Permafrost-Preserved Cultural Layers from the Pre-Contact Yup’ik Eskimo Site of Nunalleq , Alaska: Implications, Potential and Methodological Considerations.” *Environmental Archaeology* 20 (2): 158–67.](http://paperpile.com/b/GftuPk/gIedR)

[Ford, J. A. 1959. “Eskimo Prehistory in the Vicinity of Point Barrow, Alaska.” *Anthropological Papers of the American Museum of Natural History* 47 (1): 1–272.](http://paperpile.com/b/GftuPk/7c5go)

[Frantz, Laurent A. F., Victoria E. Mullin, Maud Pionnier-Capitan, Ophélie Lebrasseur, Morgane Ollivier, Angela Perri, Anna Linderholm, et al. 2016. “Genomic and Archaeological Evidence Suggest a Dual Origin of Domestic Dogs.” *Science* 352 (6290): 1228–31.](http://paperpile.com/b/GftuPk/iMyeI)

[Friesen, T. M. A. X. 2009. “PAN-ARCTIC POPULATION MOVEMENTS The Early Paleo-Inuit and Thule Inuit Migrations.” In *The Oxford Handbook of the Prehistoric Arctic*, edited by Max Friesen and Owen Mason.](http://paperpile.com/b/GftuPk/iv6Du)

[Friesen, T. Max. 2015. “On the Naming of Arctic Archaeological Traditions: The Case for Paleo-Inuit.” *Arctic* 68 (3).](http://paperpile.com/b/GftuPk/dq5oX) <http://arctic.journalhosting.ucalgary.ca/arctic/index.php/arctic/article/download/4504/4622>[.](http://paperpile.com/b/GftuPk/dq5oX)

[Friesen, T. Max, and Charles D. Arnold. 1995. “Zooarchaeology of a Focal Resource: Dietary Importance of Beluga Whales to the Precontact Mackenzie Inuit.” *Arctic* 48 (1): 22–30.](http://paperpile.com/b/GftuPk/Int0o)

[Friesen, T. Max, and Matthew Betts. 2006. “Archaeofaunas and Architecture: Zooarchaeological Variability within an Inuit Semi-Subterranean House, Arctic Canada.” In *Integrating Zooarchaeology*, edited by Mark Maltby, 65–76. Oxford: Oxbow Press.](http://paperpile.com/b/GftuPk/CtcqD)

[Garðarsdóttir, V. B. 2010. “Fornleifauppgröftur á Alþingisreitnum 2008-2010.” *Reykjavík: Alþingi Og Framkvæmdasýsla Ríkisins.*](http://paperpile.com/b/GftuPk/kRX4X)

[———. 2011. “Alþingisreiturinn: Upphaf Landnáms í Reykjavík.” *Árbók Hins íslenzka Fornleifafélags, 2011, 5–43.*](http://paperpile.com/b/GftuPk/fTAgk)

[———. 2013. “Alþingisreitur 2012.” *Reykjavík.*](http://paperpile.com/b/GftuPk/d8rdS)

[Geist, Otto William, and Froelich G. Rainey. 1936. “Archaeological Excavations at Kukulik, St. Lawrence Island, Alaska.” *Government Printing Office, Washington, DC*.](http://paperpile.com/b/GftuPk/7c6vu)

[Giddings, J. 1973. “Louis 1964 The Archaeology of Cape Denbigh.” *Brown UniversityPress, Providence*.](http://paperpile.com/b/GftuPk/rgXZA)

[Giddings, J. L. 1957. “Round Houses in the Western Arctic.” *American Antiquity* 23 (2Part1): 121–35.](http://paperpile.com/b/GftuPk/BxIqM)

[———. 1960. “The Archeology of Bering Strait.” *Current Anthropology* 1 (2): 121.](http://paperpile.com/b/GftuPk/UrmPd)

[Giddings, J. Louis. 1952. *The Arctic Woodland Culture of the Kobuk River*. Museum Monographs,. Philadelphia: University Museum, University of Pennsylvania,.](http://paperpile.com/b/GftuPk/RIzAU)

[Giddings, J. Louis, and Douglas D. Anderson. 1986. “Beach Ridge Archaeology of Cape Krusenstern: Eskimo and Pre-Eskimo Settlements around Kotzebue Sound Alaska.” *Publications in Archaeology 20. National Park Service* U.S. Department of the Interior, Washington, D.C.](http://paperpile.com/b/GftuPk/grz9v)

[Gilbert, M. Thomas P., Hans-Jürgen Bandelt, Michael Hofreiter, and Ian Barnes. 2005. “Assessing Ancient DNA Studies.” *Trends in Ecology & Evolution* 20 (10): 541–44.](http://paperpile.com/b/GftuPk/BThHl)

[Gilbert, M. Thomas P., Lynn P. Tomsho, Snjezana Rendulic, Michael Packard, Daniela I. Drautz, Andrei Sher, Alexei Tikhonov, et al. 2007. “Whole-Genome Shotgun Sequencing of Mitochondria from Ancient Hair Shafts.” *Science* 317 (5846): 1927–30.](http://paperpile.com/b/GftuPk/uLvgH)

[Glob, P. V. 1935. *Eskimo Settlements in Kempe Fjord and King Oskar Fjord, with Zoological Appendix by M. Degerbøl*. Vol. 102 No. 2. Meddelelser Om Grønland . Copenhagen.: C.A. Reitzel.](http://paperpile.com/b/GftuPk/5nUzj)

[Goodall, C. R. 1995. “Procrustes Methods in the Statistical Analysis of Shape Revisited.” *Current Issues in Statistical Shape Analysis*, 18–33.](http://paperpile.com/b/GftuPk/eF8ma)

[Gotfredsen, Anne Birgitte. 2010. “Faunal Remains from the Wollaston Forland – Clavering Ø Region, Northeast Greenland – Thule Culture Subsistence in a High Arctic Polynya and Ice-Edge Habitat.” *Geografisk Tidsskrift-Danish Journal of Geography* 110 (2): 175–200.](http://paperpile.com/b/GftuPk/Oa3Pg)

[Gotfredsen, Anne Birgitte, and Tinna Møbjerg. 2010. “Nipisat-a Saqqaq Culture Site in Sisimiut, Central West Greenland (Vol. 331).”](http://paperpile.com/b/GftuPk/3lfd4) <http://www.oapen.org/search?identifier=342365>[.](http://paperpile.com/b/GftuPk/3lfd4)

[Grønnow, Bjarne. 1994. “Qeqertasussuk-the Archaeology of a Frozen Saqqaq Site in Disko Bugt, West Greenland.” *Threads of Arctic Prehistory: Papers in Honour of William E. Taylor Jr. Ottawa, Canada: Canadian Museum of Civilization*, 197–238.](http://paperpile.com/b/GftuPk/ISyIc)

[———. 2017. *The Frozen Saqqaq Sites of Disko Bay, West Greenland. Qeqertasussuk and Qajaa (2400 – 900 BC)*. Monographs on Greenland. Museum Tusculanum Press.](http://paperpile.com/b/GftuPk/m8k3f)

[Gronnow, B., and J. F. Jensen. 2009. *The Northernmost Ruins of the Globe*.](http://paperpile.com/b/GftuPk/k6dN1)

[Gullov, Hans Christian. 1997. *From Middle Ages to Colonial Times*. Museum Tusculanum Press.](http://paperpile.com/b/GftuPk/lhkfj)

[Gusev, Sergey V., Andrey V. Zagoroulko, and Aleksey V. Porotov. 1999. “Sea Mammal Hunters of Chukotka, Bering Strait: Recent Archaeological Results and Problems.” *World Archaeology* 30 (3): 354–69.](http://paperpile.com/b/GftuPk/1wdkG)

[Harrison, R. 2009. “The Gásir Area A Archaeofauna: An Update of the Results from the Faunal Analysis of the High Medieval Trading Site in Eyjafjörður, N Iceland.”](http://paperpile.com/b/GftuPk/icj5O)

[———. 2011. “Myrkárdalur in Hörgárdalur, N. Iceland: Brief Summary of the 2008/2009 Archaeofauna.” *NORSEC/HERC Zooarchaeology Laboratory Report* 57.](http://paperpile.com/b/GftuPk/3ubRL)

[———. 2014. *Connecting the Land to the Sea at Gásir. Long-Term Human Ecodynamics in the North Atlantic: An Archaeological Study*. Vol. 117–36. Lanham, Maryland, : Lexington Publishers.](http://paperpile.com/b/GftuPk/uH4SD)

[Hays, J. M., J. D. Reuther, C. Wooley, J. S. Rogers, R. C. Bowman, J. Baxter-McIntosh, and M. M. Proue. 2012. “Life on the River: Community Archaeology at SLT-094, Middle Kuskokwim River, Alaska.” *Report Prepared by Northern Land Use Research, Inc., Fairbanks, and Chumis Cultural Resource Services, Anchorage.*](http://paperpile.com/b/GftuPk/H79y3)

[Heizer, Robert Fleming. 1956. *Archaeology of the Uyak Site, Kodiak Island, Alaska*. Vol. 17. University of California Press.](http://paperpile.com/b/GftuPk/62auz)

[Helmer, James W. 1991. “The Palaeo-Eskimo Prehistory of the North Devon Lowlands.” *Arctic* 44 (4): 301–17.](http://paperpile.com/b/GftuPk/rWm09)

[Hicks, Megan, Frank Feeley Adolf Friðriksson, George Hambrecht, Lilja Pálsdottir, Garðar Guðmundsson, and Magnus Á. Sigurgeirsson. 2013. “Midden Excavations at Skútustaðir N. Iceland, 2011. Report FS508-08274.” *Fornleifastofnun Íslands Institute of Archaeology, Reykjavík.*](http://paperpile.com/b/GftuPk/BAhOS)

[Hill, Erica. 2018. “Humans , Birds and Burial Practices at Ipiutak , Alaska : Perspectivism in the Western Arctic Humans , Birds and Burial Practices at Ipiutak , Alaska : Perspectivism in the Western Arctic.” *Environmental Archaeology* 0 (0): 1–15.](http://paperpile.com/b/GftuPk/UfdM7)

[Hodgetts, Lisa M., and Edward J. H. Eastaugh. 2017. “The Role of Magnetometry in Managing Arctic Archaeological Sites in the Face of Climate Change.” *Advances in Archaeological Practice* 5 (2): 110–24.](http://paperpile.com/b/GftuPk/I5JXY)

[Houlette, Chris. 2009. “Lost in the Collection: Reconsidering the Meat Cache 35 Assemblage and the Question of a Thule Occupation at Kukulik.” *Alaska Journal of Anthropology* 7 (2): 101–20.](http://paperpile.com/b/GftuPk/O8UAV)

[Hrdlicka, Ales. 1928. “Explorations in Alaska.” *46th Annual Report. Bureau of American Ethnography*.](http://paperpile.com/b/GftuPk/f3qiz)

[———. 1930. “Anthropological Survey in Alaska.” *46th Annual Report of the Bureau of American Ethnology to the Secretary of the Smithsonian Institution,1928-1929. Government Printing Office, Washington, D.C.*, 19–347.](http://paperpile.com/b/GftuPk/VqOUM)

[Imes, Donna L., Elizabeth J. Wictum, Marc W. Allard, and Benjamin N. Sacks. 2012. “Identification of Single Nucleotide Polymorphisms within the mtDNA Genome of the Domestic Dog to Discriminate Individuals with Common HVI Haplotypes.” *Forensic Science International. Genetics* 6 (5): 630–39.](http://paperpile.com/b/GftuPk/jEaD0)

[Irving, W. N. 1953. *Evidence of Early Tundra Cultures in Northern Alaska*. University of Alaska.](http://paperpile.com/b/GftuPk/zxgYz)

[Jensen, Anne M. 2009. “Nuvuk, Point Barrow, Alaska: The Thule Cemetery and Ipiutak Occupation.” Edited by Richard S. Davis. Ann Arbor, United States: Bryn Mawr College.](http://paperpile.com/b/GftuPk/lXFas) <https://search.proquest.com/docview/304833059>[.](http://paperpile.com/b/GftuPk/lXFas)

[———. 2016. “Archaeology of the Late Western Thule/Iñupiat in North Alaska (a.d. 1300–1750).” In *The Oxford Handbook of the Prehistoric Arctic*, edited by Max Friesen And. Oxford University Press.](http://paperpile.com/b/GftuPk/uvQRw)

[Johansen, Trine B. 2012. “A Zoorchaeological and Ethnographic Investigation of Subsistence Change through Time at Iita, Northwest Greenland.” Ph.D. Dissertation, University of California, Davis.](http://paperpile.com/b/GftuPk/2Q6eB)

[Johnson, Donald Mcintosh. 1933. “Observations on the Eskimo Remains on the East Coast of Greenland Between 72 and 75 North Latitude.” *Meddelelser Om Grønland* 92 (6).](http://paperpile.com/b/GftuPk/Rjjhp)

[Jónsson, Hákon, Aurélien Ginolhac, Mikkel Schubert, Philip L. F. Johnson, and Ludovic Orlando. 2013. “mapDamage2.0: Fast Approximate Bayesian Estimates of Ancient DNA Damage Parameters.” *Bioinformatics*  29 (13): 1682–84.](http://paperpile.com/b/GftuPk/P9vRP)

[Kaplan, Susan A. 1980. “Neo-Eskimo Occupations of the Northern Labrador Coast.” *Arctic* 33 (3): 646–58.](http://paperpile.com/b/GftuPk/QhDzH)

[———. 2012. “Labrador Inuit Ingenuity and Resourcefulness: Adapting to a Complex Environmental, Social, and Spiritual Environment.” In *Settlement, Subsistence, and Change among the Labrador Inuit: The Nunatsiavummiut Experience*, edited by David C. Natcher, Lawrence Felt, and Andrea Procter, 15–42. University of Manitoba Press, Winnipeg.](http://paperpile.com/b/GftuPk/lrnwD)

[Kaplan, Susan A., and James M. Woollett. 2000. “Challenges and Choices: Exploring the Interplay between Climate, History, and Culture on Canada’s Labrador Coast.” *Arctic, Antarctic, and Alpine Research* 32 (3): 351–59.](http://paperpile.com/b/GftuPk/47lcT)

[Katoh, Kazutaka, and Daron M. Standley. 2013. “MAFFT Multiple Sequence Alignment Software Version 7: Improvements in Performance and Usability.” *Molecular Biology and Evolution* 30 (4): 772–80.](http://paperpile.com/b/GftuPk/7sgmG)

[Kemp, Brian M., Cara Monroe, and David Glenn Smith. 2006. “Repeat Silica Extraction: A Simple Technique for the Removal of PCR Inhibitors from DNA Extracts.” *Journal of Archaeological Science* 33 (12): 1680–89.](http://paperpile.com/b/GftuPk/A3FBd)

[Kim, K. S., S. E. Lee, H. W. Jeong, and J. H. Ha. 1998. “The Complete Nucleotide Sequence of the Domestic Dog (Canis Familiaris) Mitochondrial Genome.” *Molecular Phylogenetics and Evolution* 10 (2): 210–20.](http://paperpile.com/b/GftuPk/hTu0W)

[Klinken, G. J. van. 1999. “Bone Collagen Quality Indicators for Paleodietary and Radiocarbon Measurements.” *Journal of Archaeological Science* 26 (6): 687–96.](http://paperpile.com/b/GftuPk/PEDCD)

[Kuzmin, Yaroslav V. 1997. “Vertebrate Animal Remains from Prehistoric and Medieval Settlements in Primorye (Russian Far East).” *International Journal of Osteoarchaeology* 7 (2): 172–80.](http://paperpile.com/b/GftuPk/B23BZ)

[Laguna, Frederica de. 1936. “An Archaeological Reconnaissance of the Middle and Lower Yukon Valley, Alaska 2.” *American Antiquity* 2 (1): 6–12.](http://paperpile.com/b/GftuPk/qLpAh)

[Larsen, Helge. 2001. *Deering: A Men’s House from Seward Peninsula, Alaska*. Edited by Martin Appelt. Department of Ethnography, the National Museum of Denmark.](http://paperpile.com/b/GftuPk/q9sp)

[Larsen, Helge, and Thorvald Sørensen. 1934. *Dødemandsbugten: An Eskimo Settlement on Clavering Island; Treaarsexpeditionen Til Christian Den X’s Land 1931-34*. Reitzel.](http://paperpile.com/b/GftuPk/vKntg)

[Ledger, Paul M., Véronique Forbes, Edouard Masson-Maclean, Charlotta Hillerdal, W. Derek Hamilton, Ellen McManus-Fry, Ana Jorge, Kate Britton, and Richard A. Knecht. 2018. “THREE GENERATIONS UNDER ONE ROOF? BAYESIAN MODELING OF RADIOCARBON DATA FROM NUNALLEQ, YUKON-KUSKOKWIM DELTA, ALASKA.” *American Antiquity* 83 (3): 505–24.](http://paperpile.com/b/GftuPk/TYBnC)

[Ledger, Paul M., Véronique Forbes, Edouard Masson-maclean, and Richard A. Knecht. 2016. “Dating and Digging Stratified Archaeology in Circumpolar North America : A View from Nunalleq , Southwestern Alaska” 69 (4): 378–90.](http://paperpile.com/b/GftuPk/qo7mc)

[Lee, Esther J., D. Andrew Merriwether, Alexei K. Kasparov, Pavel A. Nikolskiy, Marina V. Sotnikova, Elena Yu Pavlova, and Vladimir V. Pitulko. 2015. “Ancient DNA Analysis of the Oldest Canid Species from the Siberian Arctic and Genetic Contribution to the Domestic Dog.” *PloS One* 10 (5): 1–13.](http://paperpile.com/b/GftuPk/GLfzM)

[LeMoine, Genevieve M., and Christyann M. Darwent. 2010. “Zooarchaeological Analysis of a Late Dorset and an Early Thule Dwelling at Cape Grinnell, Northwest Greenland.” *Geografisk Tidsskrift – Danish Journal of Geography* 100 (2): 315–36.](http://paperpile.com/b/GftuPk/ig5DN)

[Li, Heng. 2013. “Aligning Sequence Reads, Clone Sequences and Assembly Contigs with BWA-MEM.” *arXiv [q-bio.GN]*. arXiv.](http://paperpile.com/b/GftuPk/0n4PK) <http://arxiv.org/abs/1303.3997>[.](http://paperpile.com/b/GftuPk/0n4PK)

[Li, Heng, Bob Handsaker, Alec Wysoker, Tim Fennell, Jue Ruan, Nils Homer, Gabor Marth, Goncalo Abecasis, Richard Durbin, and 1000 Genome Project Data Processing Subgroup. 2009. “The Sequence Alignment/Map Format and SAMtools.” *Bioinformatics*  25 (16): 2078–79.](http://paperpile.com/b/GftuPk/htmCu)

[Lindblad-Toh, Kerstin, Claire M. Wade, Tarjei S. Mikkelsen, Elinor K. Karlsson, David B. Jaffe, Michael Kamal, Michele Clamp, et al. 2005. “Genome Sequence, Comparative Analysis and Haplotype Structure of the Domestic Dog.” *Nature* 438 (7069): 803–19.](http://paperpile.com/b/GftuPk/EAkiM)

[Lobdell, John E. 1980. “Prehistoric Human Populations and Resource Utilization in Kachemak Bay, Gulf of Alaska.” Unpublished PhD Dissertation, University of Tennessee, Knoxville.](http://paperpile.com/b/GftuPk/mqG2U)

[Longin, R. 1971. “New Method of Collagen Extraction for Radiocarbon Dating.” *Nature* 230 (5291): 241–42.](http://paperpile.com/b/GftuPk/XYBID)

[Loring, Stephen, and Beatrix Arendt. 2009. “‘… They Gave Hebron, The City of Refuge…’(Joshua 21: 13): An Archaeological Reconnaissance at Hebron, Labrador.” *Journal of the North Atlantic* 2 (sp1): 33–56.](http://paperpile.com/b/GftuPk/qNdOI)

[Mary-Rouselière, Guy. 1976. “The Paleoeskimo in Northern Baffin Island.” In *Eastern Arctic Prehistory: Paleoeskimo Problems,* edited by Moreau S. Maxwell, 31:40‒57. Memoirs of the Society for American Archaeology. Washington, D.C.: Society for American Archaeology.](http://paperpile.com/b/GftuPk/prR3D)

[———. 1979. “The Thule Culture on North Baffin Island: Early Thule Characteristics and the Survival of the Thule Tradition.” In *Thule Eskimo Culture: An Anthropological Retrospective*, edited by Alan P. McCartney, 54–75. Archaeological Survey of Canada Paper 88, National Museum of Man Mercury Series. Ottawa: National Museums of Canada.](http://paperpile.com/b/GftuPk/kTxcj)

[Mason, Owen K. 2016. “Thule Origins in the Old Bering Sea Culture: The Interrelationship of Punuk and Birnirk Cultures.” In *The Oxford Handbook of the Prehistoric Arctic*, edited by T. Max Friesen and Owen K. Mason, 489–512. Oxford: Oxford University Press.](http://paperpile.com/b/GftuPk/PF0SF)

[Mathiassen, Therkel. 1927a. *Archaeology of the Central Eskimos: II. The Thule Culture and Its Position within the Eskimo Culture*. Vol. IV. Report of the Fifth Thule Expedition 1921-24. Copenhagen: Gyldendalske.](http://paperpile.com/b/GftuPk/lGFLr)

[———. 1927b. “Archaeology of the Central Eskimos: The Thule Culture and Its Position within the Eskimo Culture. Report of the Fifth Thule Expedition 1921--24.” Nordisk Forlag Copenhagen.](http://paperpile.com/b/GftuPk/WRVMv)

[———. 1930a. “An Old Eskimo Culture in West Greenland: Report of an Archeological Expedition to Upernivik.” *Geographical Review* 20 (4): 605–14.](http://paperpile.com/b/GftuPk/2zmJW)

[———. 1930b. *Inugsuk: A Mediaeval Eskimo Settlement in Upernivik District, West Greenland*. Luno.](http://paperpile.com/b/GftuPk/Q61Eb)

[———. 1933. *Prehistory of the Angmagssalik Eskimos*. CA Reitzel.](http://paperpile.com/b/GftuPk/9NogJ)

[———. 1958. *The Sermermiut Excavations: 1955*. CA Reitzel.](http://paperpile.com/b/GftuPk/UBnPl)

[Mathiassen, T., and E. Holtved. 1936. “The Eskimo Archaeology of Julianehaab District: With a Brief Summary of the Prehistory of the Greenlanders.” *Reitzel*.](http://paperpile.com/b/GftuPk/4LaHE)

[Max Friesen, T., and Charles D. Arnold. 2008. “The Timing of the Thule Migration: New Dates from the Western Canadian Arctic.” *American Antiquity* 73 (3): 527–38.](http://paperpile.com/b/GftuPk/fTOKa)

[Maxwell, Moreau S. 1973. *Archaeology of the Lake Harbour District, Baffin Island*. Archaeological Survey of Canada, National Museum of Man, National Museums of ….](http://paperpile.com/b/GftuPk/zkj10)

[McCartney, Allen P. 1977. *Thule Eskimo Prehistory along Northwestern Hudson Bay*. National Museum of Man Mercury Series. Archaeological Survey of Canada Paper 70,. Ottawa: National Museums of Canada,.](http://paperpile.com/b/GftuPk/CoT6O)

[McCullough, Karen Margrethe. 1989. *The Ruin Islanders: Thule Culture Pioneers in the Eastern High Arctic*. Archaeological Survey of Canada. Canadian Museum of Civilization.](http://paperpile.com/b/GftuPk/7D2og)

[McGhee, Robert. 1975. “Beluga Hunters: An Archaeological Reconstruction of the History and Culture of the Mackenzie Delta Kittegaryumiut.” *Newfoundland Social and Economic Studies 13. Memorial University of Newfoundland, St. John’s.*](http://paperpile.com/b/GftuPk/DGX60)

[———. 1979. “The Paleoeskimo Occupations at Port Refuge, High Arctic Canada.” *Musée National de l’Homme. Collection Mercure. Commission Archéologique Du Canada. Publications d'Archéologie. Dossier Ottawa*, no. 92: 1–132.](http://paperpile.com/b/GftuPk/md6Mc)

[McGovern, Thomas Howatt, Konrad Smiarowski, and Ramona Harrison. 2013. “Hard Times at Hofstaðir?: An Archaeofauna circa 1300 AD from Hofstaðir in M\`yvatnssveit, N Iceland.”](http://paperpile.com/b/GftuPk/Jx3zt) <https://rafhladan.is/bitstream/handle/10802/7465/HERC-NORSEC_Report_60.pdf?sequence=1>[.](http://paperpile.com/b/GftuPk/Jx3zt)

[McGowan, Glenys, and Jonathan Prangnell. 2006. “The Significance of Vivianite in Archaeological Settings.” *Geoarchaeology* 21 (1): 93–111.](http://paperpile.com/b/GftuPk/nUM32)

[McManus-Fry, Ellen, Rick Knecht, Keith Dobney, Michael P. Richards, and Kate Britton. 2016. “Dog-Human Dietary Relationships in Yup’ik Western Alaska: The Stable Isotope and Zooarchaeological Evidence from Pre-Contact Nunalleq.” *Journal of Archaeological Science: Reports*. https://doi.org/](http://paperpile.com/b/GftuPk/FLte6)[10.1016/j.jasrep.2016.04.007](http://dx.doi.org/10.1016/j.jasrep.2016.04.007)[.](http://paperpile.com/b/GftuPk/FLte6)

[McManus-Fry, Ellen Teresa. 2015. “Pre-Contact Ecology , Subsistence and Diet on the Yukon-Kuskokwim Delta An Integrated Ecosystem Approach.” University of Aberdeen.](http://paperpile.com/b/GftuPk/N6a4z)

[Meldgaard, J. 1983. “Qaja, En Kokkenmodding I Dybfrost.” *Copenhagen: Feltrapport Fra Arbejdsmarken I Grönland, Saertryk Af National Museet, Arbejdsmark*.](http://paperpile.com/b/GftuPk/jObq7)

[Meldgaard, Jørgen. 1960. “Prehistoric Culture Sequences in the Eastern Arctic as Elucidated by Stratified Sites at Igloolik.” In *Men and Cultures*, edited by Anthony F. C. Wallace and Anthony F. C. Wallace. Philadelphia: University of Pennsylvania Press.](http://paperpile.com/b/GftuPk/Bl7oE)

[Meldgaard, Morten. 2004. *"Ancient Harp Seal Hunters of Disko Bay. Subsistence and Settlement at the Saqqaq Culture Site Qeqertasussuk (2400-1400BC), West Greenland*. Vol. 330. : Monographs on Greenland | Meddelelser Om Grønland. Museum Tusculanum Press.](http://paperpile.com/b/GftuPk/m8PEw)

[Méreuze, Rémi. 2015. “La Construction de La Maison 33 Du Cap Espenberg, Nord-Ouest de l’Alaska, Au Xviiie Siècle.” *Nouvelles Orientations Dans Les Peches. Serie de Notes de Synthese Sur Les Questions de Developpement*, no. 141 (September): 19–25.](http://paperpile.com/b/GftuPk/VpS6n)

[Meyer, Matthias, and Martin Kircher. 2010. “Illumina Sequencing Library Preparation for Highly Multiplexed Target Capture and Sequencing.” *Cold Spring Harbor Protocols* 2010 (6): db.prot5448.](http://paperpile.com/b/GftuPk/6H4Zb)

[Møhl, Jeppe. 1979. “AN ESKIMO SETTLEMENT REPRESENTATIVE OF THE THULE CULTURE IN WEST GREENLAND.” In *Thule Eskimo Culture*, 380–94. An Anthropological Retrospective. University of Ottawa Press.](http://paperpile.com/b/GftuPk/dNABD)

[———. 1986. “Dog Remains from a Paleoeskimo Settlement in West Greenland.” *Arctic Anthropology* 23 (1/2): 81–89.](http://paperpile.com/b/GftuPk/LDMJS)

[Monteiro, L. R. 1999. “Multivariate Regression Models and Geometric Morphometrics: The Search for Causal Factors in the Analysis of Shape.” *Systematic Biology* 48 (1): 192–99.](http://paperpile.com/b/GftuPk/bd8Qs)

[Moody, John F., and Lisa M. Hodgetts. 2013. “Subsistence Practices of Pioneering Thule–Inuit: A Faunal Analysis of Tiktalik.” *Arctic Anthropology* 50 (2): 4–24.](http://paperpile.com/b/GftuPk/Wfvug)

[Morey, Darcy F. 2010. *Dogs: Domestication and the Development of a Social Bond*. Cambridge University Press.](http://paperpile.com/b/GftuPk/9nIm2)

[Morey, Darcy F., and Kim Aaris-Sørensen. 2002. “Paleoeskimo Dogs of the Eastern Arctic.” *Arctic* 55 (1): 44–56.](http://paperpile.com/b/GftuPk/9kaOs)

[Morgan, E. D., L. Titus, R. J. I. Small, and Corony Edwards. 1983. “The Composition of Fatty Materials from a Thule Eskimo Site on Herschel Island.” *Arctic* 36 (4): 356–60.](http://paperpile.com/b/GftuPk/gT1fP)

[Morrison, David. 1990. *Iglulualumiut Prehistory: The Lost Inuit of Franklin Bay*. Archaeological Survey of Canada Mercury Series Paper 116. Hull, Quebec.: Canadian Museum of Civilization.](http://paperpile.com/b/GftuPk/St2Xq)

[———. 2000. “Inuvialuit Fishing and the Gutchiak Site.” *Arctic Anthropology* 37 (1): 1–42.](http://paperpile.com/b/GftuPk/bCJ0d)

[Morrison, David A. 1983. *Thule Culture in Western Coronation Gulf, N.W.T*. Vol. Archaeological Surey of Canada Paper 116,. National Museum of Man Mercury Series. . Ottawa: National Museums of Canada.](http://paperpile.com/b/GftuPk/dtiX6)

[Moss, Madonna. 2004. *Archaeological Investigation of Cape Addington Rockshelter: Human Occupation of the Rugged Seacoast on the Outer Prince of Wales Archipelago, Alaska*. University of Oregon Anthropological Paper No. 63. University of Oregon, Department of Anthropology.](http://paperpile.com/b/GftuPk/VOZat)

[Moss, Madonna, Justin M. Hays, Peter Bowers, and Douglas R. Reger. 2016. *The Archaeology of Coffman Cove: 5500 Years of Settlement in the Heart of Southeast Alaska*. University of Oregon Museum of Natural and Cultural History and the Department of Anthropology, University of Oregon.](http://paperpile.com/b/GftuPk/3A67b)

[Murray, Maribeth S. 1996. “Economic Change in the Palaeoeskimo Prehistory of the Foxe Basin, NWT.”](http://paperpile.com/b/GftuPk/g3MH1) <https://macsphere.mcmaster.ca/handle/11375/12973>[.](http://paperpile.com/b/GftuPk/g3MH1)

[Murray, Maribeth Suzanne. 2005. “Prehistoric Use of Ringed Seals: A Zooarchaeological Study from Arctic Canada.” *Environmental Archaeology* 10 (1): 19–38.](http://paperpile.com/b/GftuPk/R22x6)

[Nash, R. J. 1976. “Cultural Systems and Culture Change in the Central Arctic.” *Memoirs of the Society for American Archaeology,* 31: 150–55.](http://paperpile.com/b/GftuPk/XN97S)

[Nash, Ronald J. 1972. “Dorset Culture in Northeastern Manitoba, Canada.” *Arctic Anthropology* 9 (1): 10–16.](http://paperpile.com/b/GftuPk/Wtov4)

[Natcher, David C., and Andrea H. Procter. 2012. *Settlement, Subsistence, and Change Among the Labrador Inuit: The Nunatsiavummiut Experience*. Univ. of Manitoba Press.](http://paperpile.com/b/GftuPk/4haOA)

[Nikitin, Iu G., and S. Chzhun. 2009. *Arkheologicheskie Issledovaniia Na Poselenii Cherniatino 2 v Primor’e v 2008 Gody*. Vol. 1-2. Tedzhon, DVGTU, IIAiE DVO RAN, Vladivostok.](http://paperpile.com/b/GftuPk/e4nUI)

[Nikitin, Iu.G., Chzhun, S., Chzho, Li Ch. 2008. *Arkheologicheskie Issledovaniia Na Poselenii Cherniatino 2 v Primor’e v 2007 Gody*. Vol. 1-2. Chunnam Vuekyn, DVGTU, IIAiE DVO RAN, Vladivostok.](http://paperpile.com/b/GftuPk/JXTS1)

[Ní Leathlobhair, Máire, Angela R. Perri, Evan K. Irving-Pease, Kelsey E. Witt, Anna Linderholm, James Haile, Ophelie Lebrasseur, et al. 2018. “The Evolutionary History of Dogs in the Americas.” *Science*, no. 361: 81–85.](http://paperpile.com/b/GftuPk/VwOGC)

[Nomokonova, Tatiana, Robert J. Losey, Ol’ga I. Goriunova, and Andrzej W. Weber. 2013. “A Freshwater Old Carbon Offset in Lake Baikal, Siberia and Problems with the Radiocarbon Dating of Archaeological Sediments: Evidence from the Sagan-Zaba II Site.” *Quaternary International: The Journal of the International Union for Quaternary Research* 290-291 (March): 110–25.](http://paperpile.com/b/GftuPk/j6BzW)

[Nomokonova, T. Iu, and O. I. Goriunova. 2013. “Kosti Zhivotnykh Iz Kammenykh Konstruktsii Bukhty Sagan-Nuge Na Malom More Oz. Baikal [Animal Bones from Stone Features of Sagan-Nuge Cove in the Little Sea Region of Lake Baikal].” *Bulletin of the Irkutsk State University. Geoarchaeology, Ethnology, and Anthropology* Series 1(2): 44–61.](http://paperpile.com/b/GftuPk/6FlcP)

[Norman, Lauren E. Y., T. Max Friesen, Claire Alix, Michael J. E. O’Rourke, and Owen K. Mason. 2017. “An Early Inupiaq Occupation: Observations on a Thule House From Cape Espenberg, Alaska.” *Open Archaeology* 3 (1): 57.](http://paperpile.com/b/GftuPk/TKoet)

[Ólafsson, G. 2010. “Bessastaðarannsókn 1987: Aðdragandi Og Upphaf - Uppgraftarsvæði 1-11 (Skýrslur Þjóðminjasafns Íslands No. 2010/1).” *Reykjavík: Þjóðminjasafn Íslands.*](http://paperpile.com/b/GftuPk/3gxAn)

[———. 2013. “Bessastaðarannsókn II: Kirkjugarður Og Miðaldaminjar, Uppgraftarsvæði 12-15 (Skýrslur Þjóðminjasafns Íslands No. 2013/2).” *Reykjavík: Þjóðminjasafn Íslands.*](http://paperpile.com/b/GftuPk/Suvit)

[Oswalt, Wendell. 1952. “The Archaeology of Hooper Bay Village, Alaska.” *Anthropological Papers of the University of Alaska* 1 (1): 47–91.](http://paperpile.com/b/GftuPk/z1v7k)

[Pálsdóttir, A. H. 2010. “Dýrabein Frá Alþingisreit: Greining á Dýrabeinum Frá Svæðum A, B Og C (Skýrslur Íslenskra Fornleifarannsókna Ehf. No. 2010–1).” *Reykjavík: Íslenskar Fornleifarannsóknir Ehf.*](http://paperpile.com/b/GftuPk/rPiFF)

[———. 2013. “Dýrabeinin Frá Alþingisreit IV. Fasi (871-1226): Uppgröftur 2008-2012 (Skýrslur Íslenskra Fornleifarannsókna Ehf. No. 2013–1).” *Reykjavík: Íslenskar Fornleifarannsóknir Ehf.*](http://paperpile.com/b/GftuPk/L4PAb)

[Pálsdóttir, A. H., S. Boessenkool, and B. Star. 2018. “Ancient DNA Sampling Report: Walrus Bones from Alþingisreitur (Technical Report No. 100).” *Reykjavík: Landbúnaðarháskóli Íslands.*](http://paperpile.com/b/GftuPk/4rNQM)

[Pang, Jun-Feng, Cornelya Kluetsch, Xiao-Ju Zou, Ai-Bing Zhang, Li-Yang Luo, Helen Angleby, Arman Ardalan, et al. 2009. “mtDNA Data Indicate a Single Origin for Dogs South of Yangtze River, Less than 16,300 Years Ago, from Numerous Wolves.” *Molecular Biology and Evolution* 26 (12): 2849–64.](http://paperpile.com/b/GftuPk/FegVc)

[Paradis, E., J. Claude, and K. Strimmer. 2004. “APE: Analyses of Phylogenetics and Evolution in R Language.” *Bioinformatics*  20 (2): 289–90.](http://paperpile.com/b/GftuPk/U9xiu)

[Paradis, Emmanuel, Julien Claude, and Korbinian Strimmer. 2004. “APE: Analyses of Phylogenetics and Evolution in R Language.” *Bioinformatics*  20 (2): 289–90.](http://paperpile.com/b/GftuPk/tnmEq)

[Park, Robert W. 1987. “Dog Remains from Devon Island, NWT: Archaeological and Osteological Evidence for Domestic Dog Use in the Thule Culture.” *Arctic*, 184–90.](http://paperpile.com/b/GftuPk/uEuYq)

[———. 1993. “The Dorset-Thule Succession in Arctic North America : Assessing Claims for Culture Contact Author ( S ): Robert W . Park Published by : Society for American Archaeology Stable URL : http://www.jstor.org/stable/281966 Http://www.jstor.org/page/info/about/p” 58 (2): 203–34.](http://paperpile.com/b/GftuPk/0Ngo5)

[Patterson, Nick, Priya Moorjani, Yontao Luo, Swapan Mallick, Nadin Rohland, Yiping Zhan, Teri Genschoreck, Teresa Webster, and David Reich. 2012. “Ancient Admixture in Human History.” *Genetics* 192 (3): 1065–93.](http://paperpile.com/b/GftuPk/gSD4i)

[Patterson, Nick, Alkes L. Price, and David Reich. 2006. “Population Structure and Eigenanalysis.” *PLoS Genetics* 2 (12): e190.](http://paperpile.com/b/GftuPk/EHUqt)

[Popov, Alexander N., Andrei V. Tabarev, and Yuri A. Mikishin. 2014. “Neolithization and Ancient Landscapes in Southern Primorye, Russian Far East.” *Journal of World Prehistory* 27 (3-4): 247–61.](http://paperpile.com/b/GftuPk/nr4Rl)

[Popov, A. N., N. F. Chikisheva, and E. G. Shpakova. 1997. “Boismanskaya Arheologicheskaya Kultura v Yuzhnom Primorye.(Boisman Archaeological Culture in Southern Primorye). Novosibirsk: Institute of Archaeology and Ethnography.”](http://paperpile.com/b/GftuPk/RJ9NW)

[Popov, A. N., and B. V. Lazin. 2011. “Arkheologicheskie Issledovaniia Na Ostrove Russkom v G. Vladivostoke v 2010-2011 Godakh.” In *Drevnosti Po Obe Storony Velikogo Okeana,* edited by D. L. Brodianskii, 118–26. DVGTU, Vladivostok.](http://paperpile.com/b/GftuPk/NoHk2)

[Purcell, Shaun, Benjamin Neale, Kathe Todd-Brown, Lori Thomas, Manuel A. R. Ferreira, David Bender, Julian Maller, et al. 2007. “PLINK: A Tool Set for Whole-Genome Association and Population-Based Linkage Analyses.” *American Journal of Human Genetics* 81 (3): 559–75.](http://paperpile.com/b/GftuPk/yC722)

[Raghavan, M., M. DeGiorgio, A. Albrechtsen, I. Moltke, P. Skoglund, T. S. Korneliussen, B. Gronnow, et al. 2014. “The Genetic Prehistory of the New World Arctic.” *Science* 345 (6200): 1255832–1255832.](http://paperpile.com/b/GftuPk/y69GU)

[Rainey, Froelich G. 1941. “Eskimo Prehistory: The Orvik Site on the Punuk Islands.” *Anthropological Papers of the American Museum of Natural History*.](http://paperpile.com/b/GftuPk/jpSvK)

[Rakov, V. A., A. N. Popov, L. E. Vasil’eva, Iu V. Zavertanova, and Iu A. }. Mikishin. 2009. “Fauna Pribrezhnoi Zony Proliva Bosfor-Vostochnyi Perioda Zheleznogo Veka (po Materialan Spasatel’nykh Raskopok Pamiatnikov Nazimova-1 I Pospelova-1 v G. Vladivostoke).” In *Ot Mongolii Do Primor’ia I Sakhalina*, edited by D. L. Brodianskii, 162–212. DVGTU, Vladivostok.](http://paperpile.com/b/GftuPk/de440)

[Rankin, L. 2014. “Indian Harbour, Norman’s Island and Double Mer Point, Labrador.” *Provincial Archaeology Office 2013 Archaeology Review St. John’s, NL*.](http://paperpile.com/b/GftuPk/syJ9e)

[R Core Team. 2018. *R: A Language and Environment for Statistical Computing*. Vienna, Austria: R Foundation for Statistical Computing.](http://paperpile.com/b/GftuPk/35fIo)

[Reger, D. R., Madonna L. Moss, Peter M. Bowers, and Justin M. Hays. 2017. *Recovery of Archaeological Data from the Ferry Terminal Site (49-PET-556), Coffman Cove, Alaska*. Preliminary Report Submitted to the USDA Forest Service, December 2017. Fairbanks, AK: Northern Land Use Research.](http://paperpile.com/b/GftuPk/oA8rT)

[Reimer, Paula J., Edouard Bard, Alex Bayliss, J. Warren Beck, Paul G. Blackwell, Christopher Bronk Ramsey, Caitlin E. Buck, et al. 2013. “IntCal13 and Marine13 Radiocarbon Age Calibration Curves 0–50,000 Years Cal BP.” *Radiocarbon* 55 (4): 1869–87.](http://paperpile.com/b/GftuPk/nX6QW)

[Renouf, M. A. P. 1993. “Palaeoeskimo Seal Hunters at Port Au Choix, Northwestern Newfoundland.” *Newfoundland and Labrador Studies*.](http://paperpile.com/b/GftuPk/PYFRK) <https://journals.lib.unb.ca/index.php/NFLDS/article/viewFile/941/1294>[.](http://paperpile.com/b/GftuPk/PYFRK)

[Rick, Anne Meachem. 1980. “Non-Cetacean Vertebrate Remains from Two Thule Winter Houses on Somerset Island, NWT.” *Canadian Journal of Archaeology/Journal Canadien d’Archéologie*, 99–117.](http://paperpile.com/b/GftuPk/6eg5N)

[Riewe, R., Suluk, L., & Brandon, L. 1989. “Inuit Land Use and Occupancy in Northern Manitoba.” *Northern Review* 3/4.](http://paperpile.com/b/GftuPk/iOaum) <http://journals.sfu.ca/nr/index.php/nr/article/view/458>[.](http://paperpile.com/b/GftuPk/iOaum)

[Roberts, H. M. 2009. “Gásir Post Excavation Reports-.” *Fornleifastofnun Íslands, Reykjavík.* 1 (FS423-010712,).](http://paperpile.com/b/GftuPk/hgdsk)

[Rohland, Nadin, and Michael Hofreiter. 2007. “Ancient DNA Extraction from Bones and Teeth.” *Nature Protocols* 2 (7): 1756–62.](http://paperpile.com/b/GftuPk/sGaW6)

[Rohland, Nadin, Heike Siedel, and Michael Hofreiter. 2010. “A Rapid Column-Based Ancient DNA Extraction Method for Increased Sample Throughput.” *Molecular Ecology Resources* 10 (4): 677–83.](http://paperpile.com/b/GftuPk/2uNo6)

[Rohlf, F. J. 2004. “TpsDig, Version 1.4.” *Http://life.bio.sunysb.edu/morph/*.](http://paperpile.com/b/GftuPk/7r5CD) <https://ci.nii.ac.jp/naid/20001496718/>[.](http://paperpile.com/b/GftuPk/7r5CD)

[Rohlf, F. James, and Dennis Slice. 1990. “Extensions of the Procrustes Method for the Optimal Superimposition of Landmarks.” *Systematic Biology* 39 (1): 40–59.](http://paperpile.com/b/GftuPk/MvJjy)

[Ross, W. Gillies. 1977. “Whaling and the Decline of Native Populations [Corrected Title: Intoduced Disease and Eskimo Mortality during the Whaling Period: The Extinction of the Saglermiut in Hudson Bay, 1902].” *Arctic Anthropology* 14 (2): 1–8.](http://paperpile.com/b/GftuPk/sPjVz)

[Ryan, Karen, and Janet Young. 2013. “Identification of a Probable Aarnguaq in a Sadlermiut Grave from Native Point, Southampton Island, Nunavut, Canada.” *Arctic Anthropology* 50 (1): 20–48.](http://paperpile.com/b/GftuPk/KCAIx)

[Sabo, George, and John D. Jacobs. 1980. “Aspects of Thule Culture Adaptations in Southern Baffin Island.” *Arctic* 33 (3): 487–504.](http://paperpile.com/b/GftuPk/H34Pm)

[Sampson, P. D., F. L. Bookstein, F. H. Sheenan, and E. L. Bolson. 1996. “Eigenshape Analysis of Left Ventricular Outlines from Contrast Ventriculograms.” In *Advances in Morphometrics*, edited by L. F. Marcus, M. Corti, A. Loy, G. J. P. Naylar, and D. E. Slice, 211–33. NATO ASI Series A: Life Sciences. New York: Plenum.](http://paperpile.com/b/GftuPk/514TD)

[Savolainen, Peter, Thomas Leitner, Alan N. Wilton, Elizabeth Matisoo-Smith, and Joakim Lundeberg. 2004. “A Detailed Picture of the Origin of the Australian Dingo, Obtained from the Study of Mitochondrial DNA.” *Proceedings of the National Academy of Sciences of the United States of America* 101 (33): 12387–90.](http://paperpile.com/b/GftuPk/9PizQ)

[Savolainen, Peter, Ya-Ping Zhang, Jing Luo, Joakim Lundeberg, and Thomas Leitner. 2002. “Genetic Evidence for an East Asian Origin of Domestic Dogs.” *Science* 298 (5598): 1610–13.](http://paperpile.com/b/GftuPk/r08QL)

[Schlager, S. 2017. “Morpho and Rvcg – Shape Analysis in R.” In *Statistical Shape and Deformation Analysis*, edited by G. Zheng, S. Li, and G. Szekely, 217–56.](http://paperpile.com/b/GftuPk/FTCeR)

[Schledermann, Peter. 1990. *Crossroads to Greenland: 3000 Years of Prehistory in the Eastern High Arctic*. Arctic Institute of North America, the University of Calgary.](http://paperpile.com/b/GftuPk/Bm9uR)

[Schledermann, Peter, and Karen M. McCullough. 2003. “Late Thule Culture Developments on the Central East Coast of Ellesmere Island.” *Sila – The Greenland Research Centre at the National Museum of Denmark and Danish Polar Center, Copenhagen.*](http://paperpile.com/b/GftuPk/rQxj4)

[Schulting, Rick J., Christopher Bronk Ramsey, Vladimir I. Bazaliiskii, Olga I. Goriunova, and Andrzej Weber. 2014. “Freshwater Reservoir Offsets Investigated Through Paired Human-Faunal 14C Dating and Stable Carbon and Nitrogen Isotope Analysis at Lake Baikal, Siberia.” *Radiocarbon* 56 (3): 991–1008.](http://paperpile.com/b/GftuPk/e7klX)

[Sheehan, Glenn W. 1997. *In the Belly of the Whale: Trade and War in Eskimo Society*. Vol. 6. Alaska Anthropological Association.](http://paperpile.com/b/GftuPk/mlOMD)

[Shirar, Scott. 2011. “Late Holocene Chronology of the Noatak and Kobuk Rivers.” *Alaska Journal of Anthropology*, 1.](http://paperpile.com/b/GftuPk/zwsRy)

[Sloan, Anna C. 2014. “Spirituality and the Seamstress: Birds in Ipiutak and Western Thule Lifeways at Deering, Alaska.” *Arctic Anthropology* 51 (2): 35–59.](http://paperpile.com/b/GftuPk/720EY)

[Smiarowski, Konrad. 2012. “E172 Tatsip Ataa Midden Excavation 2009 & 2010 Preliminary Excavation Report.” *http://www.nabohome.org/publications/ipy/E172ReportDraft3KS3-20-12.pdf*.](http://paperpile.com/b/GftuPk/ji8X8)

[Sørensen, Mikkel, and Hans Christian Gulløv. 2012. “The Prehistory of Inuit in Northeast Greenland.” *Arctic Anthropology* 49 (1): 88–104.](http://paperpile.com/b/GftuPk/aRAdo)

[Staab, M. L. 1979. “Analysis of Faunal Material Recovered from a Thule Eskimo Site on the Island of Silumiut, N.W.T., Canada.” In *The Thule Eskimo Culture: An Anthropological Retrospective.*, edited by A. P. McCartney, 349–79. Mercury Series. Archaeological Survey of Canada Paper 88. Ottawa: National Museum of Man.](http://paperpile.com/b/GftuPk/PhHnt)

[Stamatakis, Alexandros. 2006. “RAxML-VI-HPC: Maximum Likelihood-Based Phylogenetic Analyses with Thousands of Taxa and Mixed Models.” *Bioinformatics*  22 (21): 2688–90.](http://paperpile.com/b/GftuPk/mZFqc)

[———. 2014. “RAxML Version 8: A Tool for Phylogenetic Analysis and Post-Analysis of Large Phylogenies.” *Bioinformatics*  30 (9): 1312–13.](http://paperpile.com/b/GftuPk/nfe8C)

[Steffian, Amy F. 1992. “Fifty Years after Hrdlicka: Further Excavation of the Uyak Site, Kodiak Island, Alaska.” *Contributions to the Anthropology of Southcentral and Southwestern Alaska*, 141–64.](http://paperpile.com/b/GftuPk/EF8Q5)

[Stenton, Douglas R. 1987. “Recent Archaeological Investigations in Frobisher Bay, Baffin Island, NWT.” *Canadian Journal of Archaeology/Journal Canadien d’Archéologie*, 13–48.](http://paperpile.com/b/GftuPk/KEL0u)

[Stuiver, M., P. J. Reimer, and R. W. Reimer. 2019. “CALIB 7.1.” *Http://calib.org*.](http://paperpile.com/b/GftuPk/4QrBn)

[Sutherland, Patricia D. 1996. “Continuity and Change in the Paleo-Eskimo Prehistory of Northern Ellesmere Island.” *The Paleo-Eskimo Cultures of Greenland: New Perspectives in Greenlandic Archaeology. Publication*, no. 1: 271–94.](http://paperpile.com/b/GftuPk/jslUe)

[Tackney, Justin, Anne M. Jensen, Caroline Kisielinski, and Dennis H. O’Rourke. 2019. “Molecular Analysis of an Ancient Thule Population at Nuvuk, Point Barrow, Alaska.” *American Journal of Physical Anthropology* 168 (2): 303–17.](http://paperpile.com/b/GftuPk/u5yb9)

[Thai, Quan Ke, Dung Anh Chung, and Hoang-Dung Tran. 2017. “Canis mtDNA HV1 Database: A Web-Based Tool for Collecting and Surveying Canis mtDNA HV1 Haplotype in Public Database.” *BMC Genetics* 18 (1): 60.](http://paperpile.com/b/GftuPk/GVPHf)

[Thalmann, O., B. Shapiro, P. Cui, V. J. Schuenemann, S. K. Sawyer, D. L. Greenfield, M. B. Germonpré, et al. 2013. “Complete Mitochondrial Genomes of Ancient Canids Suggest a European Origin of Domestic Dogs.” *Science* 342 (6160): 871–74.](http://paperpile.com/b/GftuPk/Oq0dQ)

[Thostrup, Christian Bendix. 1911. *Ethnographic Description of the Eskimo Settlements and Stone Remains in North-East Greenland*. Vol. 4. CA Reitzel.](http://paperpile.com/b/GftuPk/Qljqu)

[Tremayne, Andrew H., Christyann M. Darwent, John Darwent, Kelly A. Eldridge, and Jeffrey T. Rasic. 2018. “Iyatayet Revisited: A Report on Renewed Investigations of a Stratified Middle-to-Late Holocene Coastal Campsite in Norton Sound, Alaska.” *Arctic Anthropology* 55 (1): 1–23.](http://paperpile.com/b/GftuPk/7VdTG)

[VanStone, James W., and Charles V. Lucier. 1974. “An Early Archaeological Example of Tattooing from Northwestern Alaska.” *Fieldiana. Anthropology* 66 (1): 1–9.](http://paperpile.com/b/GftuPk/Gvk1t)

[Vésteinsson, Orri. 2014. “Archaeological Investigations in Igaliku: Excavations in the Meadow, 2012–2013. Report FS517-12151.” *Fornleifastofnun Íslands Institute of Archaeology, Reykjavík.*](http://paperpile.com/b/GftuPk/yPtNj)

[Woollett, James M. 2003. “An Historical Ecology of Labrador Inuit Culture Change.” City University of New York.](http://paperpile.com/b/GftuPk/hqbK2)

[———. 2007. “Labrador Inuit Subsistence in the Context of Environmental Change: An Initial Landscape History Perspective.” *American Anthropologist* 109 (1): 69–84.](http://paperpile.com/b/GftuPk/7eaAW)

[Workman, Karen W. 1975. “Chugachik Island (SEL 033): Test Excavations at a Middle Kachemak Tradition Site in Kachemak Bay, Alaska, July 1974.” *Prepared for Alaska Division of Parks.*](http://paperpile.com/b/GftuPk/xTWq8)

[———. 1978. “The Second Season at Chugachik Island (SEL 033), Kachemak Bay, Alaska.” In *Paper Presented at the 5th Annual Meeting of the Alaska Anthropological Association, March 17-18, Anchorage.*](http://paperpile.com/b/GftuPk/lv6U3)

[Workman, William B. 1998. “Archaeology of the Southern Kenai Peninsula.” *Arctic Anthropology* 35 (1): 146–59.](http://paperpile.com/b/GftuPk/Gazln)

[Workman, William B., Lobdell John E., and Karen W. Workman. 1980. “Recent Archeological Work in Kachemak Bay, Gulf of Alaska.” *Arctic* 33 (3): 385–99.](http://paperpile.com/b/GftuPk/dTE2O)

[Workman, William B., and Peter Zollars. 2002. “The Dispersal of the Arctic Small Tool Tradition into Southern Alaska: Dates and Data from the Kenai Peninsula, Southcentral Alaska.” *Anthropological Papers of the University of Alaska New Series* 2 (1): 39–49.](http://paperpile.com/b/GftuPk/oxhZ7)

[Yorga, Brian W. D. 1980. “Washout: A Western Thule Site on Herschel Island, Yukon Territory.” *Musée National de l’Homme. Collection Mercure. Commission Archéologique Du Canada. Publications d'Archéologie. Dossier Ottawa*, no. 98: 1–201.](http://paperpile.com/b/GftuPk/czw1j)

[Zelditch, Miriam Leah, Donald L. Swiderski, H. David Sheets, and William L. Fink. 2004. *Geometric Morphometrics for Biologists: A Primer*. Elsevier.](http://paperpile.com/b/GftuPk/tcjWm)

[Zollars, Peter. 1982. “Chugachik Island Project Report, 1982.” *Prepared for the Otto Geist Fund Committee, University of Alaska Museum.*](http://paperpile.com/b/GftuPk/3GmWB)
