# Supplementary material for: Specialized sledge dogs accompanied Inuit dispersal across the North American Arctic
Source: Proc Biol Sci. 2019 Nov 27;286(1916):20191929. doi: 10.1098/rspb.2019.1929 (PMC6939252; doi:10.1098/rspb.2019.1929)
Supplement: Supplementary captions for Figures S1 - S17 and captions for Databases S1 to S6 [file rspb20191929supp2.docx]

## SI Text : Cultural, archaeological and methodological supplementary information

## SI Figure Captions

**Fig. S1:** MapDamage plots showing general patterns of deamination seen in samples included in the study with transition frequency plotted against position in read. Deaminated bases appear most frequently at the ends of reads, the frequency of the appearance of deaminated bases increases between younger and older samples. A) Plot of historical sample collected ca. 135 years before present. B) Plot of Thule Inuit sample, ca. 600 years old. C) Plot of Siberian Holocene sample, ca. 6,000 years old.

**Fig. S2**: Maximum likelihood tree generated with RAXML with 1,000 bootstraps from samples with a minimum mean of 10x read depth of 95\% of sites in the mitochondrial genome with the exclusion of samples in basal positions of the subclades to ensure robusticity of node support.

**Fig. S3:** Bayesian skyline plot of whole dataset, plot displates Ne against time (years before present). The black line represents the average Ne and the 95\% HPD is depicted with the purple area. Bayesian skyline plot of all samples with a minimum mean of 10x read depth, generated with BEAST2.4.

**Fig. S4:** Maximum likelihood tree generated with RAXML with 1,000 bootstraps from samples with a minimum mean of 3x read depth of 80\% of positions in the mitochondrial genome. Arrows indicating A-clade subclades and X-clade. Three specimen (coyote outgroup and two Chinese wolves) were excluded from figure for better visualisation, remaining samples were coloured according to cultural affiliation. Dog clades B-E are collapsed into black clades with labels in red.

**Fig. S5:** Maximum likelihood tree generated with RAXML with 1,000 bootstraps from samples with a minimum mean of 3x read depth of 80\% of positions in the mitochondrial genome. Arrows indicating A-clade subclades and X-clade. The outgroup and Chinese wolves are included in figure.

**Fig. S6:** Maximum likelihood tree generated with RAXML with 1,000 bootstraps from samples with a minimum mean of 10x read depth of 95\% of sites in the mitochondrial genome. A-clade subclades and X-clade are indicated with arrows.

**Fig. S7:** Bayesian mitochondrial phylogeny constructed with BEAST2.4 for A-clade with dated nodes from samples with a minimum mean of 10x read depth. The subclades of the A-clade have been coloured according with Fig. 2, purple for clade A2b, blue for clade A2a, red for clade A1b, and yellow for clade A1a.

**Fig. S8:** Bayesian mitochondrial phylogeny constructed with BEAST2.4 for whole dataset with dated nodes from samples with a minimum mean of 10x read depth. The subclades of the A-clade have been coloured according with Fig. 2, purple for clade A2b, blue for clade A2a, red for clade A1b, and yellow for clade A1a.

**Fig. S9:** Bayesian skyline plots corresponding to Inuit and Historical/Modern sample sets from the North American Arctic. Each plot displates Ne against from 15,000 years ago to present. The black line represents the average Ne and the 95\% HPD is depicted with the purple area. A) North American Arctic dogs from 300 years ago until 50 years before present. B) North American Arctic dogs from 300 years ago until present. C) Samples of only North American dogs from Inuit contexts. D) Combined dataset of Inuit dogs until modern dogs in the North American Arctic.

**Fig. S10:** Nuclear ancestry analyses of AL2797 (Thule sample from Nunalleq). A-B. Principal component analysis of nuclear SNP data with (A) and without (B) coyotes genomes. This data set comprises 49 canids genomes (see Table X for accession numbers and sample provenance), including an outgroup (Andean fox) 2 coyotes (C\_MidW, C\_Cal), 10 modern Arctic, pre-contact dogs and CTVT genomes (D\_AHusky91, D\_Green, D\_Husky, D\_Husky89, D\_Mal68, AL3194, AL3223, C\_399T, C\_79T, C\_24T), 9 modern East Asian dogs (D\_China8, D\_Tibet3, D\_Tibet4, D\_TMastif4, D\_TMastif5, D\_China9, D\_Dingo, D\_Viet21, D\_Viet59) and 17 Western dogs (D\_India168, D\_India60, D\_SLaika, D\_Na89, D\_Basenji, D\_Na8, D\_Qatar27, D\_Qatar5, D\_NGDG, D\_Peru, D\_Port71, D\_Mex, D\_Port61, D\_Leb85, D\_Leb79, D\_GerShep6, D\_GerShep3), 6 modern Eurasian wolves (W\_Iran, W\_India, W\_Spa, W\_Port, W\_Mongo, W\_Altai), 1 ancient Eurasian wolf (TAI) and 3 modern American wolves (W\_Mex1, W\_Yellow1, W\_Yellow2). C. Neighbour joining tree tree depicting the relationship between these genomes D. D-statistics of the form D(AndeanFox, AL2797, Pop3, Pop4) where Pop3 was fixed as either Western dogs, Asian dogs, Arctic dogs, or CTVT (coloured box plot) and Pop4 represents the genome on the y-axis.

**Fig. S11:** Estimated *F*_ST_ and statistical significance were computed using absolute haplogroup frequencies (i.e., sample sizes were accounted for) in Arlequin 3.5.1.3. *F*_ST_ ranges from 0 to 1 with 0 reflecting no difference in haplogroup frequencies and 1 reflecting the greatest possible difference. Statistical significance was denoted by asterisks (*** P < 0.001, *P < 0.05).

**Fig. S12:** A) A median joining network of historical and modern haplotypes from North American Arctic dogs constructed using Network v5.0.1.0., illustrating that subclades A2a and A2b are more deeply rooted than subclades A1a or A1b; rho estimates indicate the average number of mutations accumulating since divergence from the root haplotype (too few samples from A2b were present in historical dogs to estimate rho). B) The relatively recent derivation of A1a and A1b haplotypes (past ~200 years) relative to A2a haplotypes with ages based on calibration to the North American A2a subclade assuming it began accumulating the observed mutations at least 1,000 years ago (rho minus 1 SD = 1000 years), coinciding with the Inuit expansion.

**Fig. S13:** Maps showing the sampling location and overall sample sizes for the three elements analysed using GMM methods.

**Fig. S14:** (top) Images of the canid crania showing the placement of the 30 permanent 3D landmarks. 2D landmark configurations for the (middle) the lower M1 showing permanent landmarks (red) and 2 curves of sliding semi-landmarks (grey) and (bottom) mandible showing 15 permanent landmarks (red).

**Fig. S15** Two first axes of Principal component analyses of the Crania (top), lower M1 (middle), and mandible (bottom).

**Fig. S16** Two first axes of Canonical Variate Analyses of the Crania (top), lower M1 (middle), and mandible (bottom).

**Fig. S17:** Bone collagen $\delta$^13^C and $\delta$^15^N data for the canids from the Nunalleq Site. The wolf is labelled (AL2797), and shows a diet with less marine protein than the dogs from the site.

## Captions for Databases S1 – S6

**Table S1:** Data table listing all specimens used in for DNA analysis, including group assignment, type of element analysed, and sample collection location. (Excel)

**Table S2:** Data table listing all published mitochondrial sequences used as a reference panel. (Excel)

**Table S3:** Data table listing all specimens used for GMM analysis, including group assignment, type of element analysed, and sample collection location. Sheets two to four include the raw coordinates used for the GMM analyses. (Excel)

**Table S4:** Results of carbon and nitrogen stable isotope analysis, including the 7 new samples generated for this analysis, as well as those from [(E. McManus-Fry et al. 2016)](https://paperpile.com/c/GftuPk/FLte6). (Excel)

**Table S5:** Radiocarbon dates generated for this project from Deering, Seward Peninsula, Kotzebue Sound, Alaska; Silumiut site (KkJg-2), northwestern Hudson Bay, Nunavut and Boisman II, Primorsky Siberia. (Excel)

**Table S6**: Nuclear genomes used for the analysis of AL2797 (excel)
