# Supplementary material for: Specialized sledge dogs accompanied Inuit dispersal across the North American Arctic
Source: Proc Biol Sci. 2019 Nov 27;286(1916):20191929. doi: 10.1098/rspb.2019.1929 (PMC6939252; doi:10.1098/rspb.2019.1929)
Supplement: Figs. S1 to S17 [file rspb20191929supp3.pdf]

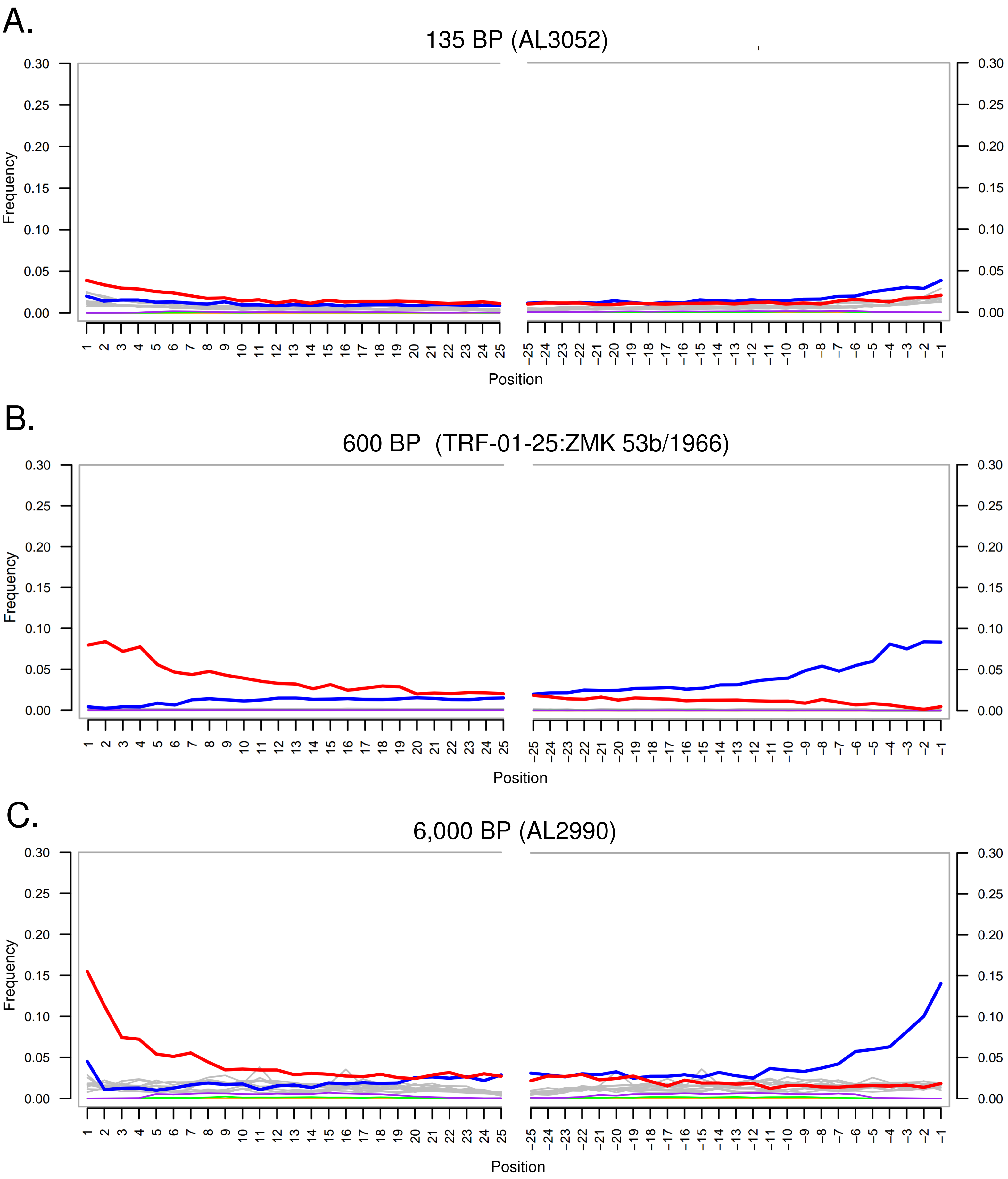
A.
135 BP (AL3052)
B.
600 BP (TRF-01-25:ZMK 53b/1966)
C.
6,000 BP (AL2990)
Frequency
Position
0.00
0.05
0.10
0.15
0.20
0.25
0.30

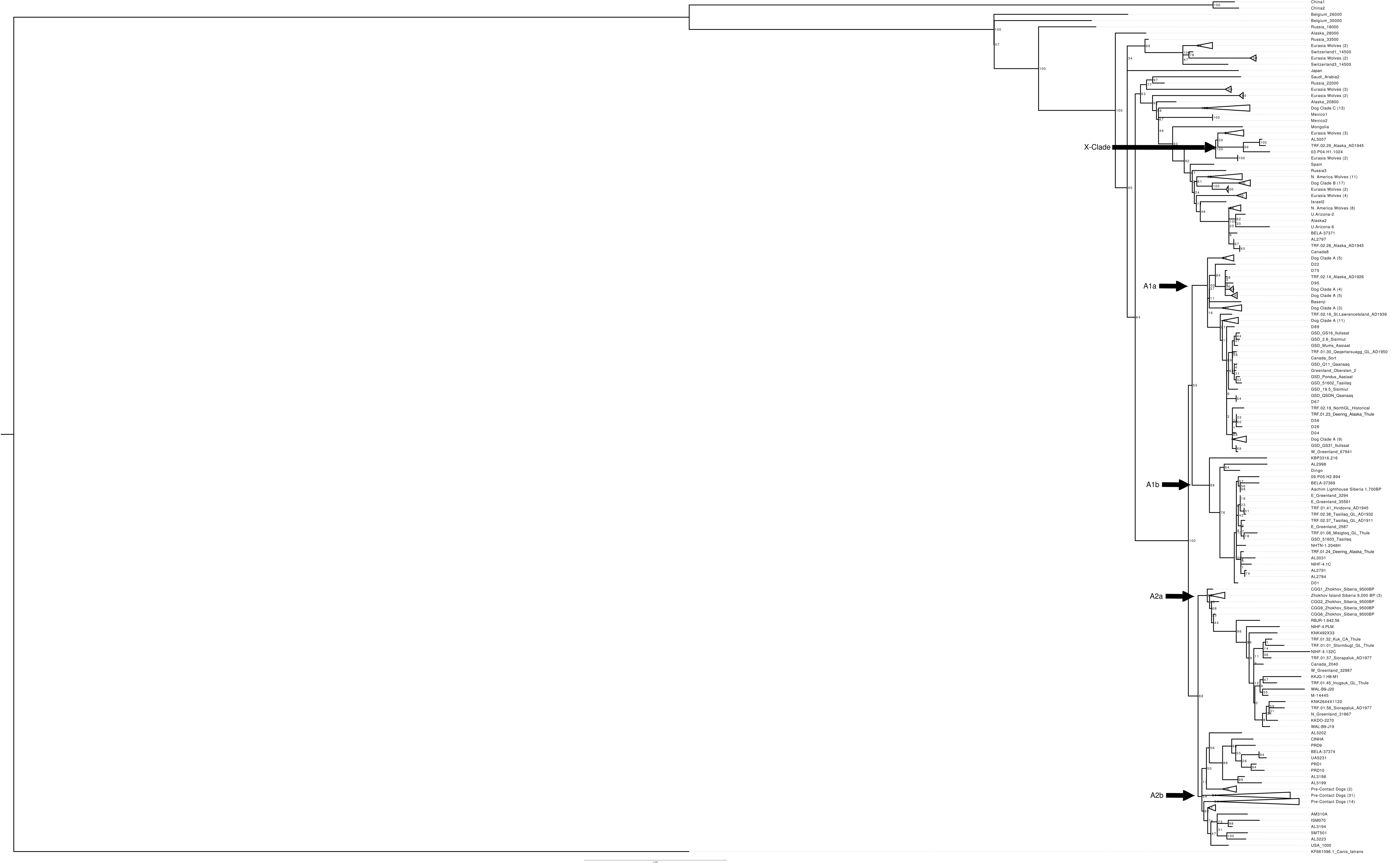
X-Clade
A1a
A1b
A2a
A2b
China1
China2
Belgium_26000
Belgium_30000
Russia_18000
Alaska_28000
Russia_33500
Eurasia Wolves (2)
Switzerland1_14500
Eurasia Wolves (2)
Switzerland3_14500
Japan
Saudi_Arabia2
Russia_22000
Eurasia Wolves (3)
Eurasia Wolves (2)
Alaska_20800
Dog Clade C (13)
Mexico1
Mexico2
Mongolia
Eurasia Wolves (3)
AL3007
TRF.02.29_Alaska_AD1945
03.P04.H1.1024
Eurasia Wolves (2)
Spain
Russia3
N. America Wolves (11)
Dog Clade B (17)
Eurasia Wolves (2)
Eurasia Wolves (4)
Israel2
N. America Wolves (8)
U.Arizona-2
Alaska2
U.Arizona-6
BELA-37371
AL2797
TRF.02.28_Alaska_AD1945
Canada8
Dog Clade A (5)
D22
D75
TRF.02.14_Alaska_AD1926
D95
Dog Clade A (4)
Dog Clade A (5)
Basenji
Dog Clade A (3)
TRF.02.16_St.LawrenceIsland_AD1939
Dog Clade A (11)
D89
GSD_GS16_Ilulissat
GSD_2.6_Sisimiut
GSD_Mums_Aasiaat
TRF.01.30_Qeqertarsuagg_GL_AD1950
Canada_Sort
GSD_Q11_Qaanaaq
Greenland_Obersten_2
GSD_Pondus_Aasiaat
GSD_51602_Tasiilaq
GSD_19.5_Sisimiut
GSD_QSON_Qaanaaq
D67
TRF.02.19_NorthGL_Historical
TRF.01.23_Deering_Alaska_Thule
D56
D28
D04
Dog Clade A (9)
GSD_GS31_Ilulissat
W_Greenland_67941
KBP3316.216
AL2998
Dingo
09.P05.H2.894
BELA-37369
Aachim Lighthouse Siberia 1,700BP
E_Greenland_3294
E_Greenland_35561
TRF.01.41_Hvidovre_AD1945
TRF.02.38_Tasiilaq_GL_AD1932
TRF.02.37_Tasiilaq_GL_AD1911
E_Greenland_2587
TRF.01.08_Misigtoq_GL_Thule
GSD_51603_Tasiilaq
NHTN-1.2048H
TRF.01.24_Deering_Alaska_Thule
AL3031
NIHF-4.1C
AL2791
AL2784
D01
CGG1_Zhokhov_Siberia_9500BP
Zhokhov Island Siberia 9,000 BP (3)
CGG2_Zhokhov_Siberia_9500BP
CGG9_Zhokhov_Siberia_9500BP
CGG6_Zhokhov_Siberia_9500BP
RBJR-1.642.56
NIHF-4.PLM
KNK492X33
TRF.01.32_Kuk_CA_Thule
TRF.01.01_Stormbugt_GL_Thule
NIHF-4.132C
TRF.01.57_Siorapaluk_AD1977
Canada_2040
W_Greenland_32987
KKJG-1.H8-M1
TRF.01.45_Inugsuk_GL_Thule
WAL-B9-J20
M-14445
KNK2644X1120
TRF.01.56_Siorapaluk_AD1977
N_Greenland_31867
KKDO-2270
WAL-B9-J19
AL3202
CINHA
PRD9
BELA-37374
UA5231
PRD1
PRD10
AL3198
AL3199
Pre-Contact Dogs (2)
Pre-Contact Dogs (31)
Pre-Contact Dogs (14)
AM310A
ISM070
AL3194
5MT501
AL3223
USA_1000
KF661096.1_Canis_latrans

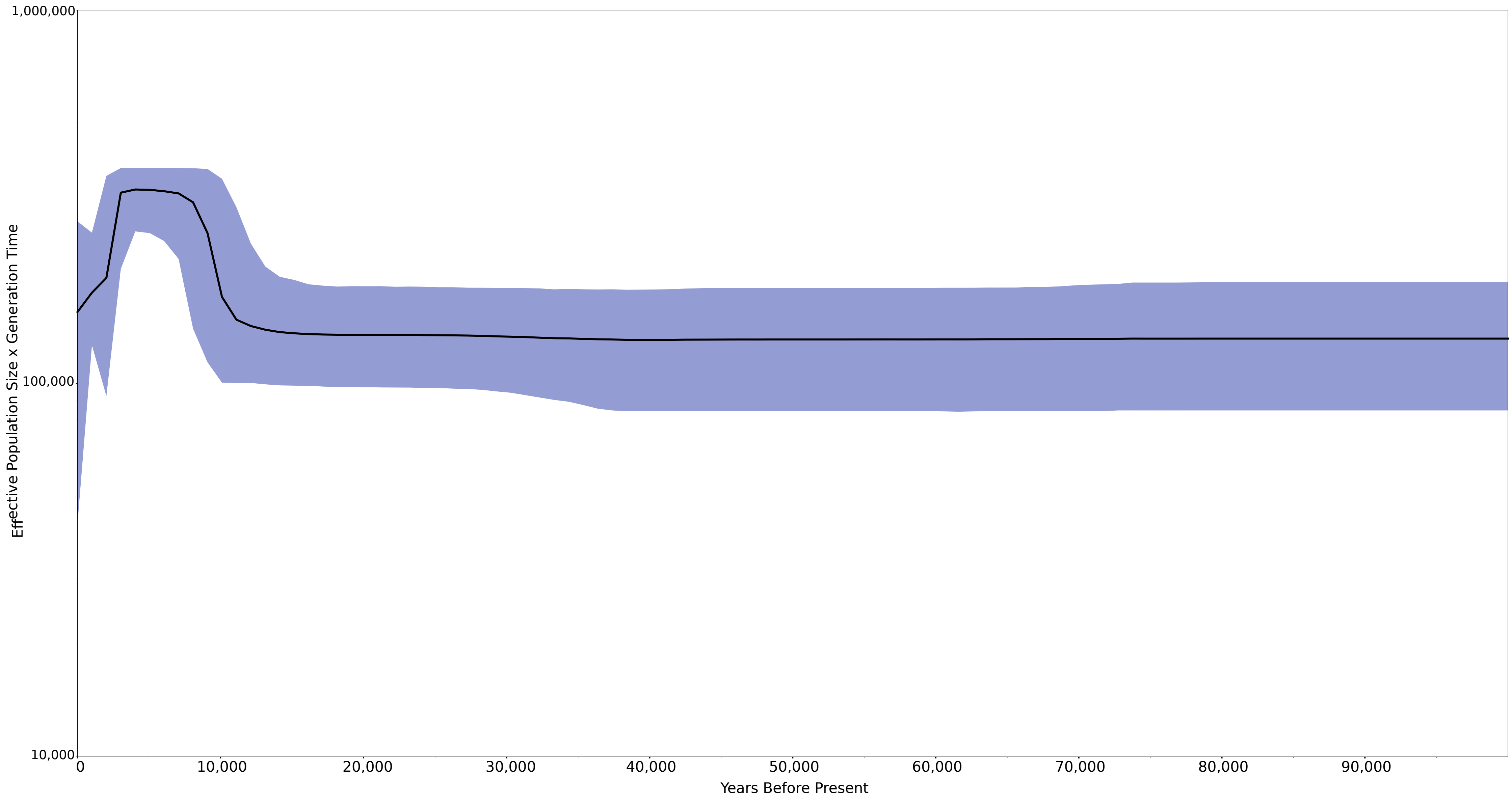

1,000,000
100,000
10,000
Effective Population Size x Generation Time
0
10,000
20,000
30,000
40,000
50,000
60,000
70,000
80,000
90,000
Years Before Present

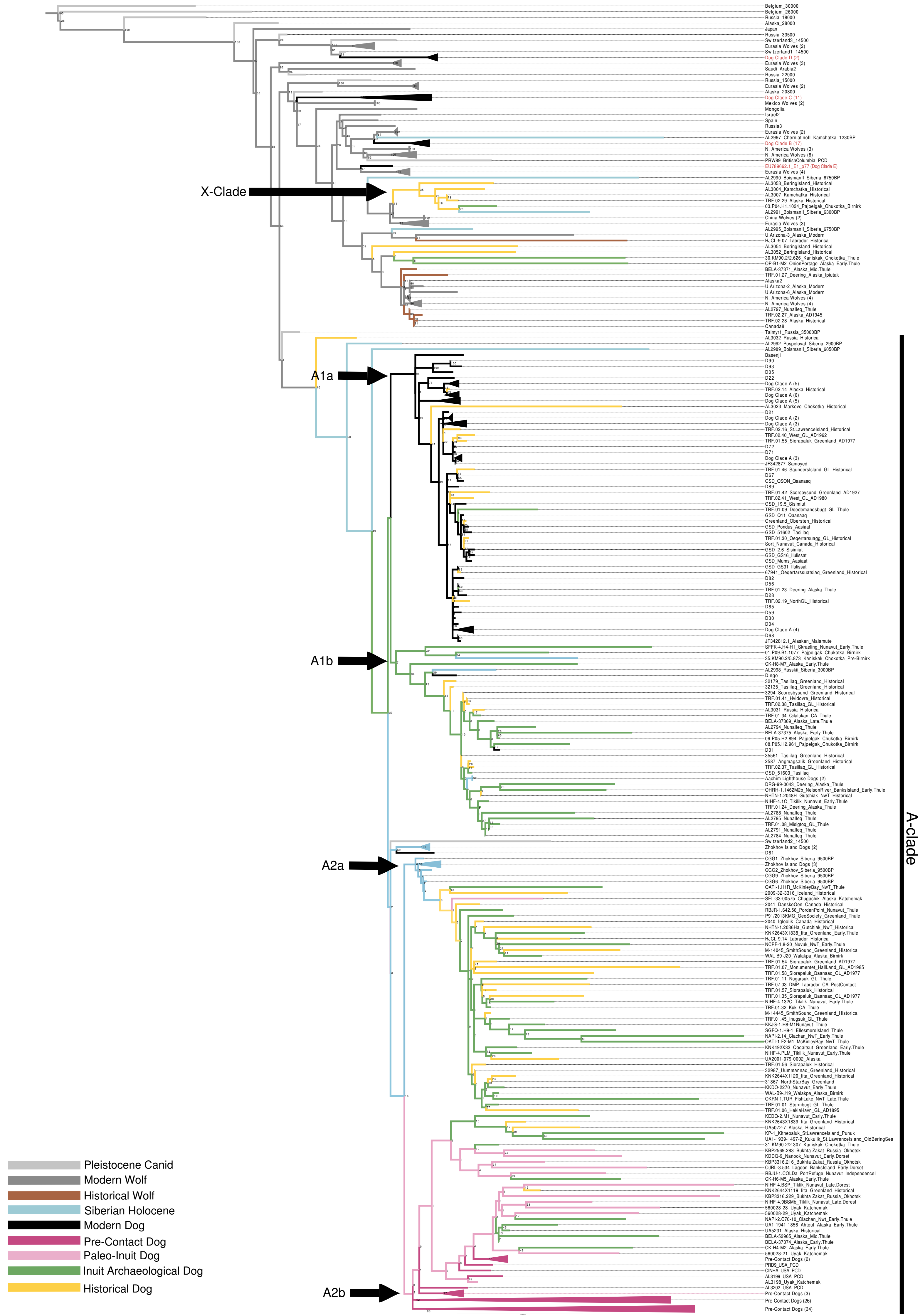
X-Clade
A1a
A1b
A2a
A2b
A-clade
Pleistocene Canid
Modern Wolf
Historical Wolf
Siberian Holocene
Modern Dog
Pre-Contact Dog
Paleo-Inuit Dog
Inuit Archaeological Dog
Historical Dog

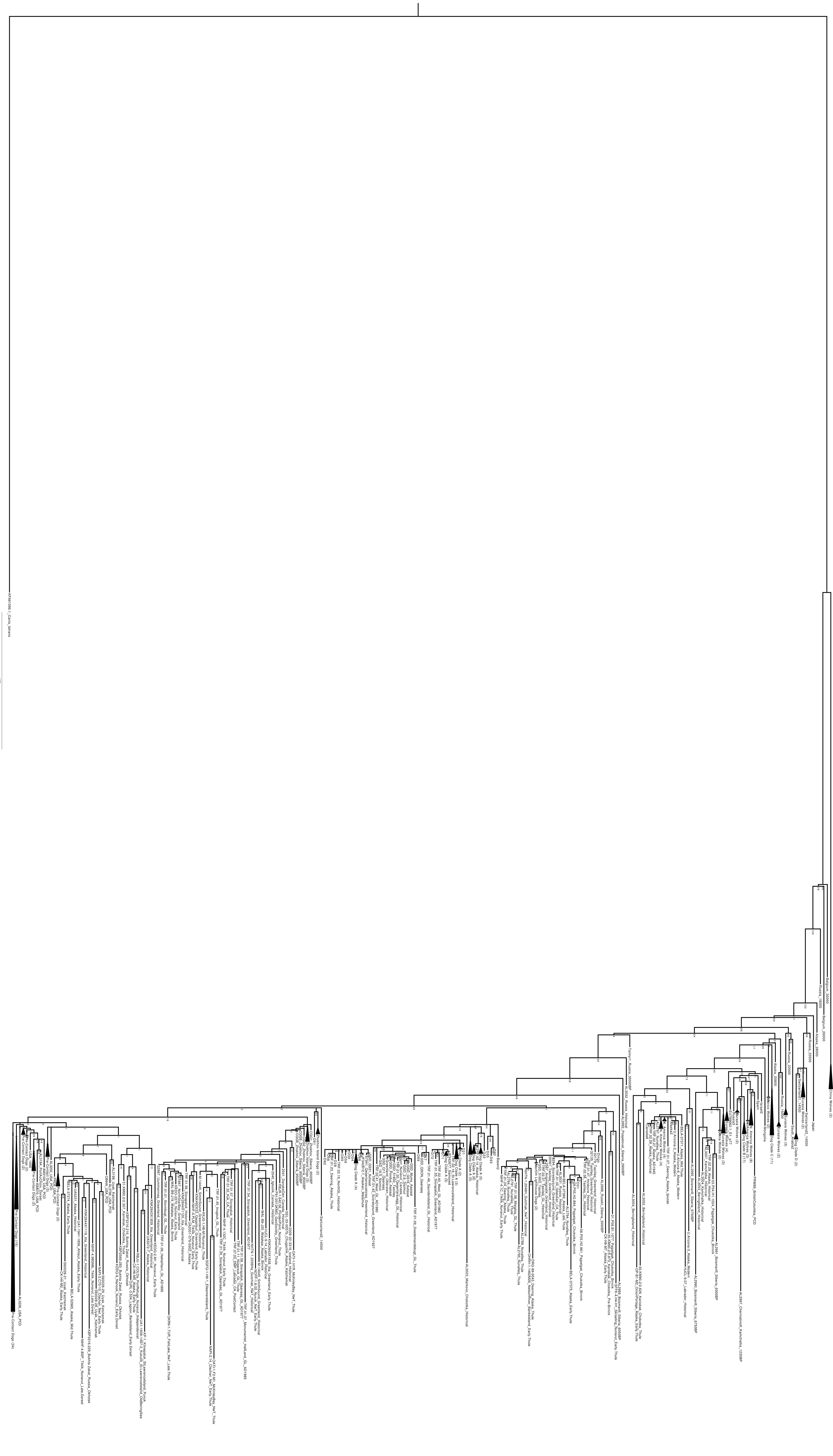

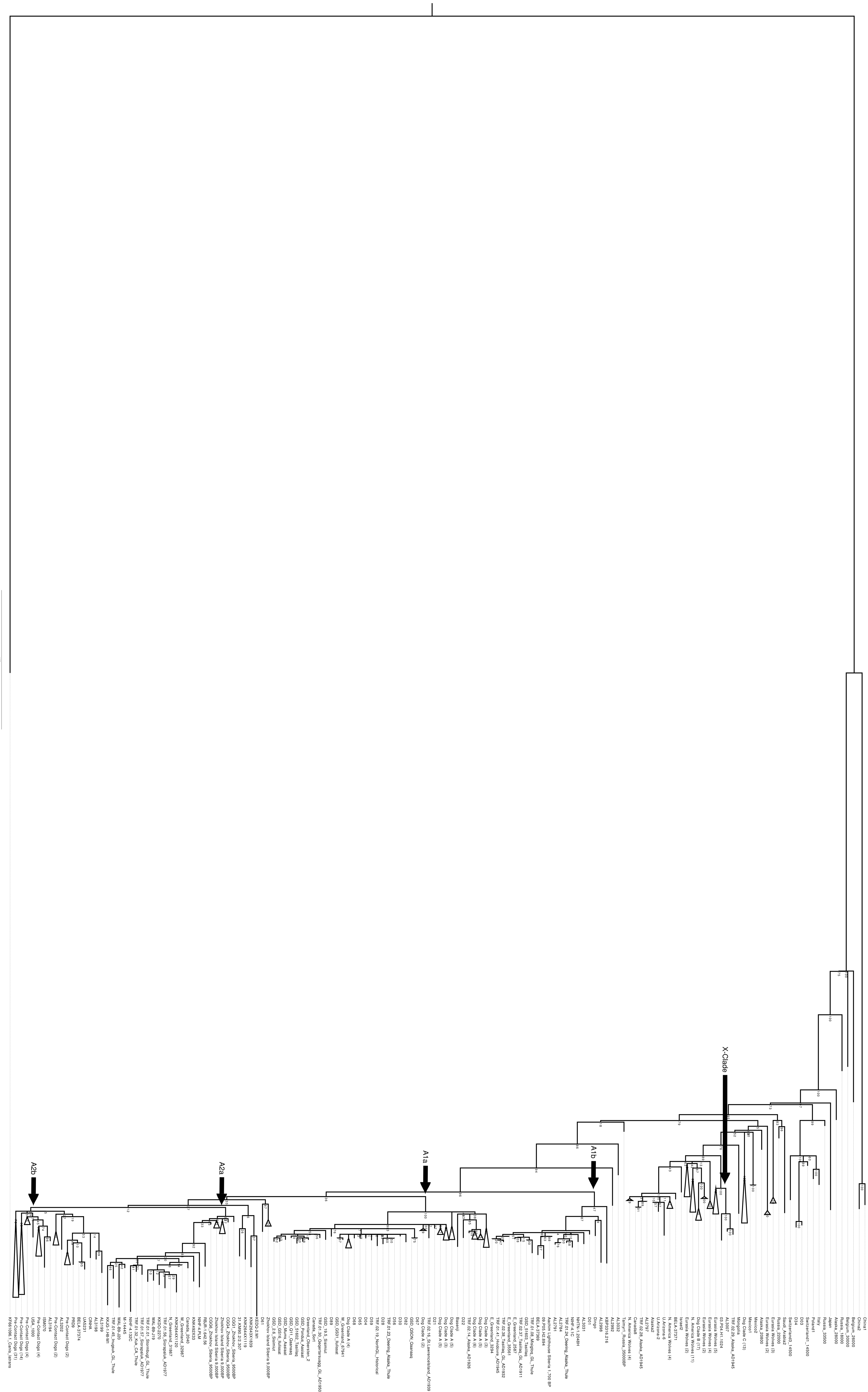
A2b
A2a
A1a
A1b
X-Clade

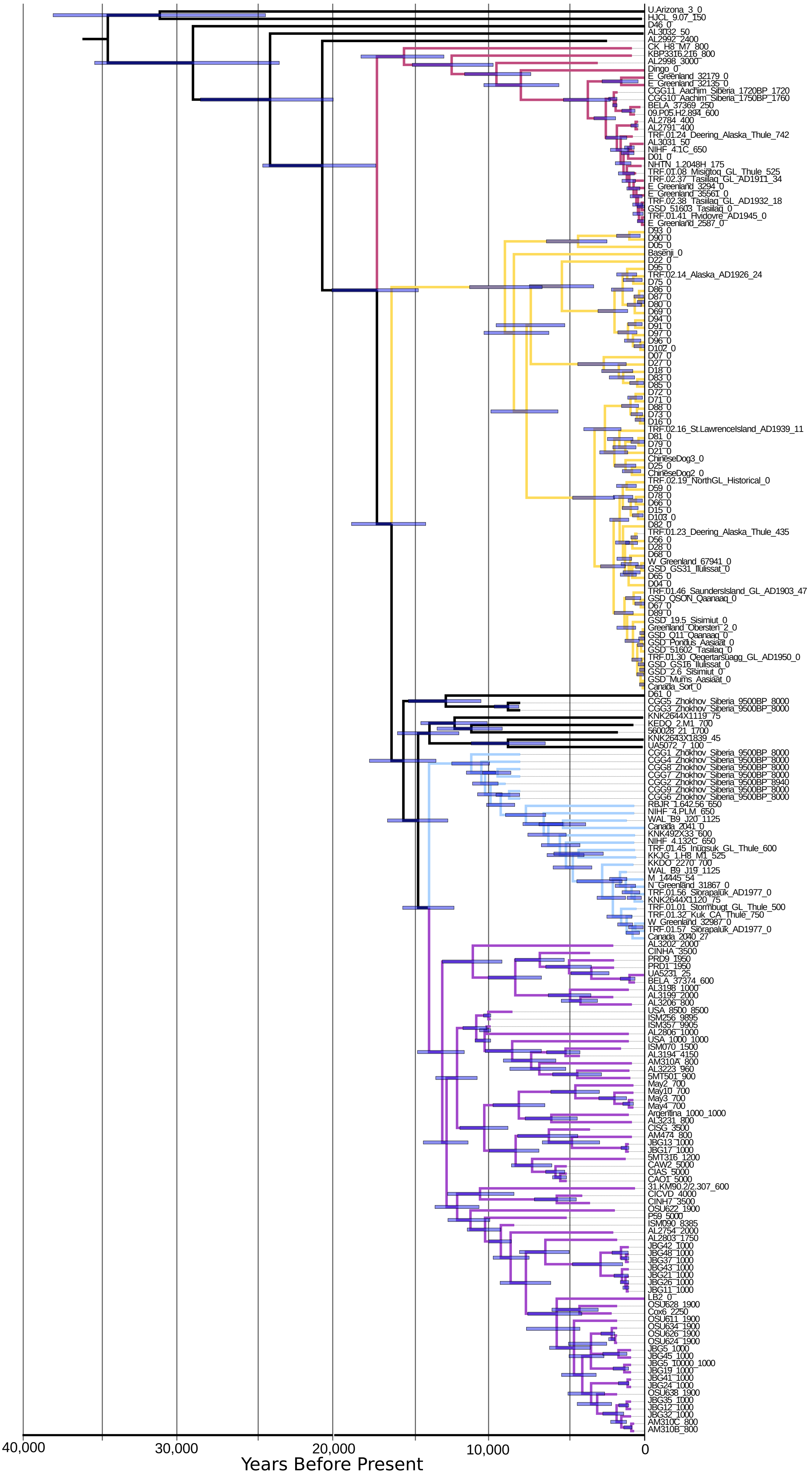

U.Arizona_3_0
HJCL_9.07_150
D46_0
AL3032_50
AL2992_2400
CK_H8_M7_800
KBP3316.216_800
AL2998_3000
Dingo_0
E_Greenland_32179_0
E_Greenland_32135_0
CGG11_Aachim_Siberia_1720BP_1720
CGG10_Aachim_Siberia_1750BP_1760
BELA_37369_250
09.P05.H2.894_600
AL2784_400
AL2791_400
TRF.01.24_Deering_Alaska_Thule_742
AL3031_50
NIHF_4.1C_650
D01_0
NHTN_1.2048H_175
TRF.01.08_Misigtoq_GL_Thule_525
TRF.02.37_Tasiilaq_GL_AD1911_34
E_Greenland_3294_0
E_Greenland_35561_0
TRF.02.38_Tasiilaq_GL_AD1932_18
GSD_51603_Tasiilaq_0
TRF.01.41_Hvidovre_AD1945_0
E_Greenland_2587_0
D93_0
D90_0
D05_0
Basenji_0
D22_0
D95_0
TRF.02.14_Alaska_AD1926_24
D75_0
D86_0
D87_0
D80_0
D69_0
D94_0
D91_0
D97_0
D96_0
D102_0
D07_0
D27_0
D18_0
D83_0
D85_0
D72_0
D71_0
D88_0
D73_0
D16_0
TRF.02.16_St.LawrenceIsland_AD1939_11
D81_0
D79_0
D21_0
ChineseDog3_0
D25_0
ChineseDog2_0
TRF.02.19_NorthGL_Historical_0
D59_0
D78_0
D66_0
D15_0
D103_0
D82_0
TRF.01.23_Deering_Alaska_Thule_435
D56_0
D28_0
D68_0
W_Greenland_67941_0
GSD_GS31_Ilulissat_0
D65_0
D04_0
TRF.01.46_SaundersIsland_GL_AD1903_47
GSD_QSON_Qaanaaq_0
D67_0
D89_0
GSD_19.5_Sisimiut_0
Greenland_Obersten_2_0
GSD_Q11_Qaanaaq_0
GSD_Pondus_Aasiaat_0
GSD_51602_Tasiilaq_0
TRF.01.30_Qeqertarsuagg_GL_AD1950_0
GSD_GS16_Ilulissat_0
GSD_2.6_Sisimiut_0
GSD_Mums_Aasiaat_0
Canada_Sort_0
D61_0
CGG5_Zhokhov_Siberia_9500BP_8000
CGG3_Zhokhov_Siberia_9500BP_8000
KNK2644X1119_75
KEDQ_2.M1_700
560028_21_1700
KNK2643X1839_45
UA5072_7_100
CGG1_Zhokhov_Siberia_9500BP_8000
CGG4_Zhokhov_Siberia_9500BP_8000
CGG8_Zhokhov_Siberia_9500BP_8000
CGG7_Zhokhov_Siberia_9500BP_8000
CGG2_Zhokhov_Siberia_9500BP_8940
CGG9_Zhokhov_Siberia_9500BP_8000
CGG6_Zhokhov_Siberia_9500BP_8000
RBJR_1.642.56_650
NIHF_4.PLM_650
WAL_B9_J20_1125
Canada_2041_0
KNK492X33_600
NIHF_4.132C_650
TRF.01.45_Inugsuk_GL_Thule_600
KKJG_1.H8_M1_525
KKDO_2270_700
WAL_B9_J19_1125
M_14445_54
N_Greenland_31867_0
TRF.01.56_Siorapaluk_AD1977_0
KNK2644X1120_75
TRF.01.01_Stormbugt_GL_Thule_500
TRF.01.32_Kuk_CA_Thule_750
W_Greenland_32987_0
TRF.01.57_Siorapaluk_AD1977_0
Canada_2040_27
AL3202_2000
CINHA_3500
PRD9_1950
PRD1_1950
UA5231_25
BELA_37374_600
AL3198_1000
AL3199_2000
AL3206_800
USA_8500_8500
ISM256_9895
ISM357_9905
AL2806_1000
USA_1000_1000
ISM070_1500
AL3194_4150
AM310A_800
AL3223_960
5MT501_900
May2_700
May10_700
May3_700
May4_700
Argentina_1000_1000
AL3231_800
CISG_3500
AM474_800
JBG13_1000
JBG17_1000
5MT316_1200
CAW2_5000
CIAS_5000
CAOT_5000
31.KM90.2/2.307_600
CICVD_4000
CINH7_3500
OSU622_1900
P59_5000
ISM090_8385
AL2754_2000
AL2803_1750
JBG42_1000
JBG48_1000
JBG37_1000
JBG43_1000
JBG21_1000
JBG26_1000
JBG11_1000
LB2_0
OSU628_1900
Cox6_2250
OSU611_1900
OSU634_1900
OSU626_1900
OSU624_1900
JBG5_1000
JBG45_1000
JBG5_10000_1000
JBG19_1000
JBG41_1000
JBG24_1000
OSU638_1900
JBG35_1000
JBG12_1000
JBG32_1000
AM310C_800
AM310B_800
40,000
30,000
20,000
10,000
0
Years Before Present

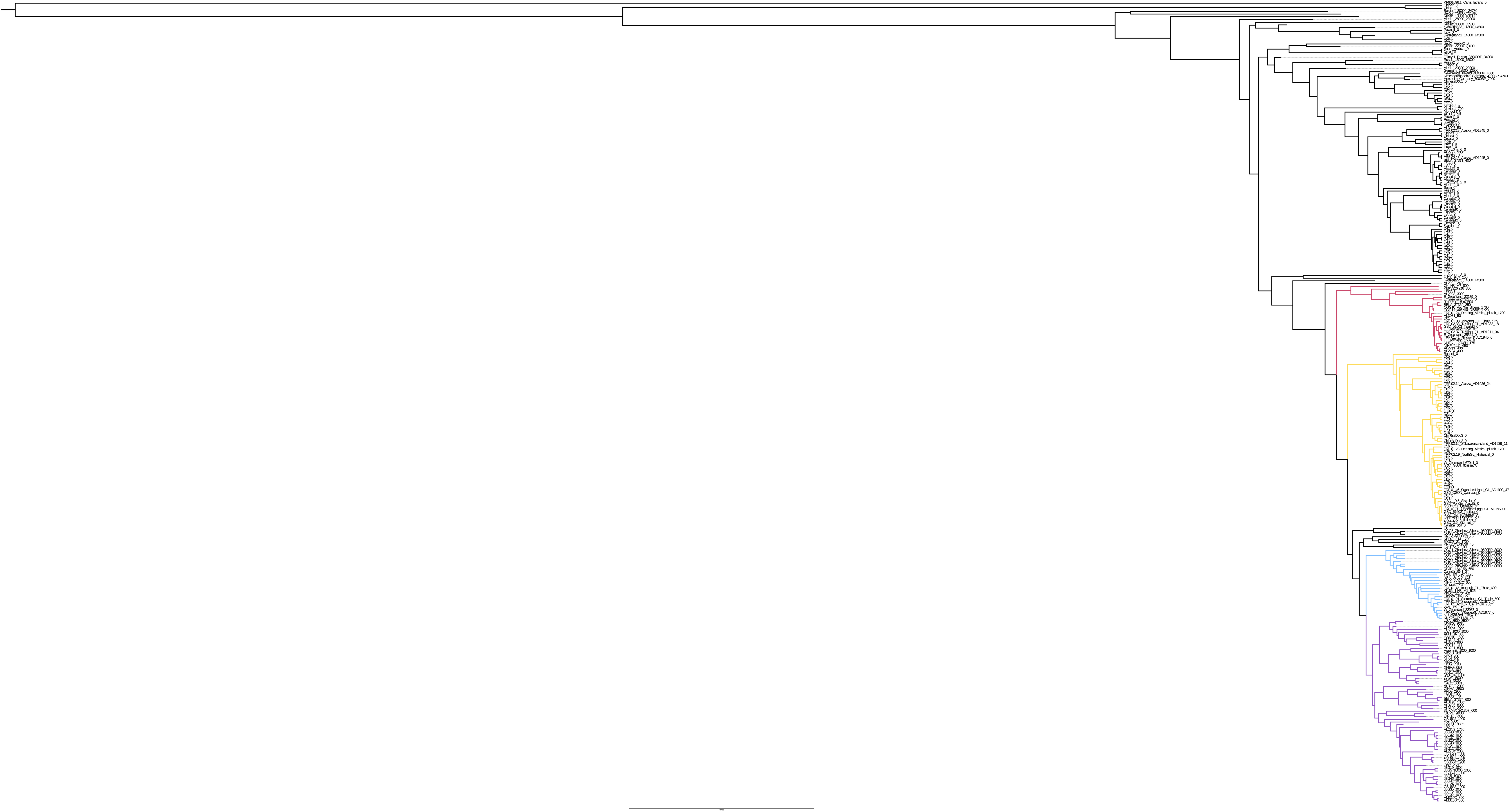

Bayesian Skyline Plots

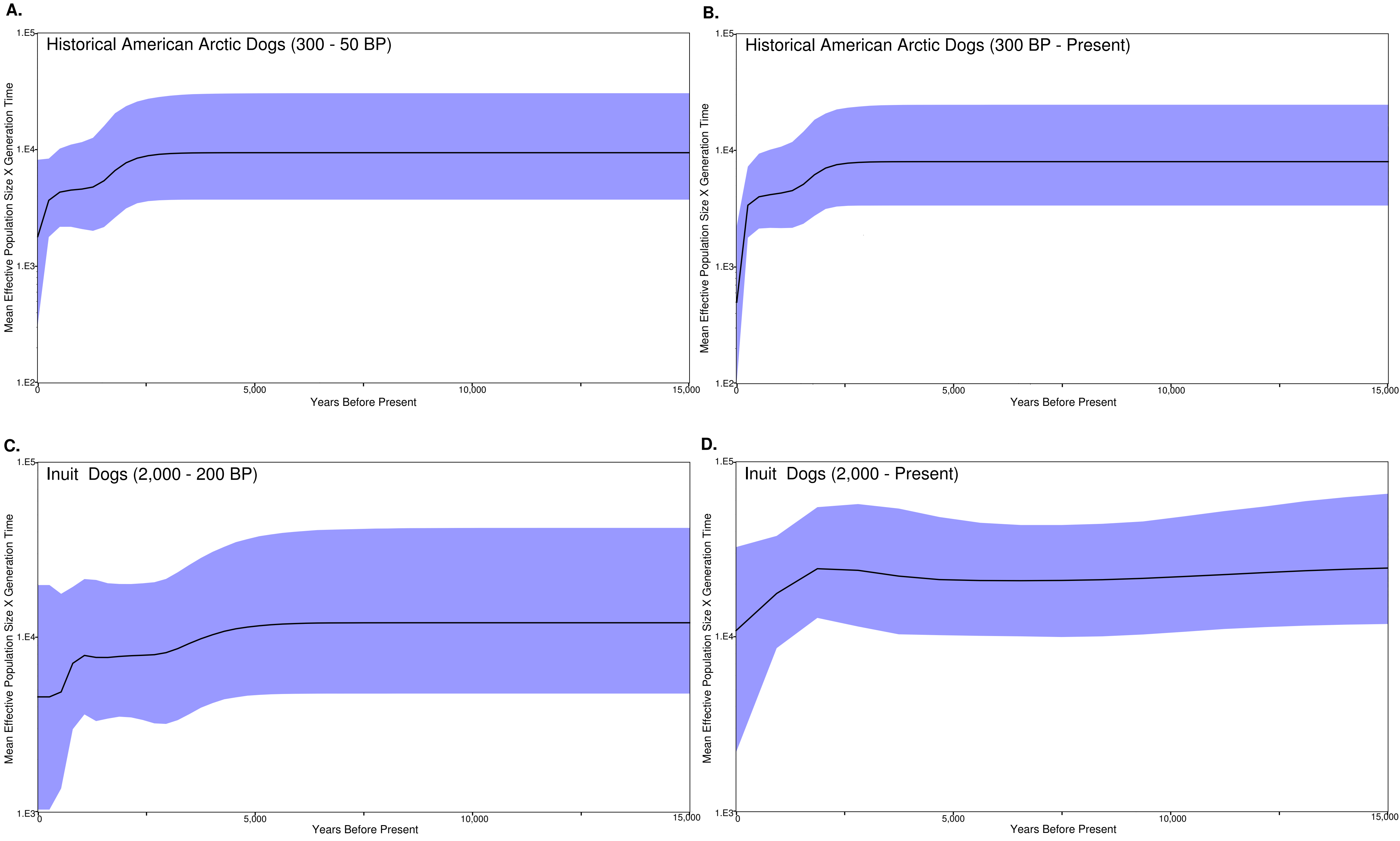

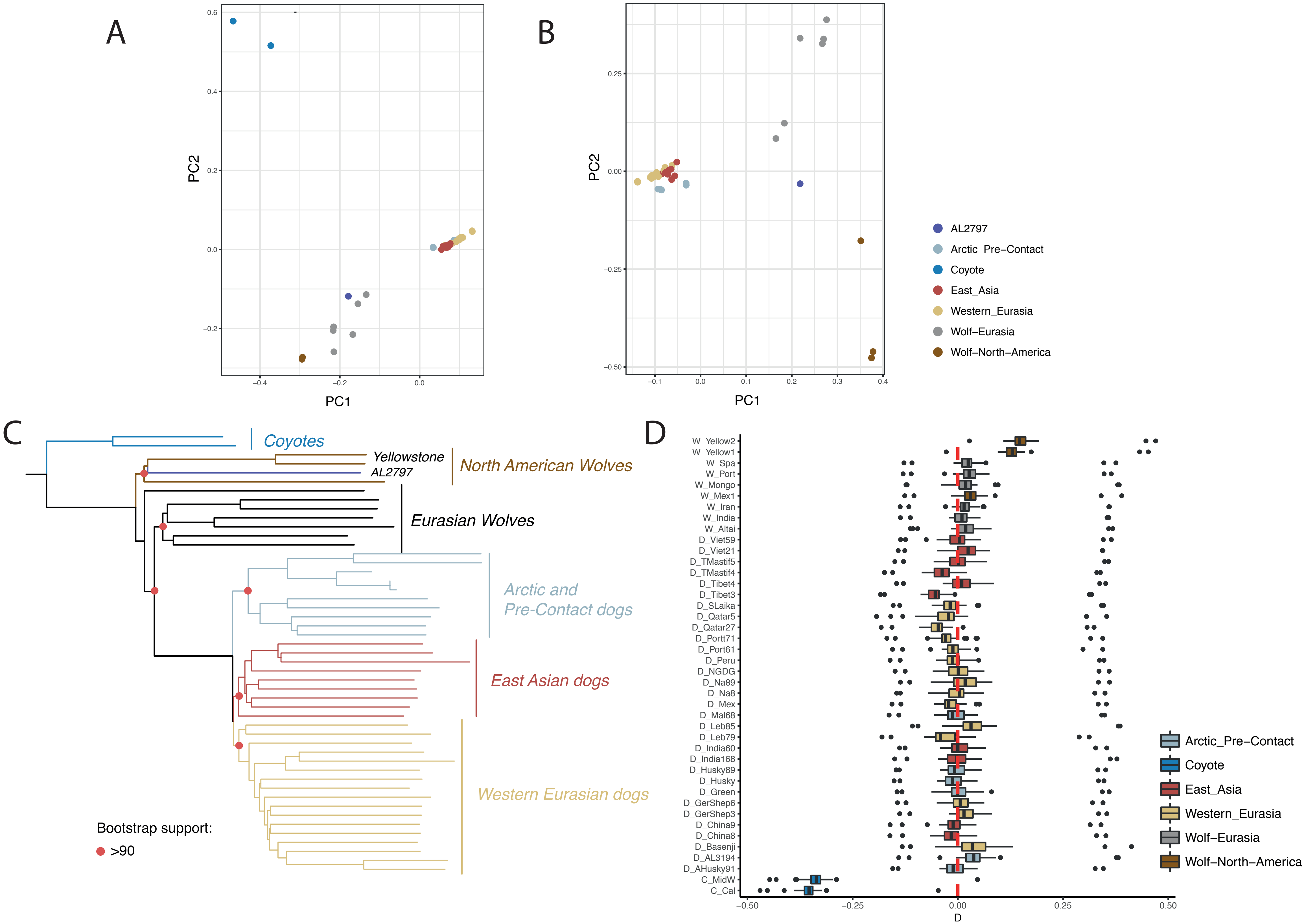

A
B
C
D
PC1
PC2
AL2797
Arctic_Pre−Contact
Coyote
East_Asia
Western_Eurasia
Wolf−Eurasia
Wolf−North−America
Coyotes
Yellowstone
AL2797
North American Wolves
Eurasian Wolves
Arctic and Pre-Contact dogs
East Asian dogs
Western Eurasian dogs
Bootstrap support:
>90
W_Yellow2
W_Yellow1
W_Spa
W_Port
W_Mongo
W_Mex1
W_Iran
W_India
W_Altai
D_Viet59
D_Viet21
D_TMastif5
D_TMastif4
D_Tibet4
D_Tibet3
D_SLaika
D_Qatar5
D_Qatar27
D_Portt71
D_Port61
D_Peru
D_NGDG
D_Na89
D_Na8
D_Mex
D_Mal68
D_Leb85
D_Leb79
D_India60
D_India168
D_Husky89
D_Husky
D_Green
D_GerShep6
D_GerShep3
D_China9
D_China8
D_Basenji
D_AL3194
D_AHusky91
C_MidW
C_Cal
D

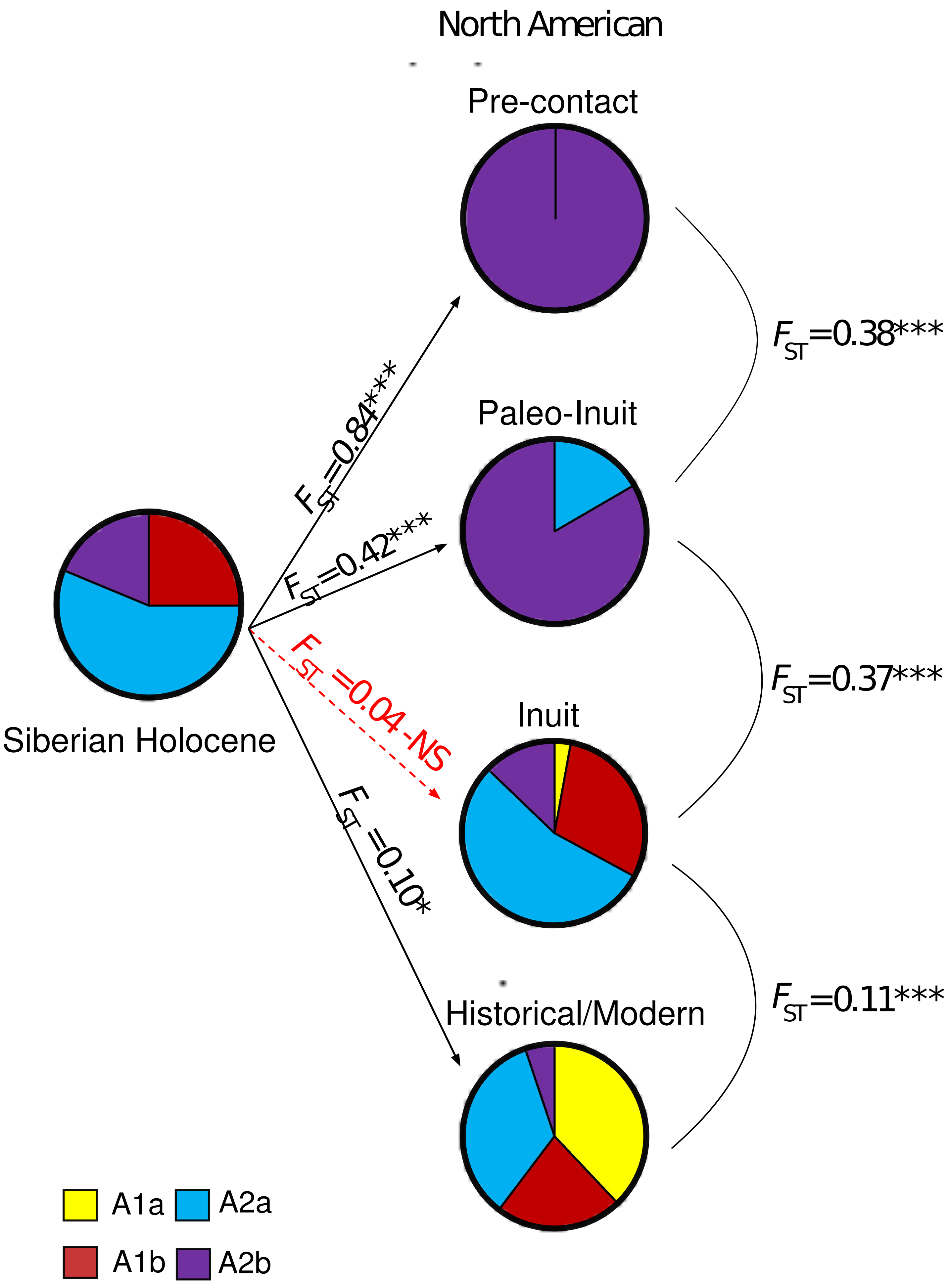
North American
Pre-contact
Paleo-Inuit
Inuit
Historical/Modern
Siberian Holocene
F_{ST}=0.84***
F_{ST}=0.42***
F_{ST} =0.04-NS
F_{ST} =0.10*
F_{ST}=0.38***
F_{ST}=0.37***
F_{ST}=0.11***
A1a
A2a
A1b
A2b

A.

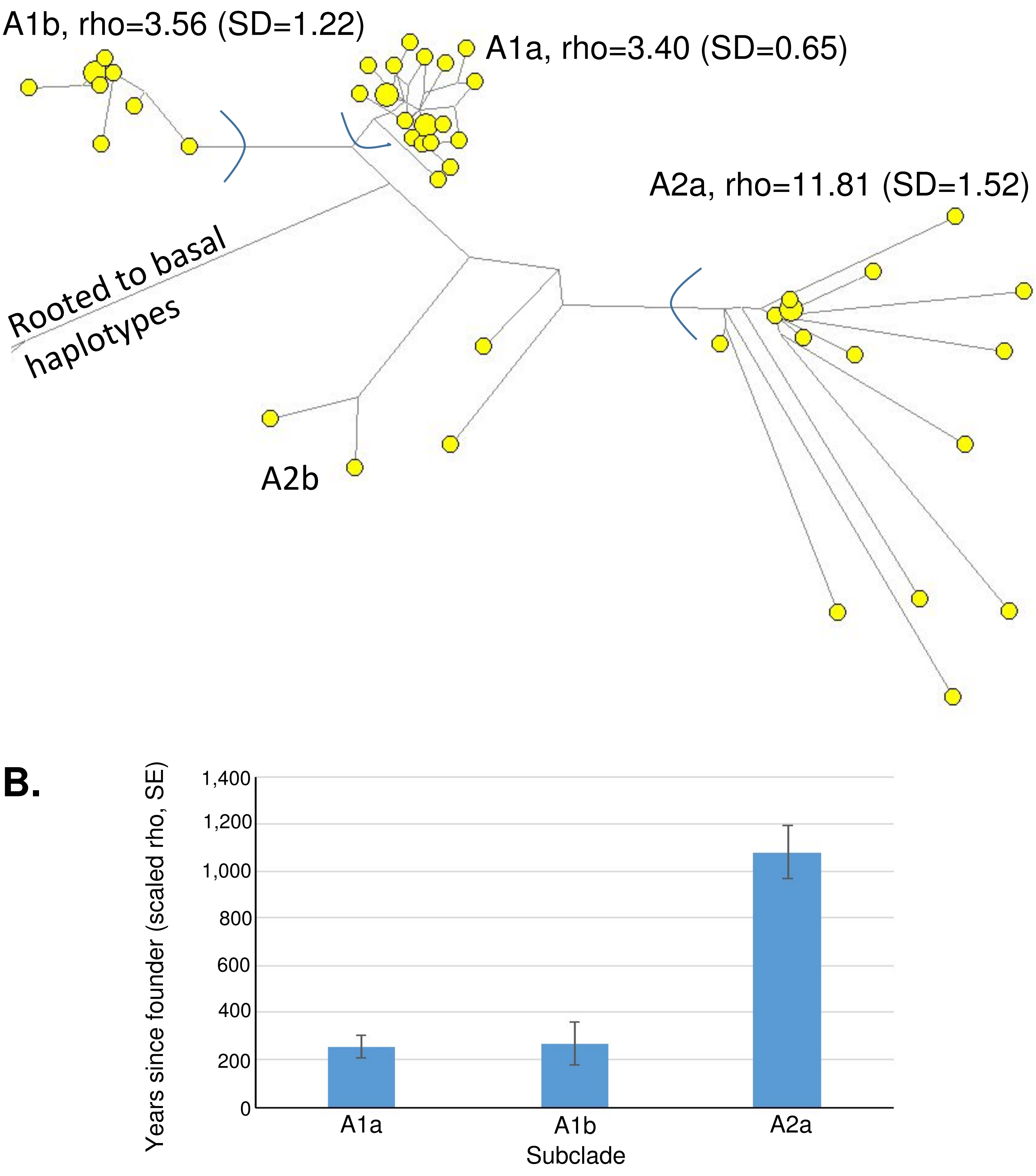

A1b, rho=3.56 (SD=1.22)
A1a, rho=3.40 (SD=0.65)
A2a, rho=11.81 (SD=1.52)
Rooted to basal haplotypes
A2b
B.
Years since founder (scaled rho, SE)
1,400
1,200
1,000
800
600
400
200
0
A1a
A1b
A2a
Subclade

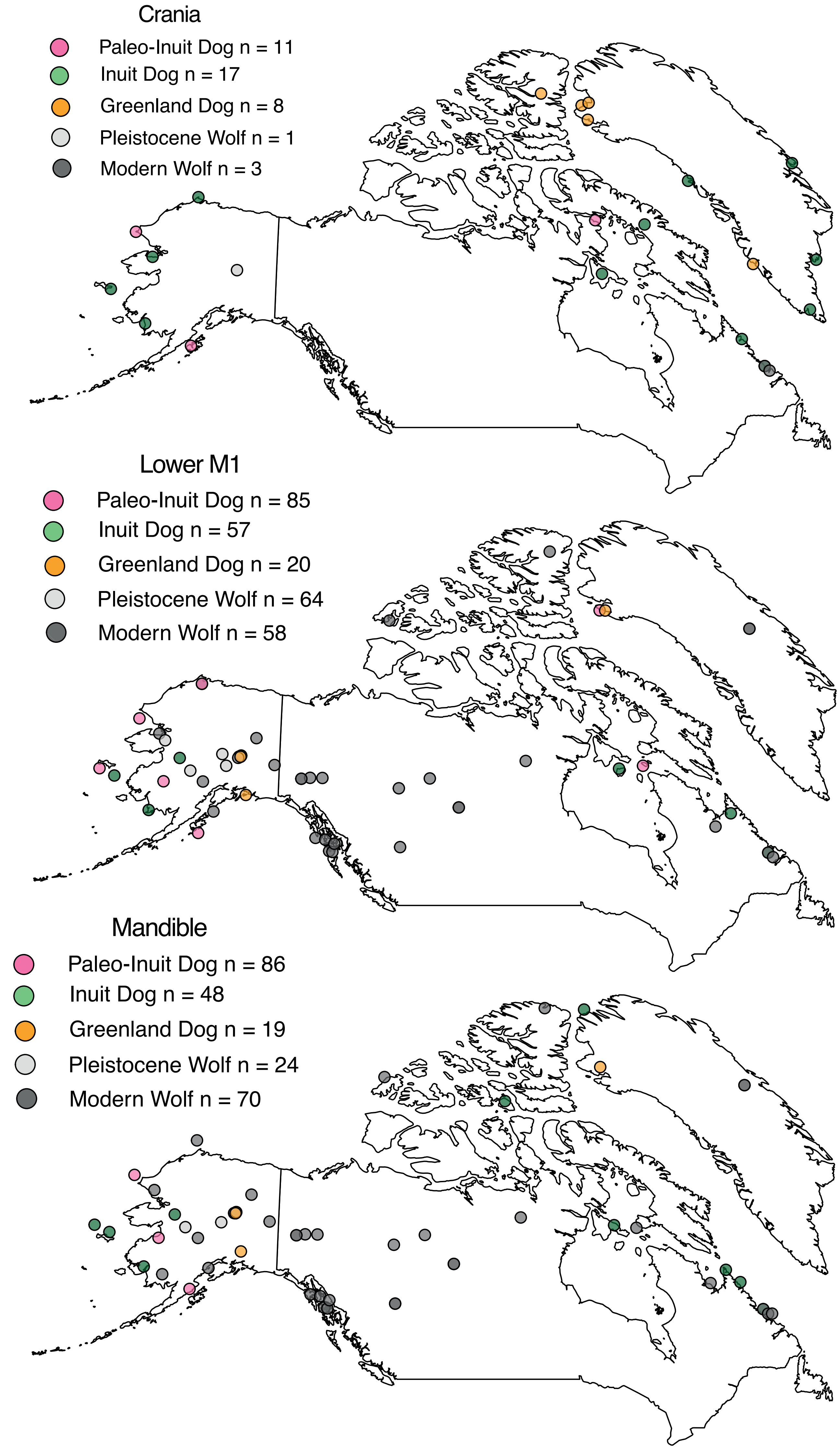
Crania
Paleo-Inuit Dog n = 11
Inuit Dog n = 17
Greenland Dog n = 8
Pleistocene Wolf n = 1
Modern Wolf n = 3
Lower M1
Paleo-Inuit Dog n = 85
Inuit Dog n = 57
Greenland Dog n = 20
Pleistocene Wolf n = 64
Modern Wolf n = 58
Mandible
Paleo-Inuit Dog n = 86
Inuit Dog n = 48
Greenland Dog n = 19
Pleistocene Wolf n = 24
Modern Wolf n = 70

Crania

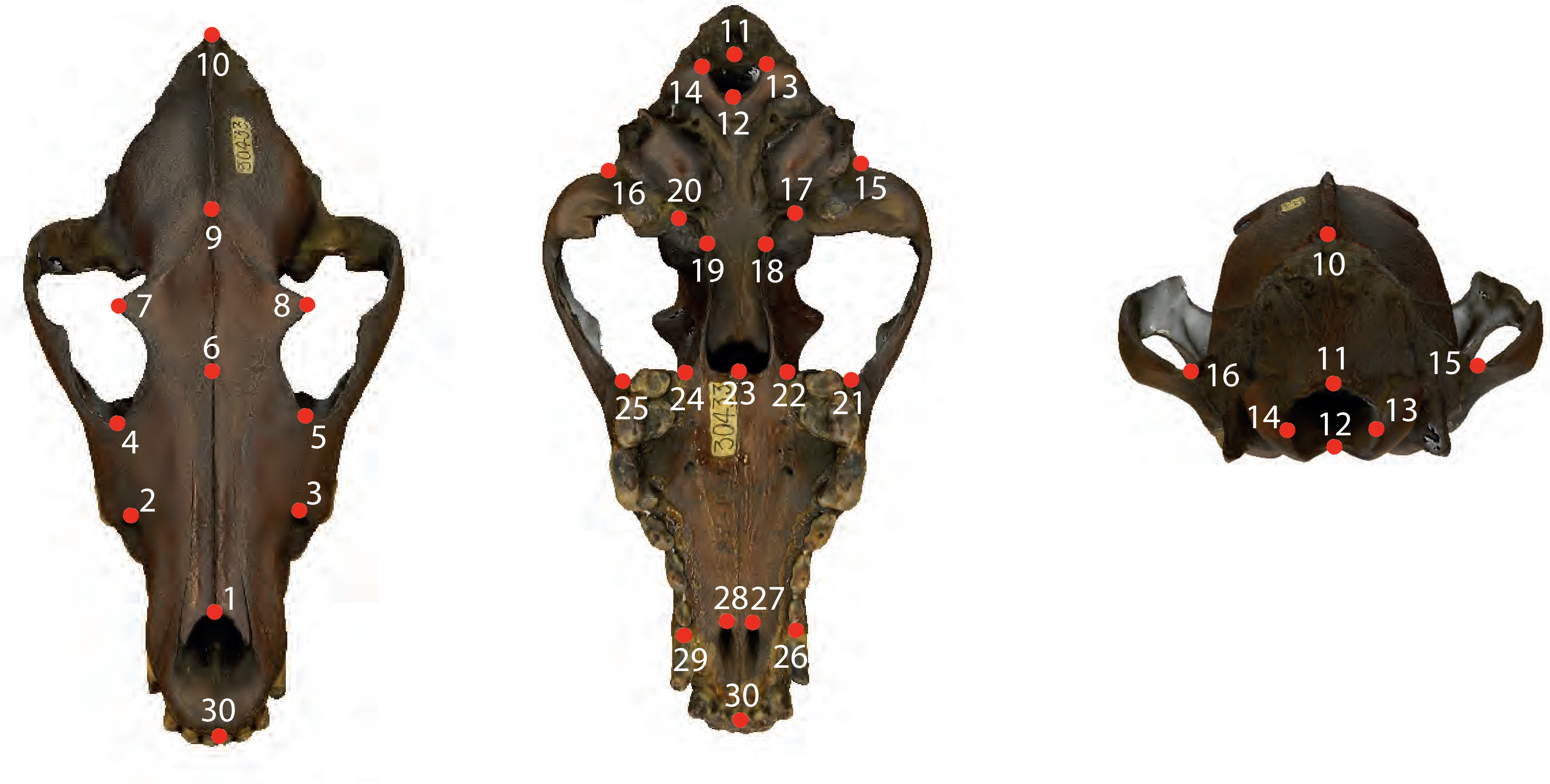

Lower M1

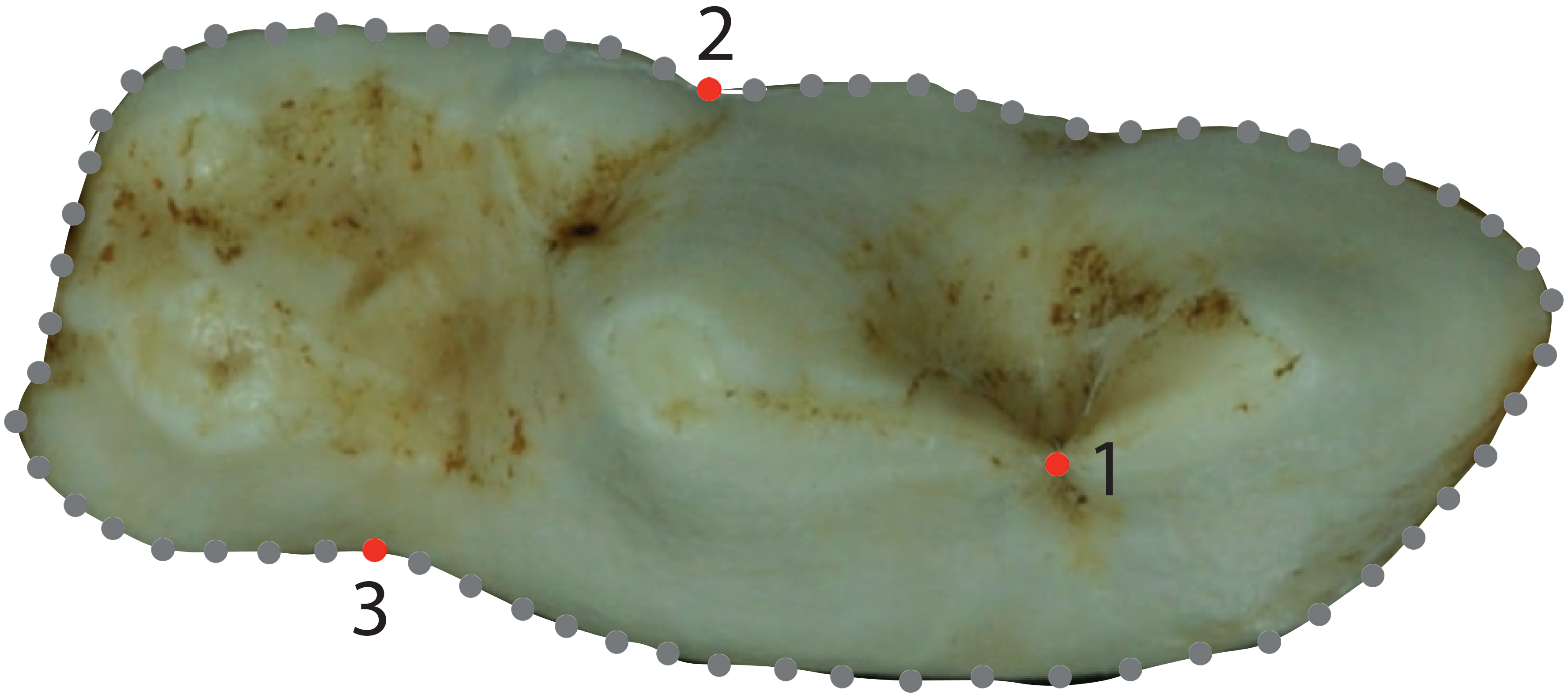

Mandible

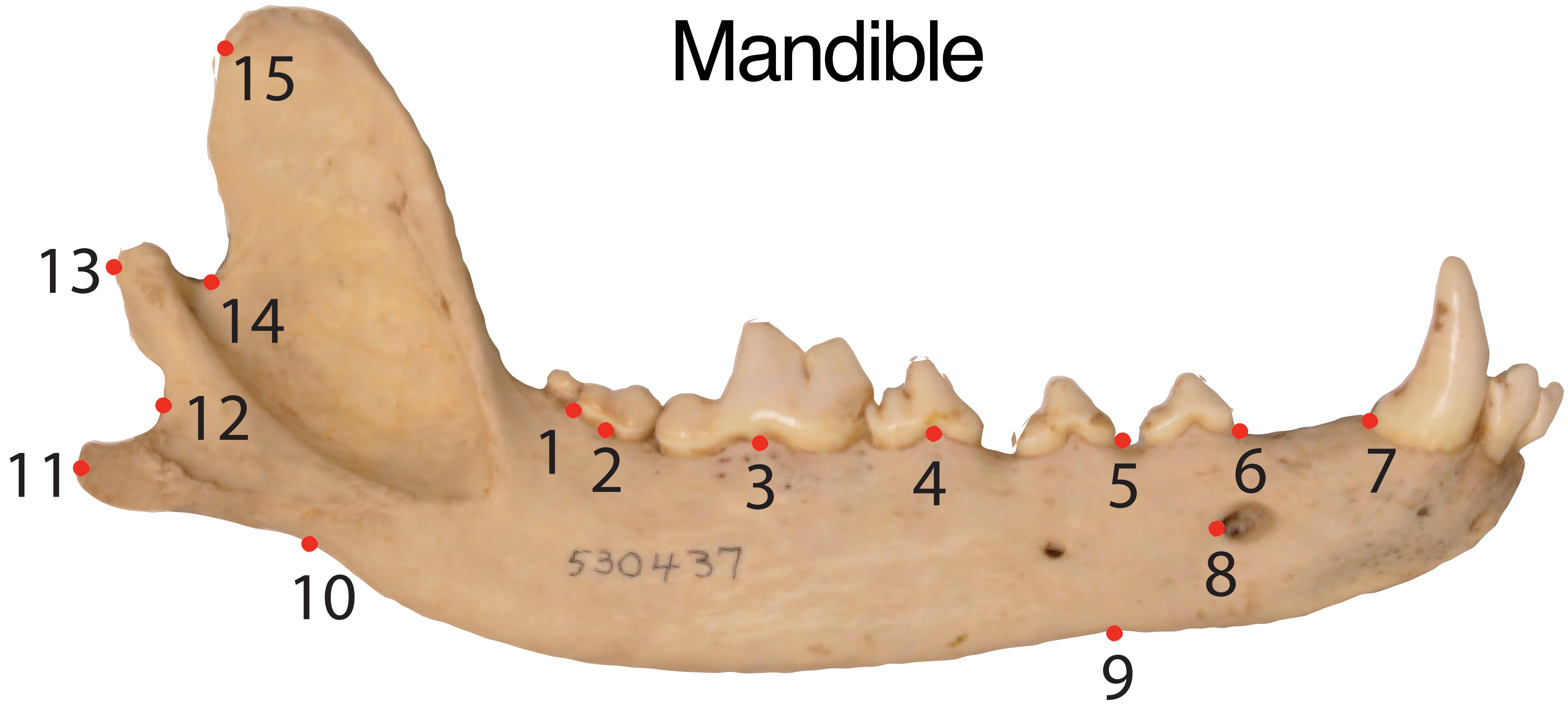

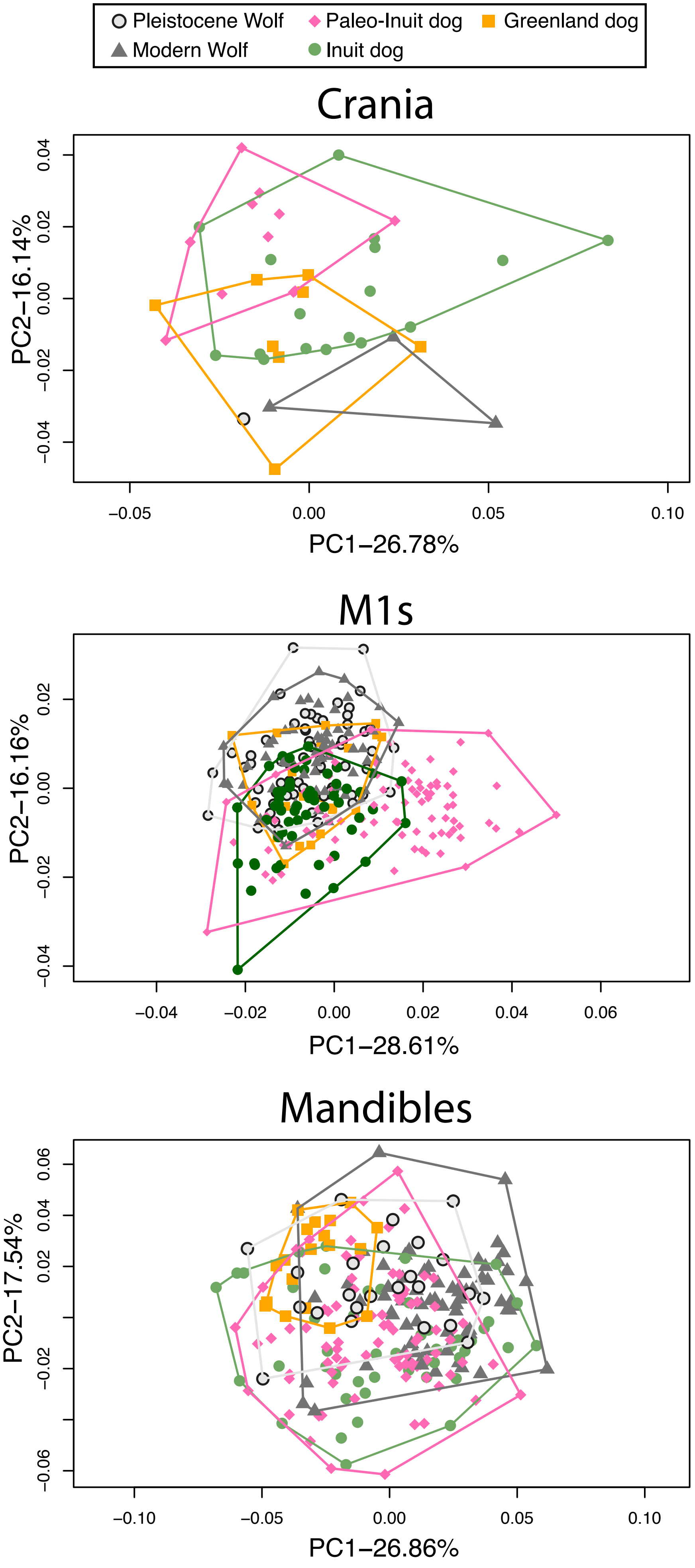
Pleistocene Wolf
Paleo-Inuit dog
Greenland dog
Modern Wolf
Inuit dog
Crania
PC2−16.14%
0.04
0.02
0.00
−0.02
−0.04
−0.05
0.00
0.05
0.10
PC1−26.78%
M1s
PC2−16.16%
0.02
0.00
−0.02
−0.04
−0.04
−0.02
0.00
0.02
0.04
0.06
PC1−28.61%
Mandibles
PC2−17.54%
0.06
0.04
0.02
−0.02
−0.06
−0.10
−0.05
0.00
0.05
0.10
PC1−26.86%

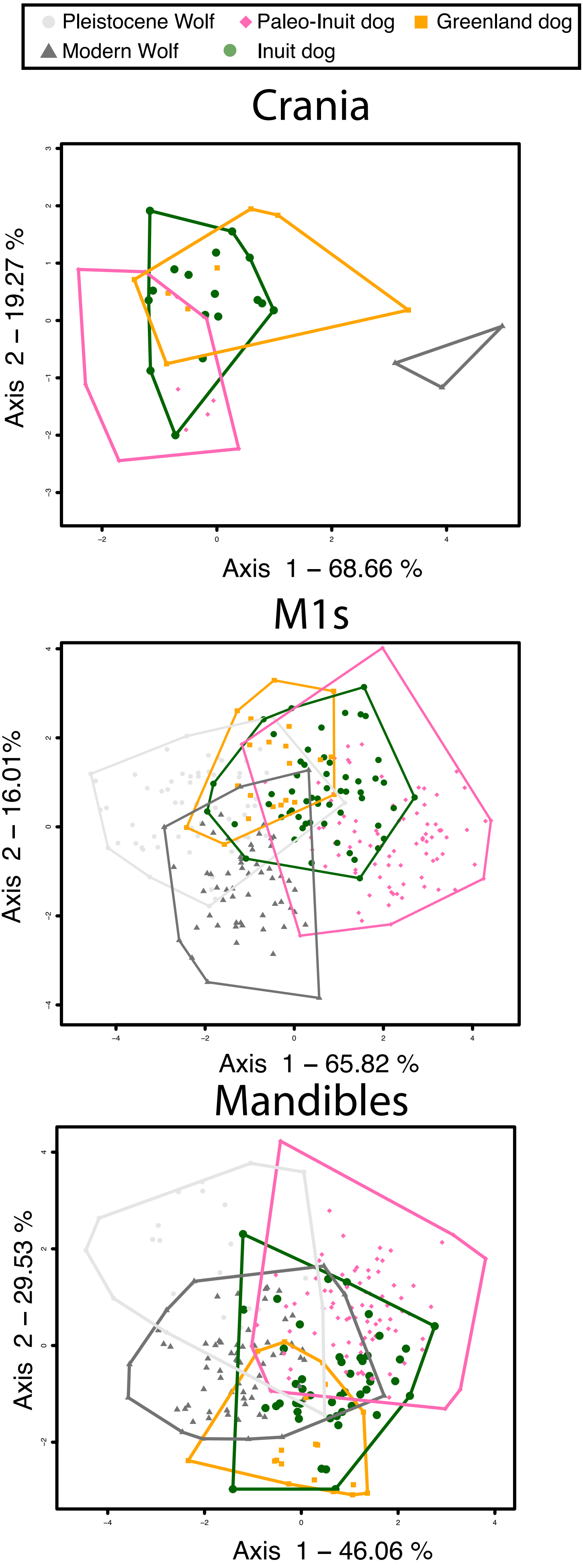
Pleistocene Wolf
Paleo-Inuit dog
Greenland dog
Modern Wolf
Inuit dog
Crania
Axis 2 – 19.27 %
Axis 1 – 68.66 %
M1s
Axis 2 – 16.01%
Axis 1 – 65.82 %
Mandibles
Axis 2 – 29.53 %
Axis 1 – 46.06 %

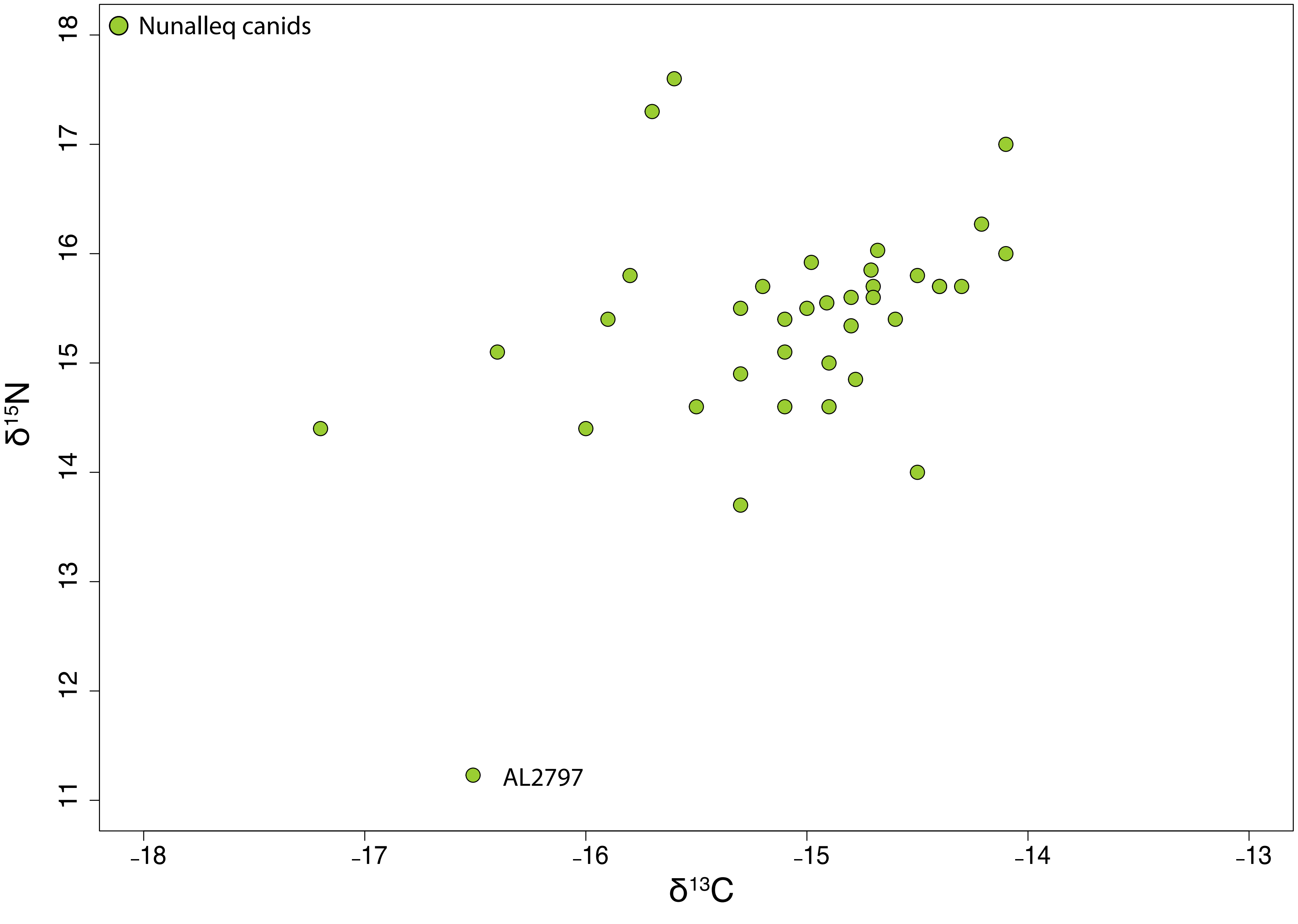

Nunalleq canids
AL2797
δ15N
δ13C
18
17
16
15
14
13
12
11
–18
–17
–16
–15
–14
–13
